# Intestinal stem cell overproliferation resulting from inactivation of the APC tumor suppressor requires the transcription cofactors Earthbound and Erect wing

**DOI:** 10.1371/journal.pgen.1006870

**Published:** 2017-07-14

**Authors:** Ai Tian, Hassina Benchabane, Zhenghan Wang, Chloe Zimmerman, Nan Xin, Jessica Perochon, Gabriela Kalna, Owen J. Sansom, Chao Cheng, Julia B. Cordero, Yashi Ahmed

**Affiliations:** 1 Department of Molecular and Systems Biology and the Norris Cotton Cancer Center, Geisel School of Medicine at Dartmouth College, Hanover, NH, United States of America; 2 Wolfson Wohl Research Centre, Institute of Cancer Sciences, University of Glasgow, Glasgow, United Kingdom; 3 CRUK Beatson Institute, Garscube Estate, Glasgow, United Kingdom; 4 CRUK Beatson Institute, Institute of Cancer Sciences, University of Glasgow, Garscube Estate, Glasgow, United Kingdom; 5 Department of Biomedical Data Science, Molecular and Systems Biology and the Norris Cotton Cancer Center, Geisel School of Medicine at Dartmouth College, Hanover, NH, United States of America; The University of North Carolina at Chapel Hill, UNITED STATES

## Abstract

Wnt/β-catenin signal transduction directs intestinal stem cell (ISC) proliferation during homeostasis. Hyperactivation of Wnt signaling initiates colorectal cancer, which most frequently results from truncation of the tumor suppressor Adenomatous polyposis coli (APC). The β-catenin-TCF transcription complex activates both the physiological expression of Wnt target genes in the normal intestinal epithelium and their aberrantly increased expression in colorectal tumors. Whether mechanistic differences in the Wnt transcription machinery drive these distinct levels of target gene activation in physiological versus pathological states remains uncertain, but is relevant for the design of new therapeutic strategies. Here, using a Drosophila model, we demonstrate that two evolutionarily conserved transcription cofactors, Earthbound (Ebd) and Erect wing (Ewg), are essential for all major consequences of *Apc1* inactivation in the intestine: the hyperactivation of Wnt target gene expression, excess number of ISCs, and hyperplasia of the epithelium. In contrast, only Ebd, but not Ewg, mediates the Wnt-dependent regulation of ISC proliferation during homeostasis. Therefore, in the adult intestine, Ebd acts independently of Ewg in physiological Wnt signaling, but cooperates with Ewg to induce the hyperactivation of Wnt target gene expression following *Apc1* loss. These findings have relevance for human tumorigenesis, as Jerky (JRK/JH8), the human Ebd homolog, promotes Wnt pathway hyperactivation and is overexpressed in colorectal, breast, and ovarian cancers. Together, our findings reveal distinct requirements for Ebd and Ewg in physiological Wnt pathway activation versus oncogenic Wnt pathway hyperactivation following *Apc1* loss. Such differentially utilized transcription cofactors may offer new opportunities for the selective targeting of Wnt-driven cancers.

## Author summary

The identification of effective therapy for colorectal cancer, which is a leading cause of cancer-related death, is imperative. Wnt pathway components have promise as therapeutic targets, since more than 90% of colon cancers are triggered by mutations that overactivate this pathway, particularly in the tumor suppressor *APC*. However, as Wnt signaling is also required for normal intestinal homeostasis, the selective therapeutic targeting of oncogenic Wnt signaling remains a major challenge. Through a forward genetic screen, we previously identified two suppressors of Drosophila *Apc1*, Earthbound (Ebd) and Erect wing (Ewg), as transcription cofactors of the Wnt pathway. Here, we analyze the roles of these two factors in the Wnt-dependent control of intestinal stem cell proliferation. We find that both Ebd and Ewg are essential for the hyperactivation of Wnt signaling and the consequent epithelial hyperplasia resulting from *Apc1* inactivation. Moreover, Ebd, but not Ewg, is also required for the Wnt-dependent maintenance of normal intestinal homeostasis. Together, our findings reveal differential requirements for two highly conserved transcriptional cofactors in Wnt pathway activation versus hyperactivation. The identification of such factors may provide potential selectivity for the targeting of Wnt-driven cancers.

## Introduction

The evolutionarily conserved Wnt/β-catenin signal transduction pathway directs fundamental cellular processes across metazoans, whereas deregulation of this pathway is associated with numerous human congenital disorders and cancers [1,2]. In the absence of Wnt exposure, β-catenin, a key transcription coactivator, is phosphorylated and targeted for proteasomal degradation by a “destruction complex” comprised of the scaffold protein Axin, the tumor suppressor Adenomatous polyposis coli (APC), and two kinases: glycogen synthase kinase 3 (GSK3) and casein kinase 1α (CK1α). Wnt stimulation inactivates the destruction complex and thereby stabilizes β-catenin, which subsequently translocates to the nucleus and interacts with the DNA-binding transcription factor T-cell factor (TCF) to regulate Wnt target genes [3–5].

The adult mammalian intestine is among the many tissues in which Wnt pathway activation is crucial. Wnt signaling is a key determinant of intestinal stem cell (ISC) maintenance and proliferation during homeostasis [6–10]. Conversely, aberrant activation of the Wnt pathway, which occurs primarily through truncating mutations in *APC*, initiates the development of the vast majority of colorectal cancers [10–19]. As inhibition of Wnt signaling blocks the division and induces the differentiation of these cancer cells, targeting Wnt pathway components is of great interest for colorectal cancer treatment [20–23].

Many of the same Wnt target genes that are transcriptionally activated in the intestinal epithelium during homeostasis are expressed at aberrantly increased levels in colorectal tumors harboring mutations in *APC* [18,23–31]. The β-catenin-TCF complex is required for the activation of Wnt target genes in both physiological settings and in these pathological states [7,15,23,32]; however, recent studies have suggested that some of the transcription cofactors interacting with β-catenin-TCF to drive Wnt target gene expression in these two contexts are distinct. For example, B-cell CLL/lymphoma 9 (BCL9) and Pygopus (Pygo), which form a complex with β-catenin and TCF [33–38], are essential only in a subset of tissues during mammalian development [39–45], and are dispensable for Wnt-dependent ISC proliferation and maintenance during homeostasis [40,46]. In contrast, BCL9 and its homolog BCL9-2 are crucial for Wnt-driven intestinal tumor progression [46–52], and Pygo is required for the activation of several Wnt target genes in colon cancer cells [37,47]. These studies suggest that distinct transcription cofactors are utilized in physiological versus pathological states, thereby conferring potential selectivity between Wnt-dependent cell proliferation in normal tissues and tumors. The identification of such novel cofactors that specifically transduce oncogenic Wnt signaling may yield new strategies for the targeting of Wnt-driven cancers.

Through a forward genetic modifier screen for suppressors of *Apc1* in the Drosophila retina, we identified Earthbound1 (Ebd1) and Erect wing (Ewg) as context-specific transcription cofactors in the Wingless pathway [53,54]. Ebd1, a member of a protein family containing Centromere Binding Protein B (CENPB) DNA binding domains, physically associates with and bridges β-catenin/Armadillo (Arm) and TCF, thereby promoting the formation and stability of the β-catenin-TCF complex and the recruitment of β-catenin to chromatin [53]. Ewg is a DNA binding transcriptional activator that shares DNA binding specificity with its human homolog, Nuclear Respiratory Factor-1 (NRF-1) [54–57]. We found that Ewg is a physical and functional partner of Ebd1 that promotes the recruitment of Ebd1 to specific chromatin sites [54]. We postulated that recruitment of Ebd1 to chromatin by Ewg enhances the transcriptional activity of the β-catenin-TCF complex, thus promoting Wingless signaling.

Herein, we report that these two Wnt pathway transcription cofactors have distinct functions in the Wnt-directed regulation of the adult Drosophila intestine. Similar to the mammalian intestine, the adult Drosophila midgut undergoes rapid turnover and is replenished by intestinal stem cells (ISCs) [58,59]. We find that both Ebd and Ewg are required for all major consequences of *Apc1* inactivation in the adult midgut: the hyperactivation of Wingless target genes, excess number of ISCs, and hyperplasia of the epithelium. By contrast, during intestinal homeostasis, Ebd is essential for the Wingless-dependent control of ISC proliferation, whereas Ewg is dispensable. These studies identify transcriptional cofactors that are differentially required for Wnt signaling in physiological conditions versus the pathological states resulting from hyperactivation of the pathway, providing potential selectivity for therapeutic strategies that target Wnt-driven cancers.

## Results

### Apc1 prevents supernumerary progenitor cells and intestinal hyperplasia

**Fig 1:**
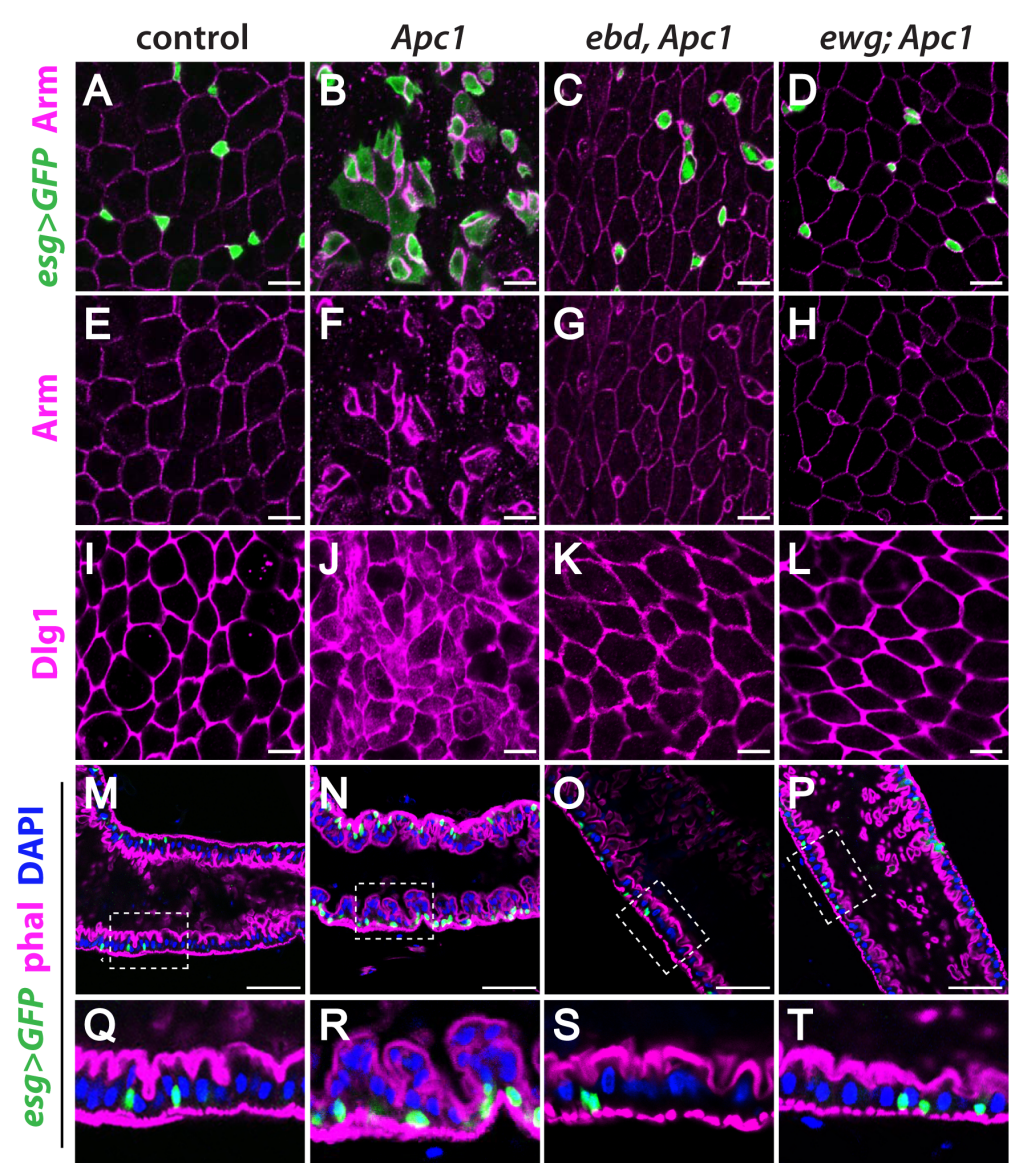
Mutation of *ebd* or *ewg* rescues the intestinal phenotypes resulting from *Apc1* inactivation. (A-D) Increased numbers of *esg>GFP* marked progenitor cells in *Apc1* mutants (green; compare B to A) are suppressed by loss of *ebd* (C) or *ewg* (D). A few chains of small *esg>GFP* positive cells remain in *ebd Apc1* double mutants (C). (E-H) Loss of *Apc1* results in changes in Arm: progenitor cells form chains and clusters with strong Arm staining, whereas ECs have less membrane-associated Arm (magenta, compare F to E). This phenotype is rescued in *ebd Apc1* (G) or *ewg Apc1* (H) double mutants. (I-L) Dlg1 is at the plasma membrane of ECs in control flies (I), but is also cytoplasmic in *Apc1* mutants (J). There is little cytoplasmic Dlg1 in *ebd Apc1* (K) or *ewg Apc1* (L) double mutants. (M-T) The single-layer epithelium of control midguts (M and Q) is replaced by a multi-layered epithelium in *Apc1* mutants (N and R). This phenotype is suppressed by loss of *ebd* (O and S) or *ewg* (P and T). (Q-T) Magnified views of the boxed regions in (M-P). Progenitors are marked with *esg>GFP* (green), the actin-rich cell cortex with phalloidin (phal; magenta), and nuclei with DAPI (blue). Scale bars: (A-L) 10 μm and (M-P) 50 μm. Genotypes: **control:** *esg-Gal4 UAS-GFP/+; Apc1*^*Q8*^*/+* ***Apc1*:** *esg-Gal4 UAS-GFP/+; Apc1*^*Q8*^ ***ebd Apc1*:** *esg-Gal4 UAS-GFP/+; ebd1*^*240*^ *Apc1*^*Q8*^*/Df(3L)9698 ebd2*^*136*^ *Apc1*^*Q8*^ ***ewg Apc1*:** *ewg*^*P1*^*; esg-Gal4 UAS-GFP/+; Apc1*^*Q8*^.

Mammalian genomes encode two *APC* genes: *APC* and *APC2/APCL* with partially redundant roles [60,61]. *APC* is required in the gastrointestinal tract and serves as a gatekeeper that prevents colorectal cancer [11–19,62–66]. The Drosophila genome also encodes two *Apc* genes: *Apc1* and *Apc2* [67–71]. Simultaneous inactivation of both Drosophila *Apc* homologs results in overproliferation of ISCs and hyperplasia of the intestinal epithelium, resembling the mammalian counterpart [72–75]. However, contradictory findings were reported previously regarding the role of Apc1. Two studies indicated that loss of *Apc1* alone results in ISC overproliferation [72,73], whereas another study indicated that Apc1 and Apc2 are fully redundant in this context [75]. To address this controversy, we compared the intestinal epithelium of *Apc1*^*Q8*^ null mutants [67] to controls. In the wild-type intestinal epithelium, ISCs divide asymmetrically to give rise to enteroblasts (EBs) or pre-enteroendocrine (pre-EE) cells, which differentiate into absorptive enterocytes (ECs) or secretory enteroendocrine cells (EEs), respectively [58,59,76–78]. As documented previously [72,73], we found that substantially more progenitor cells (ISCs and EBs) were present in *Apc1* mutant midguts, as revealed by the progenitor cell-specific marker *esg>GFP* (*esg-gal4 UAS-GFP)* (low magnification: S1A and S1B Fig; high magnification: Fig 1A and 1B and quantification: S2A Fig) [58,59]. Furthermore, the number of EBs, as indicated by the expression of *GBE-Su(H)-lacZ* [58], was also increased (S2B and S2C Fig). Thus, our results confirmed that loss of *Apc1* alone results in excess intestinal progenitor cells.

Moreover, we discovered a novel phenotype that results from *Apc1* inactivation: disrupted EC morphology in the midgut epithelium. Levels of membrane-associated Arm in ECs were decreased in *Apc1* mutants (low magnification: S1C and S1D Fig; high magnification: Fig 1E and 1F). Furthermore, Discs large 1 (Dlg1), which is normally restricted to the septate junctions between ECs, was instead diffusely cytoplasmic (Fig 1I and 1J). These findings indicate that the cell-cell junctions and the apico-basal polarity of ECs [59,75,79] were disrupted by loss of *Apc1*. Furthermore, in contrast to the monolayer intestinal epithelium of controls [58,59,74], many ECs were detached from the basement membrane in *Apc1* mutant midguts, forming an aberrantly multi-layered epithelium (low magnification: S1E and S1F Fig, Fig 1M and 1N; high magnification: Fig 1Q and 1R and quantification: S2E and S2F Fig). Together, these findings reveal that loss of *Apc1* alone is sufficient to result in an aberrantly increased number of progenitors, defects in adhesion and epithelial polarity, and disorganization of the intestinal architecture, in a manner analogous to the pathological consequences of *APC* inactivation in mammals.

### Apc1 is essential for both the development and homeostasis of the adult midgut

As the severe intestinal defects present in *Apc1* mutants were readily detected as early as two days of adulthood, we hypothesized that these phenotypes arise during formation of the adult midgut. To test this hypothesis, we examined the *Apc1* mutant gut epithelium shortly after eclosion. Strikingly, compared with the age-matched controls, the midguts of recently eclosed *Apc1* mutants (0–4 hours after eclosion) exhibited supernumerary *esg>GFP* marked progenitor cells (low magnification: S3A and S3C Fig; high magnification: S3B and S3D Fig). The cell-cell adherens junctions, however, remained intact at this time (low magnification: S3A’ and S3C’ Fig; high magnification: S3B’ and D’ Fig). Thus, an excess number of progenitor cells are present prior to eclosion, whereas the disruption of both EC structure and epithelial architecture arise after eclosion. To trace the initial requirement for *Apc1*, we examined midguts earlier in their development. The adult intestine is derived from the larval gut, but undergoes major histolysis and reformation during pupation [80–82]. Therefore, we examined the gut epithelium of *Apc1* mutant third instar wandering larvae, the developmental stage that immediately precedes formation of the adult gut. Notably, supernumerary adult midgut progenitors (AMPs) [80] were not detected in the *Apc1* mutant larval guts (S4A–S4B” Fig) and the epithelial structure was normal (S4C–S4D” Fig). Thus, loss of *Apc1* initiates intestinal defects during formation of the adult gut during pupation, and these defects increase in severity during adulthood.

To further test the temporal and cell-specific requirements for Apc1 in the midgut, we utilized the temperature-sensitive progenitor cell driver (*esg*^*ts*^: *esg-Gal4 tub-Gal80*^*ts*^ *UAS-GFP*) for RNAi-mediated *Apc1* knockdown in ISCs and EBs either during formation of the adult gut or during adult gut homeostasis. Of note, dramatic increases in the progenitor cell number were observed in both contexts (adult gut formation: S5A–S5D’ Fig, adult gut homeostasis: S5E–S5H’ Fig; quantification: S5I Fig). Furthermore, we used an inducible “escargot flip out” system (*esg*^*ts*^*>F/O*: *esg-Gal4 tub-Gal80*^*ts*^ *UAS-GFP; UAS-flp Act>CD2>Gal4* [83]) to mark the progenitor cells and their progeny in which *Apc1* was knocked down during adulthood. Compared with controls, increased phospho-histone H3 (pH3) positive cells and stem/progenitor cell lineages were observed within *Apc1* RNAi “escargot flip out” posterior midguts (S6A–S6B” Fig; quantification: S6C and S6D Fig), providing further evidence that adult-specific *Apc1* knockdown resulted in increased ISC proliferation. Together, these results indicate that Apc1 is required in stem/progenitor cells during both adult gut development and homeostasis, supporting our observations in *Apc1* null mutants (Fig 1 and S1–S4 Figs) and previous reports [72,73].

### Apc1 prevents the constitutive activation of Wingless target genes in the adult midgut

**Fig 2:**
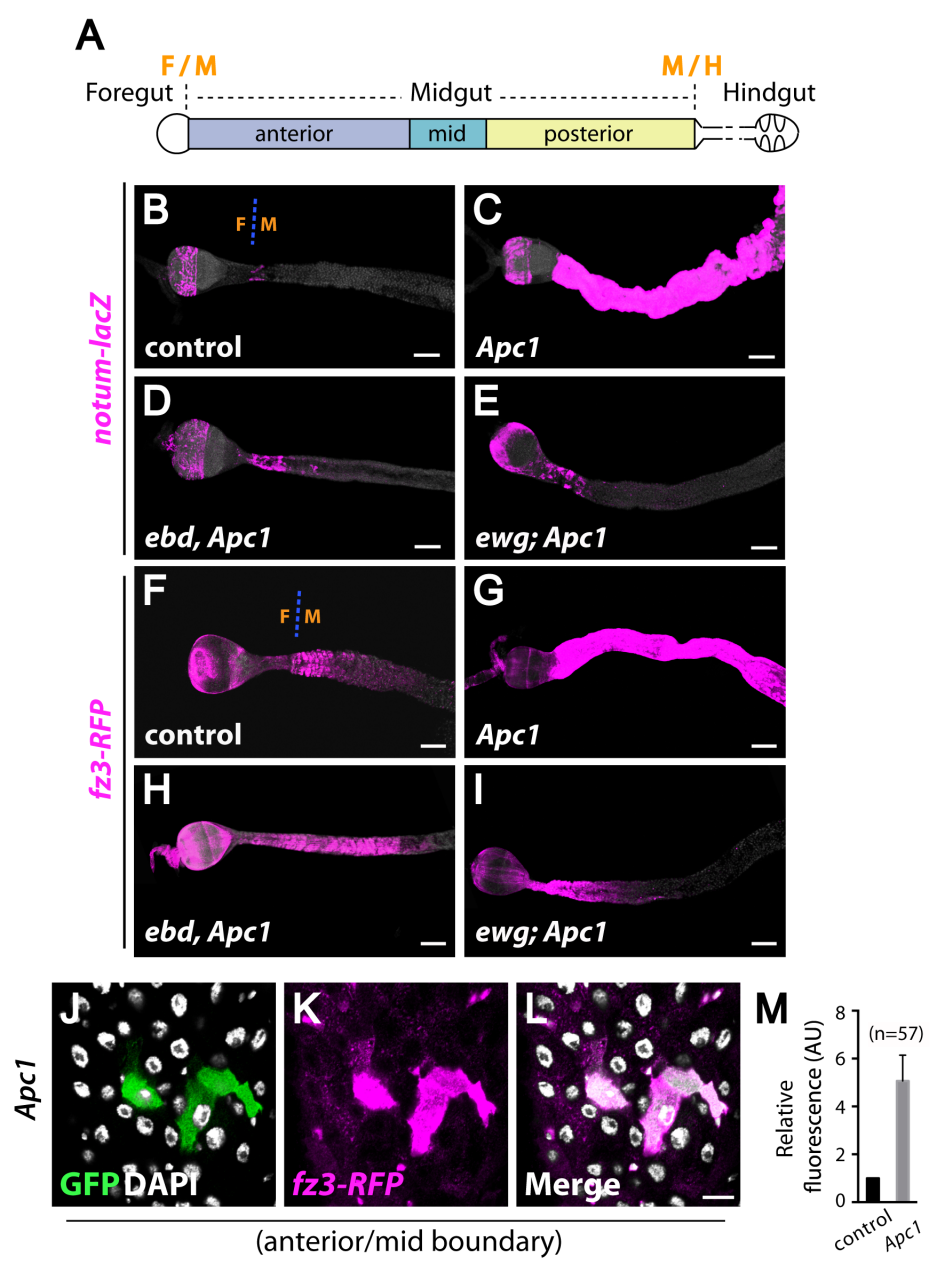
Increased expression of Wingless target genes in *Apc1* mutant midguts requires Ebd and Ewg. (A) The Drosophila intestine is partitioned into distinct compartments: the foregut, midgut and hindgut. The midgut can be further subdivided into the anterior, mid, and posterior midgut. (B-I) Expansion of *notum-lacZ* (magenta; B and C) and *fz3-RFP* (magenta; F and G) expression upon loss of *Apc1* is suppressed by inactivation of *ebd* (D and H) or *ewg* (E and I). The foregut (F)-midgut (M) boundary is marked in (B) and (F). (J-M) The expression of *fz3-RFP* (magenta) in *Apc1* mutant clones (marked with GFP, green) at compartment boundaries is increased by approximately fivefold compared to its expression in neighboring control tissue (GFP negative). Nuclei are marked with DAPI (white). Scale bars: (B-I) 100 μm and (J-L) 10 μm. Genotypes: (B-E) **control:** *notum-lacZ/+; Apc1*^*Q8*^*/+* ***Apc1*:** *notum-lacZ/+; Apc1*^*Q8*^ ***ebd Apc1*:** *notum-lacZ/+; ebd1*^*240*^ *Apc1*^*Q8*^*/Df(3L)9698 ebd2*^*136*^ *Apc1*^*Q8*^ ***ewg Apc1***: *ewg*^*P1*^*; notum-lacZ/+; Apc1*^*Q8*^ (F-I) **control:** *fz3-RFP/+; Apc1*^*Q8*^*/+* ***Apc1*:** *fz3-RFP/+; Apc1*^*Q8*^ ***ebd Apc1*:** *fz3-RFP/+; ebd1*^*240*^ *Apc1*^*Q8*^*/Df(3L)9698 ebd2*^*136*^ *Apc1*^*Q8*^ ***ewg Apc1***: *ewg*^*P1*^*; fz3-RFP/+; Apc1*^*Q8*^ (J-M) ***Apc1*:** *y w hs-flp UAS-CD8*::*GFP/+; fz3-RFP/+; tub-Gal4 FRT82B tub-Gal80/FRT82B Apc1*^*Q8*^.

Both the initiation and sustained growth of human colon cancers harboring *APC* mutations rely on the aberrant activation of Wnt target genes [10,15,16,18,20–31]. To examine whether loss of Drosophila *Apc1* also induces the aberrant activation of Wingless target genes in the midgut, we tested three transcriptional reporters for direct target genes of Wingless signaling: *frizzled3 (fz3)*, *notum*, and *naked cuticle (nkd)* [84–88]. The Drosophila midgut, like its mammalian counterpart, is subdivided into compartments with distinct histology, gene expression, and physiological functions (Fig 2A) [89–91]. The activation of Wingless signaling is graded along the length of each intestinal compartment; Wingless target genes are activated at high levels at intestinal compartment boundaries and at lower levels within compartments as a function of distance from the boundary [89,92]. We found that inactivation of *Apc1* resulted in strong ectopic expression of *notum-lacZ* [93,94] (low magnification view: S7A and S7B Fig; high magnification view: Fig 2B and 2C), *fz3-RFP* [95] (low magnification view: S7E and S7F Fig; high magnification view: Fig 2F and 2G) and *nkd(UpE2)-lacZ* [96–98] (low magnification view: S8A and S8B Fig; high magnification view: S8D and S8E Fig), both at compartment boundaries and within compartments of *Apc1* null mutant midguts. To quantify this aberrant increase in expression, we examined *fz3-RFP* in marked clones of *Apc1* null mutant cells generated with the MARCM (Mosaic Analysis of a Repressible Cell Marker) technique [99]. Even at compartment boundaries, where Wingless pathway activity is normally at its peak, a further fivefold increase in *fz3-RFP* levels was present in *Apc1* null mutant clones as compared to the surrounding control tissue (Fig 2J–2L; quantification: Fig 2M). These findings indicate that Apc1 is required to prevent the constitutive activation of Wingless target genes at both compartment boundaries and within compartments of the midgut.

### *Apc1* inactivation results in extensive deregulation of gene expression

**Fig 3:**
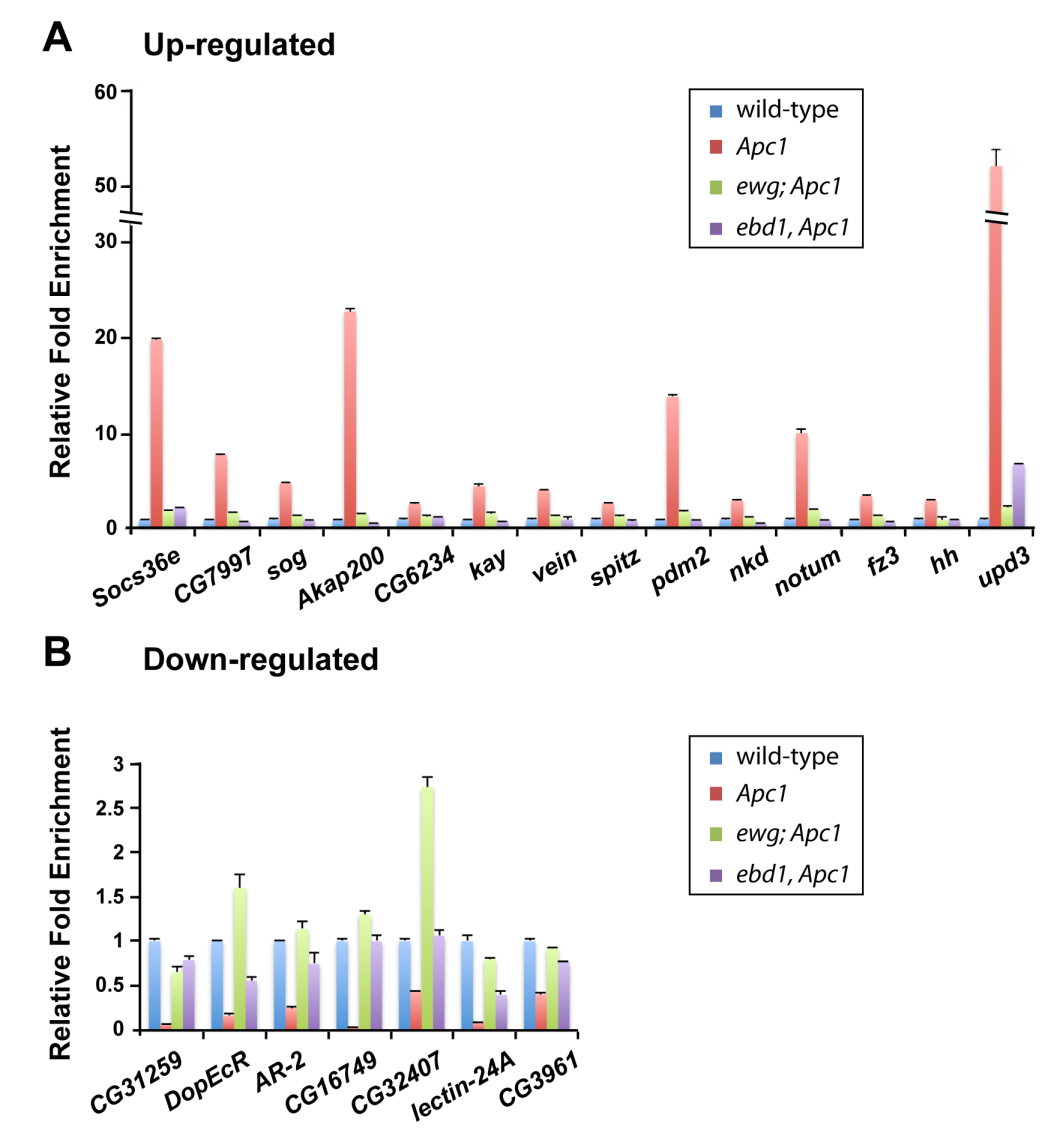
Deregulation of gene expression in the midgut upon loss of *Apc1* requires Ebd1 and Ewg. Quantitative RT-PCR of genes up- (A) or down-regulated (B) following loss of *Apc1*. Deregulation of both sets of genes is rescued in *ebd1 Apc1* and *ewg Apc1* double mutants. Genotypes: **control:** *Apc1*^*Q8*^*/+* ***Apc1*:** *Apc1*^*Q8*^ ***ebd1 Apc1*:** *ebd1*^*240*^ *Apc1*^*Q8*^ ***ewg Apc1*:** *ewg*^*P1*^*; Apc1*^*Q8*^.

We sought to determine the extent to which *Apc1* loss deregulates gene expression. Using Affymetrix microarrays, we found that the expression of approximately 1000 genes, which can be grouped in broad categories, was either up- or down-regulated by more than twofold in *Apc1* mutant midguts as compared to wild-type controls (GEO database: GSE99071; S9 Fig). The changes in expression of selected up- or down-regulated genes were validated by real-time quantitative PCR (Fig 3 and S10 Fig). Consistent with the Wingless target gene reporter analysis described above (Fig 2 and S7 and S8 Figs), *fz3*, *notum* and *nkd* transcription were activated significantly in *Apc1* mutants (Fig 3A and S10A Fig). Furthermore, a previous study identified Janus kinase/signal transducer and activator of transcription (Jak/Stat) and Epidermal growth factor receptor (Egfr) signaling as key mediators of ISC hyperproliferation in *Apc1* mutants [73]. Indeed, the expression of both *unpaired 3* (*upd3)*, a ligand of the Jak/Stat pathway, and *Socs36e*, a downstream target gene of this pathway, were induced upon loss of *Apc1* (Fig 3A and S10A Fig). Furthermore, the expression of *vein* (*vn*) and *spitz* (*spi)*, two ligands of the Egfr pathway, was also increased (Fig 3A and S10A Fig). Together, these results suggest that loss of *Apc1* results in an extensive deregulation of gene expression.

### Both Ebd and Ewg are required for the excess progenitor cells and intestinal epithelial hyperplasia that result from *Apc1* inactivation

In a forward genetic screen in the Drosophila retina, we previously identified both Ebd1 and Ewg [53,54] as novel suppressors of *Apc1*. Furthermore, we found that these two proteins function as transcriptional cofactors that physically interact both with each other and with Arm/Tcf to promote the context-specific activation of Wingless signaling during pupal muscle development [53,54]. However, the limited genetic tools available to analyze Wnt signaling in pupal muscle restricted our ability to identify the roles of Ebd and Ewg in physiological versus pathological Wnt signaling. Herein, we sought to overcome this obstacle by utilizing powerful *in vivo* assays in the Drosophila intestine.

First, we sought to determine whether Ebd and/or Ewg are required for the phenotypic consequences of *Apc1* inactivation in the intestine by examining *ebd Apc1* and *ewg Apc1* double mutants. Strikingly, the three major defects in the midguts of *Apc1* mutants were largely suppressed upon inactivation of either *ebd* or *ewg*. First, the numbers of progenitor cells in *ebd Apc1* or *ewg Apc1* double mutants were similar to those in controls (Fig 1A–1H and S2B–S2D Fig; quantification: S2A Fig). Second, the levels of membrane-associated Arm and the subcellular localization of Dlg1 in ECs were indistinguishable from controls (Fig 1E–1L). Third, in *ebd Apc1* and *ewg Apc1* double mutants, the midguts reverted to a monolayer epithelium (low magnification: Fig 1M–1P; high magnification: Fig 1Q–1T and quantification: S2E and S2F Fig). Thus, both Ebd and Ewg are required for the excess progenitor cells and epithelial hyperplasia resulting from *Apc1* inactivation.

### Both Ebd and Ewg are required for the aberrant activation of Wingless target genes in the midgut resulting from *Apc1* inactivation

To determine whether Ebd or Ewg are required for the aberrantly high expression of Wingless target genes that results from *Apc1* inactivation, we examined the expression of *notum-lacZ*, *fz3-RFP* and *nkd(UpE2)-lacZ* in *ebd Apc1* and *ewg Apc1* double mutants. Strikingly, upon loss of either *ebd* or *ewg*, the hyperactivation of all three Wingless pathway reporters in *Apc1* mutant midguts was reduced nearly to control levels (Fig 2B–2E, Fig 2F–2I, S7 and S8 Figs). Thus, not only Arm/β-catenin and TCF, but also Ebd and Ewg are required for the aberrantly increased activation of Wingless target genes in *Apc1* mutant midguts.

### Both Ebd and Ewg are required for the extensive deregulation of transcription resulting from *Apc1* inactivation in the midgut

We sought to test whether Ebd and Ewg are required for the hyperactivation of only a subset of direct Wingless target genes or also have broader effects in the extensive deregulation of gene expression that occurs in *Apc1* mutants. Therefore, we analyzed the expression of genes that are selectively up- or down-regulated genes by *Apc1* inactivation in either *ebd Apc1* or *ewg Apc1* double mutants by real-time quantitative PCR (Fig 3 and S10 Fig). Of note, the transcriptional deregulation resulting from loss of *Apc1* was rescued in *ebd1 Apc1* or *ewg Apc1* double mutants for all genes analyzed (Fig 3 and S10 Fig). These findings provide further evidence that the aberrant transcriptional response in *Apc1* mutant midguts requires both Ebd and Ewg.

Ewg is a known sequence-specific DNA binding protein [54–57]. Therefore, we sought to determine whether consensus Ewg DNA binding sites are present in the enhancers of genes deregulated by *Apc1* loss. As the Wingless target gene reporters *notum-lacZ*, *nkd(UpE2)-lacZ*, and *fz3-RFP* are each hyperactivated in an Ewg-dependent manner following *Apc1* loss, and the enhancers within these reporters are well-characterized, we searched for potential Ewg and TCF binding sites in these enhancers. The transcriptional enhancers that drive expression of both the *notum-lacZ* and *nkd(UpE2)-lacZ* reporters, which are 2.2 kb [93,94] and 0.6 kb [97], respectively, are directly bound and regulated by TCF through distinct pairs of core consensus sites (SSTTTGWWSWW) and Helper sites (GCCGCCR) [5,96–98,100] (S11A–S11C Fig). We identified similar TCF core consensus binding sites and Helper sites in the 2.3 kb enhancer of the *fz3-RFP* transgene [95] (S11D Fig). In addition, we found that the *fz3* enhancer contains an Ewg consensus binding site (GCGCABGY) [54–57] (S11A and S11D Fig), and that this site is conserved among sequenced Drosophila species (S12 Fig) [101]. In contrast, neither the *notum* nor the *nkd* enhancer contains an Ewg consensus binding site (S11A–S11C Fig). Therefore, these findings suggest that the hyperactivation of at least some Wingless target genes in *Apc1* mutants may not require direct binding of Ewg to DNA, or alternatively, that Ewg may bind non-consensus DNA sites upon *Apc1* inactivation.

### Ebd and Ewg are required in progenitor cells to mediate the defects resulting from *Apc1* loss

**Fig 4:**
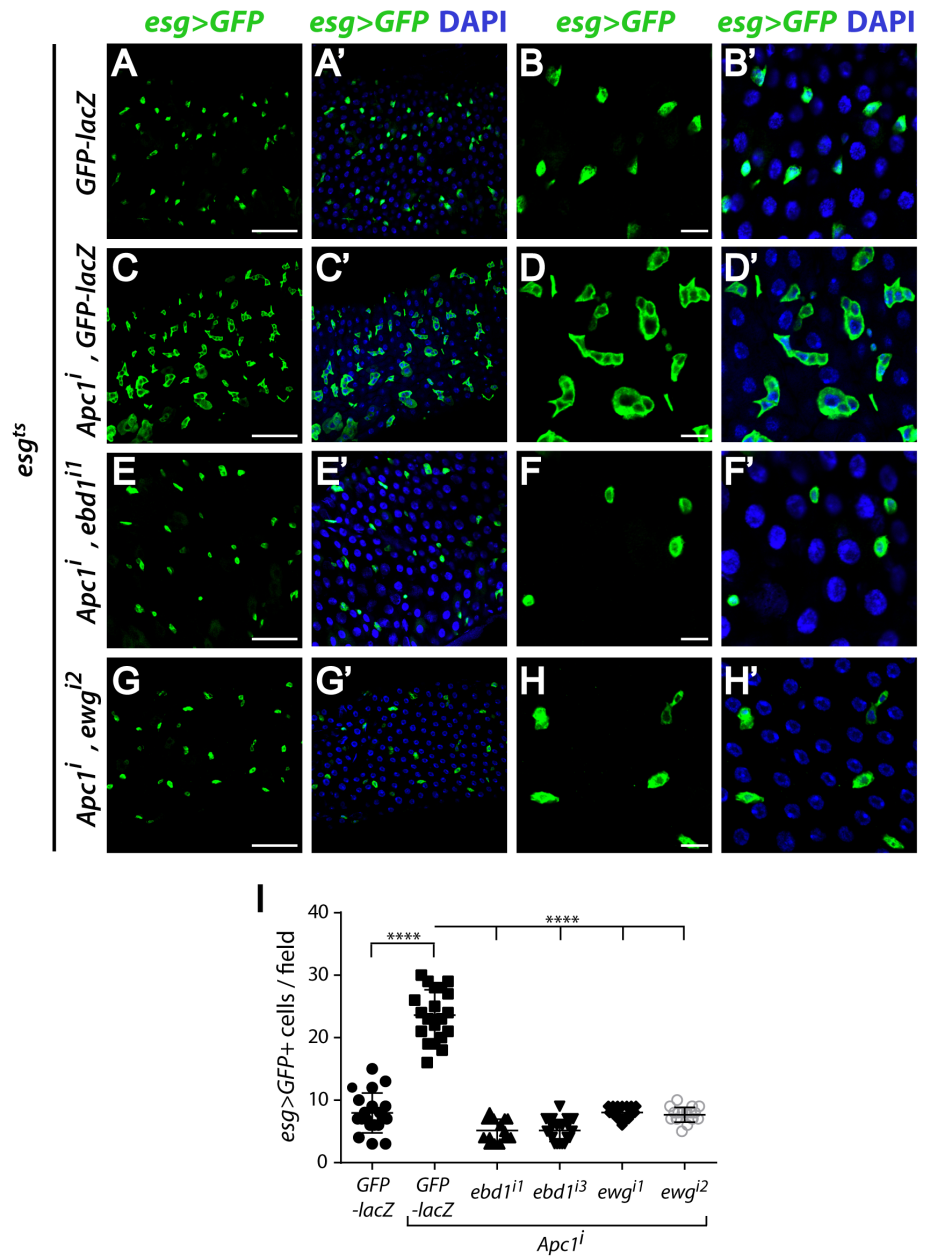
Ebd and Ewg are required in progenitors to mediate *Apc1* mutant intestinal defects. (A-H’) The excess progenitor cells (marked by *esg>GFP*, green) that result from reducing *Apc1* activity (A-D’) are suppressed upon concomitant knockdown of either *ebd* (E-F’) or *ewg* (G-H’) in progenitors. Crosses were shifted from 18°C to 29°C during the second instar larval stage and progeny of desired genotype were examined 2–3 days post-eclosion. Low magnification view (scale bars: 50 μm): A-A’, C-C’, E-E’ and G-G’; high magnification view (scale bar: 10 μm): B-B’, D-D’, F-F’ and H-H’. Nuclei are labeled by DAPI (blue). (I) Concomitant RNAi-mediated knockdown in progenitor cells of *Apc1* and either *ebd1* or *ewg* rescues the *Apc1* RNAi-mediated supernumerary progenitor defects, as measured by *esg>GFP* staining. **** P<0.0001 (t-test). Genotypes: ***GFP-lacZ*:** *esg-Gal4 tubGal80*^*ts*^ *UAS-GFP/+; UAS-GFP-lacZ* ***Apc1***^***i***^**, *GFP-lacZ*:** *UAS-Dicer2/+; esg-Gal4 tubGal80*^*ts*^ *UAS-GFP/+; UAS-Apc1 RNAi#1/UAS-GFP-lacZ* ***Apc1***^***i***^**, *ebd1***^***i1***^: *UAS-Dicer2/+; esg-Gal4 tubGal80*^*ts*^ *UAS-GFP/+; UAS-Apc1 RNAi#1/UAS-ebd1 RNAi#1* ***Apc1***^***i***^**, *ebd1***^***i3***^: *UAS-Dicer2/+; esg-Gal4 tubGal80*^*ts*^ *UAS-GFP/+; UAS-Apc1 RNAi#1/UAS-ebd1 RNAi#3* ***Apc1***^***i***^**, *ewg***^***i2***^: *UAS-Dicer2/+; esg-Gal4 tubGal80*^*ts*^ *UAS-GFP/+; UAS-Apc1 RNAi#1/UAS-ewg RNAi#2* ***Apc1***^***i***^**, *ewg***^***i1***^: *UAS-Dicer2/+; esg-Gal4 tubGal80*^*ts*^ *UAS-GFP/+; UAS-Apc1 RNAi#1/UAS-ewg RNAi#1*.

RNAi-mediated knockdown of *Apc1* specifically in progenitor cells phenocopies the supernumerary progenitors observed in *Apc1* null mutants (S5 Fig). Therefore, we hypothesized that Ebd and Ewg act in progenitor cells to mediate the phenotypic consequences of *Apc1* loss. To test this hypothesis, we used RNAi-mediated knockdown to concomitantly reduce both *Apc1* and *ebd* or *ewg* in progenitors. The aberrant increase in progenitor cell number resulting from *Apc1* knockdown was largely suppressed upon simultaneous knockdown of either *ebd* or *ewg* (Fig 4A–4H’; quantification: Fig 4I). Based on these findings, we conclude that Ebd and Ewg are required in progenitors to mediate the gut defects resulting from *Apc1* loss.

### Ebd1 promotes Wingless target gene activation in the adult midgut under physiological conditions, whereas Ewg is dispensable

**Fig 5:**
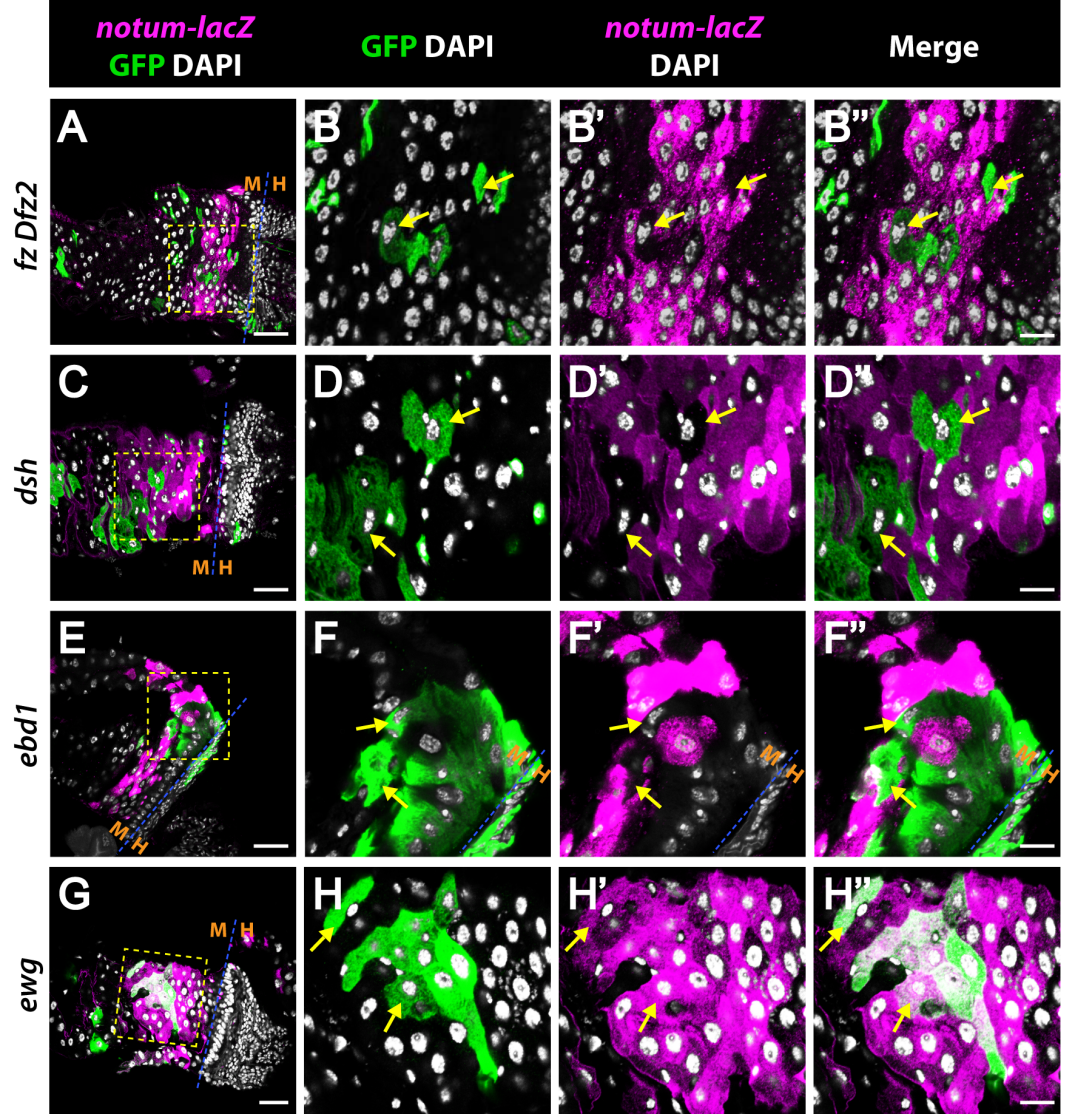
Ebd1 is required for physiological Wingless signal transduction, whereas Ewg is dispensable. Expression of *notum-lacZ* (magenta), a Wingless target gene reporter, is diminished in mutant clones of *fz Dfz2* (A-B”), *dsh* (C-D”) and *ebd1* (E-F”) (arrows), but is retained in *ewg* mutant clones (G-H”), suggesting Ebd1 is required for Wingless-dependent expression of *notum* during homeostasis. Ewg, in contrast, is dispensable for this process. Clones are marked with GFP (green) and the midgut-hindgut (M-H) boundary is indicated (A, C, E-F”‘ and G). Magnified views of the boxed regions in (A, C, E and G) are shown in (B-B”, D-D”, F-F” and H-H”), respectively. Scale bars: (A, C and E) 30 μm, (G) 25 μm and (B-B”, D-D”, F-F” and H-H”) 10 μm. Genotypes: ***fz Dfz2***: *y w hs-flp/+; tub-Gal4 UAS-GFP/notum-lacZ; FRT2A tub-Gal80/FRT2A fz^H51^ Dfz2^C1^* ***dsh*:** *hs-flp tub-Gal80 FRT19A/dsh*^*3*^ *FRT19A; notum-lacZ/+; tub-Gal4 UAS-mCD8*::*GFP/+* ***ebd1*:** *y w hs-flp/+; tub-Gal4 UAS-GFP/notum-lacZ; FRT2A tub-Gal80/ebd1*^*5*^ *FRT2A* ***ewg*:** *hs-flp tub-Gal80 FRT19A/ewg*^*2*^ *FRT19A; notum-lacZ/+; tub-Gal4 UAS-mCD8*::*GFP/+*.

Our findings indicate that both Ebd1 and Ewg are required for the aberrantly increased expression of the Wingless target genes resulting from *Apc1* loss in the adult midgut (Fig 2 and Fig 3; S7, S8 and S10 Figs). To determine whether Ebd1 and Ewg also promote Wingless target gene expression in the adult midgut under physiological conditions, we analyzed the expression of the Wingless target gene reporter *notum-lacZ*. Under basal conditions, *notum-lacZ* expression peaks at both the foregut/midgut (Fig 2B) and the midgut/hindgut boundaries (Fig 5). This expression of *notum-lacZ* is completely dependent on Wingless pathway activation, as revealed by its loss in *fz Dfz2* double null mutant [102] or *dsh* null mutant clones [103] (Fig 5A–5D”). We found that in many, but not all *ebd1* null mutant clones, *notum-lacZ* expression was eliminated (Fig 5E–5F”), providing evidence that Ebd1 promotes the activation of Wingless target genes not only in hyperactivated states, but also during homeostasis in the adult midgut. In contrast, *ewg* null mutant clones resulted in no detectable reduction in the expression of *notum-lacZ* (Fig 5G–5H”). In addition, *ewg* null mutant clones also did not affect the expression of the other two Wingless target gene reporters, *fz3-RFP* (S13A–S13B” Fig) or *nkd-lacZ* (S13C–S13D” Fig), suggesting that Ewg is dispensable for Wingless target gene activation in the adult midgut under physiological conditions. Thus, these findings indicate although both Ebd and Ewg are essential for the hyperactivation of Wingless signaling upon *Apc1* inactivation, only Ebd is required for Wnt pathway activation during intestinal homeostasis, whereas Ewg is dispensable.

### Ebd is required for adult intestinal homeostasis, whereas Ewg is dispensable

**Fig 6:**
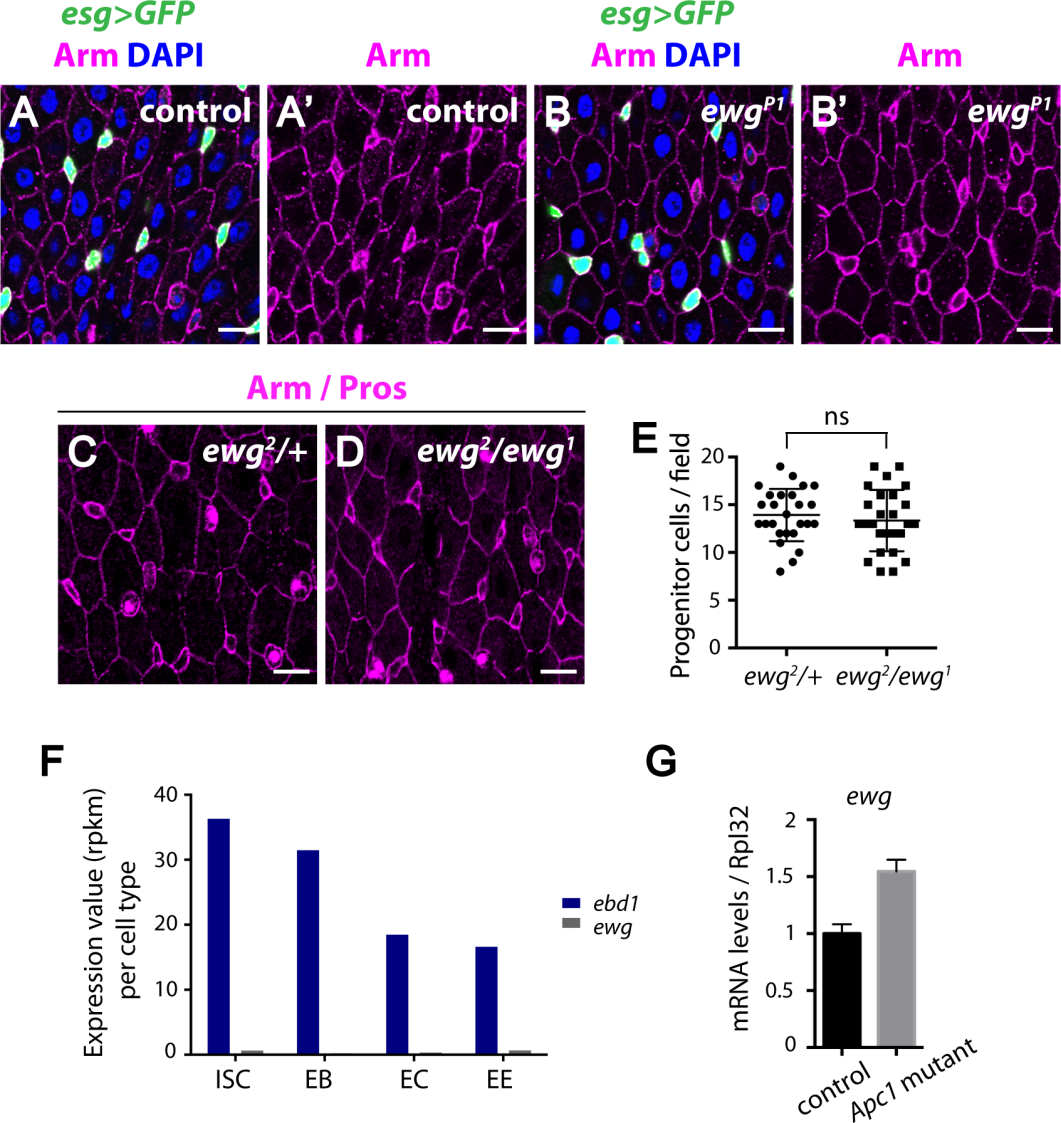
Ewg is not required for intestinal homeostasis in physiological conditions. (A-B’) No overt change in the number of progenitor cells (marked with *esg>GFP*, green A and B) is detected in *ewg*^*P1*^ mutants compared to controls, suggesting that Ewg is not required for homeostasis of intestinal tissues under physiological conditions. In addition, *ewg*^*P1*^ mutants exhibit a normal, well-organized epithelial structure (marked with Arm, magenta A’ and B’). (C-D) Similarly, no obvious change in the number of progenitor cells is detected in *ewg*^*2*^*/ewg*^*1*^ mutants compared to controls. Progenitor cells are identified as small cells with strong Arm staining and the absence of Prospero staining. (E) Quantification of progenitor cell numbers in a defined field in the posterior midguts of age matched *ewg*^*2*^*/+* and *ewg*^*2*^*/ewg*^*1*^ flies. ns: not significant (t-test). (F) Cell type specific RNA-seq of Drosophila intestines (performed by Dr. Buchon’s lab; FlyGut-seq [104]) revealed that under homeostatic conditions, *ebd1* is expressed in all gut cell types, whereas *ewg* is expressed at very low levels in the gut. RPKM: Reads Per Kilobase of transcript per Million. (G) Quantification of Ewg expression levels in control and *Apc1* mutant guts. Scale bars: (A-D) 10 μm. Genotypes: (A-B’) **control:** *esg-Gal4 UAS-GFP/+* ***ewg***^***P1***^: *ewg*^*P1*^*; esg-Gal4 UAS-GFP/+* (G) **control:** *Apc1*^*Q8*^*/+* ***Apc1 mutant*:** *Apc1*^*Q8*^.

Wingless pathway activation is crucial for the maintenance of adult midgut homeostasis [89,92]. We thus sought to determine whether Ebd and Ewg are required for this process. To test whether Ewg has a role during adult midgut homeostasis, we first analyzed *ewg*^*P1*^, a hypomorphic allele containing an *ewg* missense mutation ([54]; note that complete inactivation of *ewg* results in embryonic lethality). In comparison with controls, *ewg*^*P1*^ midguts contained comparable numbers of progenitor cells (marked by *esg>GFP*) and displayed normal epithelial architecture (Fig 6A–6B’). Thus, although this allele revealed that Ewg is crucial for the hyperactivated Wingless signaling and intestinal hyperplasia that results from *Apc1* inactivation in the adult midgut (Figs 1–3 and S1, S2, S7, S8 and S10 Figs), it exhibits no detectable defects under physiological conditions. To further reduce the level of *ewg* activity, we examined the midguts of flies transheterozygous for the null allele *ewg*^*2*^ and the hypomorphic allele *ewg*^*1*^, which is the most severe viable combination of *ewg* alleles (all mutant flies exhibit “erect wing” defects) [54,55]. Notably, no excess progenitors were observed in *ewg*^*2*^/*ewg*^*1*^ transheterozygotes (Fig 6C and 6D; quantification: Fig 6E). These results suggested that consistent with our observation that Ewg is dispensable for physiological Wingless target gene activation, Ewg does not have a role in Wingless-dependent adult intestinal homeostasis. Gut cell type specific RNA-seq under homeostatic condition revealed previously [104] that expression of Ewg is very low during intestinal homeostasis, while Ebd1 is expressed in all gut cell types (Fig 6F). Thus, the possibility arose that loss of *Apc1* induces overexpression of *ewg* and this might explain why Ewg is essential for hyperactivated Wingless signaling but dispensable for physiological signaling. However, RT-qPCR of *ewg* expression revealed a less than 2-fold increase in *Apc1* mutants (Fig 6G), suggesting that an increase in Ewg levels is unlikely the mechanism by which Ewg mediates the consequences of *Apc1* loss in the adult midgut.

We next analyzed whether Ebd is required during adult intestinal homeostasis. In Drosophila, two Ebd proteins, Ebd1 and Ebd2, possess partially redundant functions [53]. To elucidate the function of Ebd1 in the midgut, we compared the intestinal epithelium of control (*ebd1/+*) to *ebd1*^*240*^ null mutants [53]. We found that the number of progenitor cells (marked by *esg>GFP* or combination of Arm/Prospero staining) was significantly increased in *ebd1* mutants (S14A–S14F Fig; quantification: S14M). In addition, in contrast to the control midguts, very few of which (8%) displayed chains of progenitors and none of which exhibited clusters of progenitor cells, the majority of *ebd1* mutant midguts (60%) contained chains of progenitor cells and 15% exhibited clusters (S14A–S14F Fig, quantification: S14N Fig). To further determine whether this requirement for Ebd1 in the regulation of ISC proliferation occurs during adulthood, we generated marked *ebd1* null mutant clones in adults. We found that *ebd1* mutant clones were markedly larger than control clones: 44% of *ebd1* mutant clones contained more than 4 cells, as compared to 14% of the control clones (S15 Fig). Together, these findings indicate that in contrast to Ewg, Ebd1 is required for intestinal homeostasis during adulthood, resembling other Wingless pathway components [89,92].

**Fig 7:**
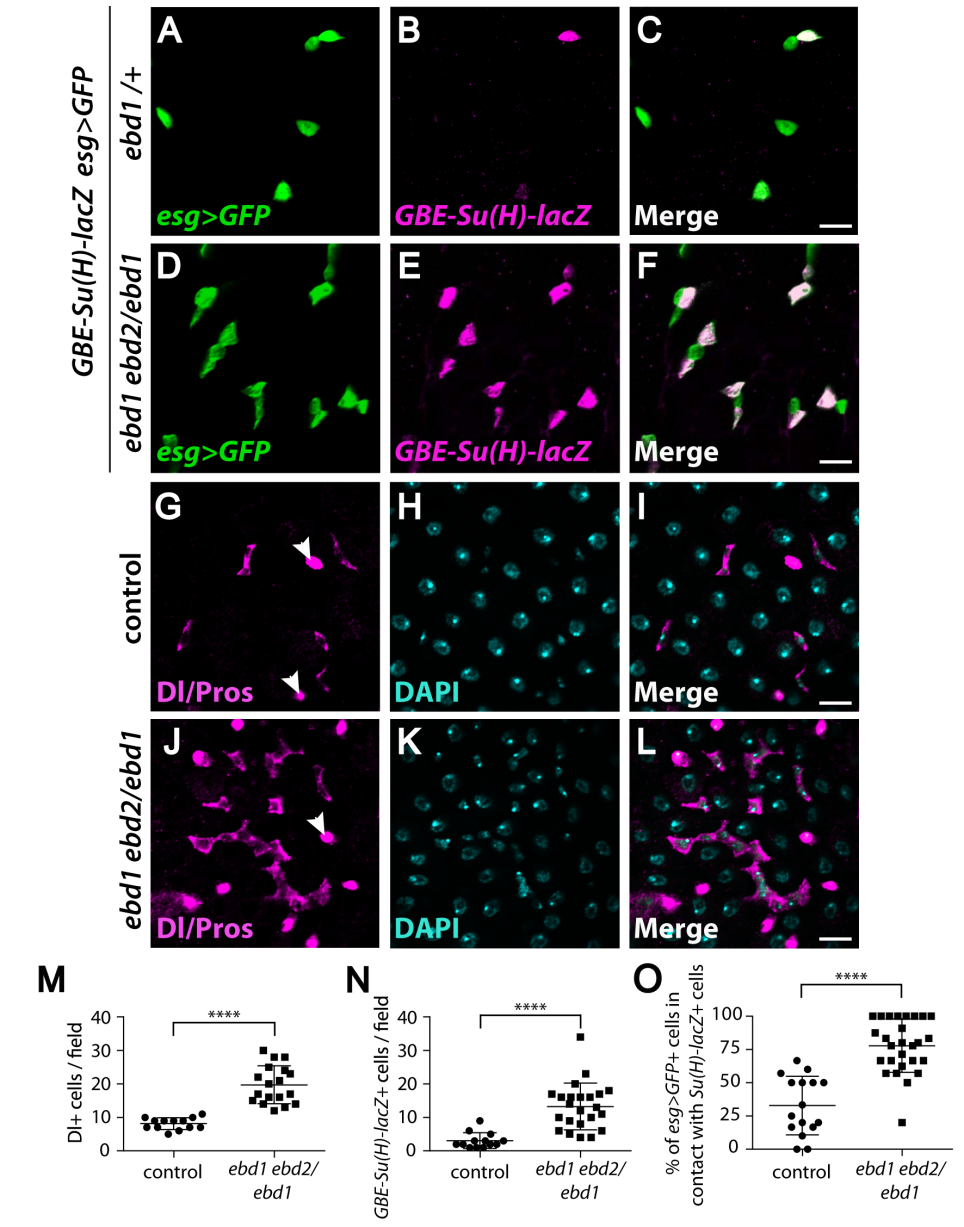
Ebd inhibits the proliferation of intestinal stem cells. (A-F) Loss of *ebd* results in increased numbers of progenitor cells (marked with *esg>GFP*, green), including EBs (marked with *GBE-Su(H)-lacZ*, magenta) (compare D-F with A-C). Nearly all *esg>GFP* positive cells are associated with at least one *GBE-Su(H)-lacZ* positive cell in *ebd1 ebd2/ebd1* mutants (D-F), suggesting that the mutant ISCs have a higher proliferation rate. (G-L) Loss of *ebd* leads to increased proliferation of ISCs, marked by Dl staining (magenta, compare J-L with G-I). EEs are stained with Prospero (Pros, magenta, arrowheads) and nuclei with DAPI (turquoise). (M and N) *ebd1 ebd2/ebd1* mutants have higher numbers of Dl positive ISCs (M) and *GBE-Su(H)-lacZ* positive EBs (N) compared to control. (M) **** P<0.0001 (t-test), (N) **** P<0.0001 (t-test). (O) An increased number of ISCs are associated with EBs in *ebd1 ebd2/ebd1* mutants compared to controls. **** P<0.0001 (Mann-Whitney test). Scale bars: (A-L) 10 μm. Genotypes: (A-F) ***ebd1/+*:** *GBE-Su(H)-lacZ/+; esg-Gal4 UAS-GFP/+; ebd1*^*240*^*/+* ***ebd1 ebd2/ebd1*:** *GBE-Su(H)-lacZ/+; esg-Gal4 UAS-GFP/+;* e*bd1*^*5*^ e*bd2*^*136*^*/* e*bd1*^*240*^ (G-L) **control:** Canton S ***ebd1 ebd2/ebd1*:** e*bd1*^*5*^ e*bd2*^*136*^*/* e*bd1*^*240*^.

We further sought to determine whether the combined inactivation of *ebd1* and *ebd2* would result in a more severe phenotype than inactivation of *ebd1* singly. Staining for *esg>GFP* revealed that by comparison with *ebd1* single mutants, a larger proportion of *ebd1ebd2/ebd1* mutant midguts displayed clusters of progenitor cells (42%), and this proportion increased further in midguts homozygous mutant for both *ebd1* and *ebd2* (58%) (S14G–S14L Fig; quantification: S14N Fig), indicating that *ebd2* inactivation exacerbated the severity of *ebd1* null mutant phenotype. To further differentiate the subtypes of progenitor cells that are deregulated by loss of the Ebd1 and Ebd2 proteins, we examined the ISC-specific marker Delta (Dl) [105] and the EB-specific marker *GBE-Su(H)-lacZ* in *ebd1 ebd2/ebd1* mutants, and detected a significant increase in the number of both cell types as compared to controls (Fig 7A–7L; quantification: Fig 7M and 7N). Furthermore, 33% of ISCs (*esg*+, *GBE-Su(H*)-) were associated with an EB (*esg*+, *GBE-Su(H*)+) in controls, but this number increased to 78% in *ebd1 ebd2/ebd1* mutants (Fig 7O). Together, both Ebd1 and Ebd2 are required for homeostasis of the Drosophila midgut as their inactivation leads to an aberrant increase in both ISCs and EBs.

### Ebd1 non-autonomously prevents aberrant ISC overproliferation

To determine the cell types in which Ebd1 is expressed in the midgut, we immunostained intestines with Ebd1 antibody [53] (S16A–S16A” Fig). Using *ebd1* null mutant clones to test the specificity of the Ebd1 antibody, we found that Ebd1 is expressed in enterocytes (S16B–S16C”‘ Fig). We also detected Ebd1 in progenitors and EEs, but based on mutant clonal analysis, it was not clear that this staining was above background (S16B–S16C”‘ Fig). Therefore we conclude that there is strong Ebd1 expression in ECs, and any Ebd1 expression in progenitors cells or EEs is lower than the detection limit of the Ebd1 antibody. We also tested *ebd1-Gal4* lines [53] to drive reporter expression, which revealed that Ebd1 is strongly expressed in ECs, but also detectable at lower levels in progenitors and EEs (S17 Fig).

**Fig 8:**
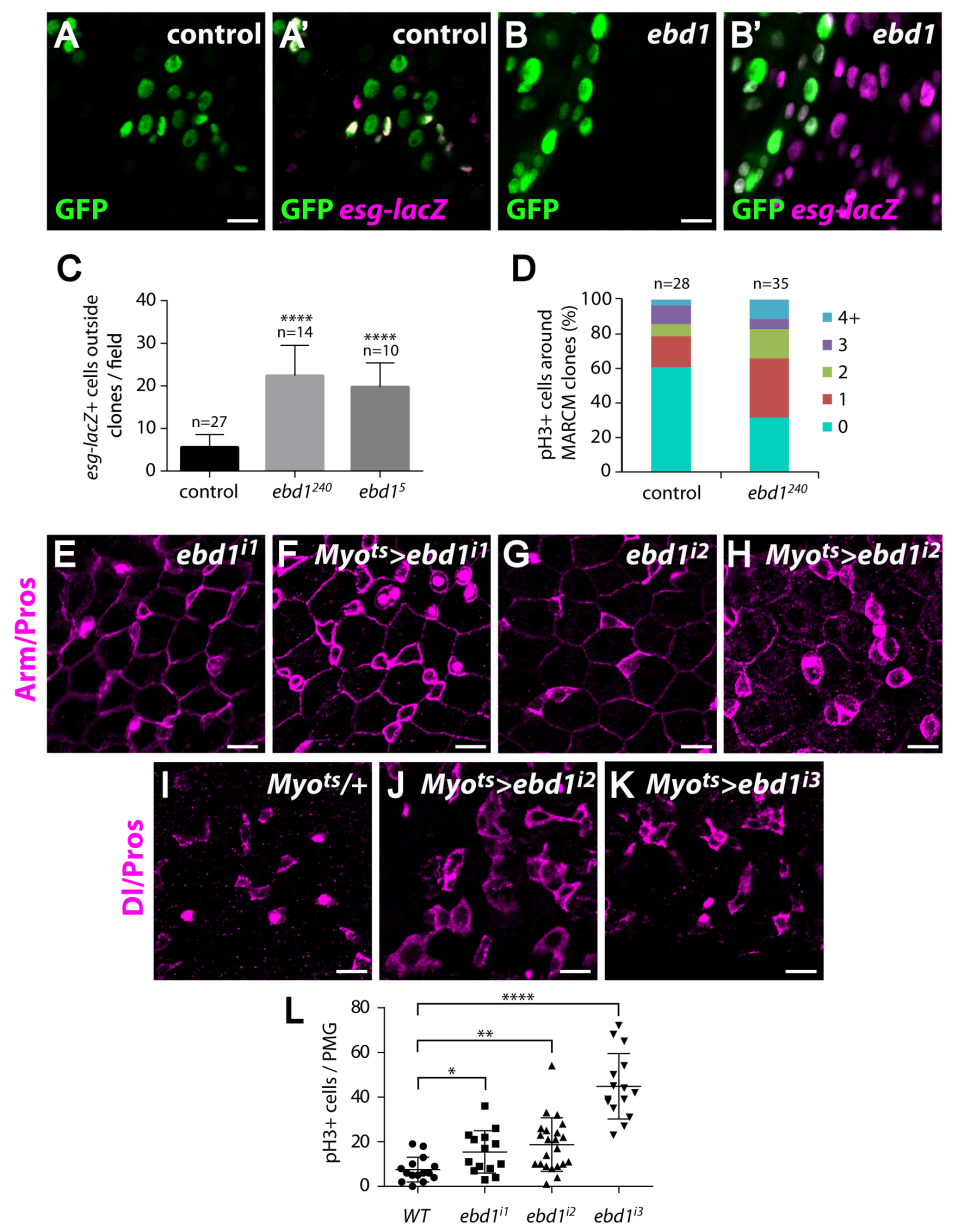
Ebd1 regulates stem cell proliferation non-autonomously. (A-B’) There are more progenitor cells, marked with *esg-lacZ* (magenta), near *ebd1*^*240*^ mutant clones (GFP labeled clones in green, B and B’), than near wild-type (control) clones (green, A and A’). (C) Quantification of the number of *esg-lacZ* positive cells around control clones and around clones of two independent *ebd1* null alleles, e*bd1*^*240*^ and e*bd1*^*5*^. **** P<0.0001 (t-test). (D) More pH3 positive cells are detected around *ebd1* mutant clones than around control clones, indicating that the rate of cell division is higher around the mutant clones. (E-H) RNAi-mediated disruption of *ebd1* expression, specifically in ECs, leads to increased numbers of progenitors (compare F to E, and H to G). Progenitor cells are identified as small cells with strong Arm staining and lack of Prospero staining (magenta). (I-K) RNAi-mediated disruption of *ebd1* expression, specifically in ECs, leads to increased numbers of ISCs (compare J and K to I). ISCs are stained with Dl (magenta) and EEs with Prospero (magenta). (L) Increased rates of ISC proliferation are observed when *ebd1* expression is knocked down with three independent RNAi lines. Proliferation rate is assessed by the number of pH3 positive cells in the posterior midgut (PMG). **** P<0.0001, ** P<0.01, * P<0.05 (t-test). Scale bars in (A-B’) and (E-K) are 10 μm. Genotypes: (A-B’) **control**: *y w hs-flp/+; tub-Gal4 UAS-GFP/esg-lacZ; FRT2A tub-Gal80/FRT2A* ***ebd1 (ebd1***^***240***^***)*:** *y w hs-flp/+; tub-Gal4 UAS-GFP/esg-lacZ; FRT2A tub-Gal80/ebd1*^*240*^ *FRT2A* ***ebd1***^***5***^: *y w hs-flp/+; tub-Gal4 UAS-GFP/esg-lacZ; FRT2A tub-Gal80/ebd1*^*5*^ *FRT2A* (E-K) ***ebd1***^***i1***^: *UAS-ebd1 RNAi#1/+* ***Myo>ebd1***^***i1***^: *Myo1A-Gal4 UAS-GFP tub-Gal80*^*ts*^*/+; UAS-ebd1 RNAi#1/+* ***ebd1***^***i2***^: *UAS-ebd1 RNAi#2/+* ***Myo>ebd1***^***i2***^: *Myo1A-Gal4 UAS-GFP tub-Gal80*^*ts*^*/+; UAS-ebd1 RNAi#2/+* ***Myo>+*:** *Myo1A-Gal4 UAS-GFP tub-Gal80*^*ts*^*/+* ***Myo>ebd1***^***i3***^: *Myo1A-Gal4 UAS-GFP tub-Gal80*^*ts*^*/+; UAS-ebd1 RNAi#3/+* (L) **WT:** *Myo1A-Gal4 UAS-GFP tub-Gal80*^*ts*^*/+* ***ebd1***^***i1***^: *Myo1A-Gal4 UAS-GFP tub-Gal80*^*ts*^*/+; UAS-ebd1 RNAi#1/+* ***ebd1***^***i2***^: *Myo1A-Gal4 UAS-GFP tub-Gal80*^*ts*^*/+; UAS-ebd1 RNAi#2/+* ***ebd1***^***i3***^: *Myo1A-Gal4 UAS-GFP tub-Gal80*^*ts*^*/+; UAS-ebd1 RNAi#3/+*.

The activation of Wingless signaling in ECs inhibits the proliferation of ISCs non-autonomously to regulate adult intestinal homeostasis [89,92]. Similarly, we found that an abnormally large number of progenitor cells were clustered around *ebd1*^*240*^ or *ebd1*^*5*^ null mutant clones (Fig 8A–8B’; quantification: Fig 8C). The excess progenitor cells present near *ebd1* clones resulted from aberrantly increased proliferation, as revealed by the number of pH3 positive cells (Fig 8D). Since Wingless signaling is required specifically in ECs to regulate the proliferation of surrounding ISCs [92], we tested whether Ebd1 functions similarly by reducing *ebd1* expression in ECs using RNAi-mediated knockdown with the EC-specific driver *Myo1A-Gal4* [83]. As compared with controls, knockdown of *ebd1* in ECs resulted in increased numbers of progenitor cells (marked by *esg-lacZ* or a combination of Arm and Prospero staining) and ISCs (marked by Dl) that were present in chains or grouped in clusters (Fig 8E–8K and S18A–S18F Fig). Furthermore, the number of pH3-positive cells increased upon *ebd1* knockdown in ECs, confirming the overproliferation of ISCs (Fig 8L). Moreover, as reported previously for inactivation of other Wingless pathway components [92], increased ISC proliferation was observed when *ebd1* expression was disrupted during adulthood, but not during development (S18G–S18I Fig). These results were obtained with three independently derived transgenic *ebd1* RNAi lines to rule out the possibility of off-target effects. Together, our findings suggest that loss of *ebd1*, like that of Wingless pathway components, non-autonomously promotes the proliferation of neighboring ISCs.

**Fig 9:**
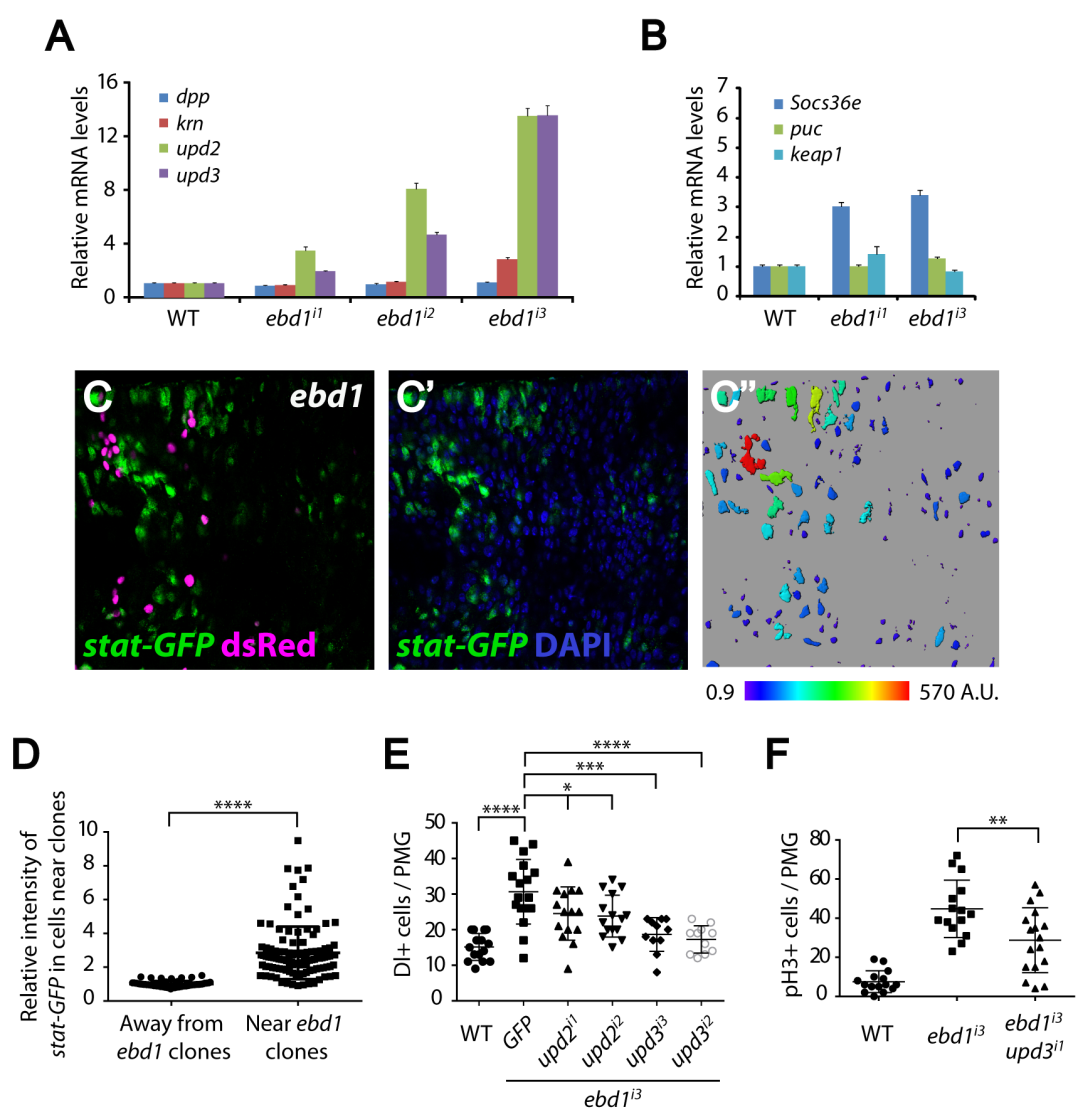
Loss of *ebd1* non-autonomously activates the Jak/Stat pathway to regulate ISC proliferation. (A) Expression of the Jak/Stat pathway ligands, *upd2* and *upd3*, increases upon RNAi-mediated knockdown of *ebd1* expression in ECs. Expression of two other ligands secreted by ECs, *dpp* and *krn*, is not affected by the loss of *ebd1*. (B) Increased expression of *Socs36e*, a downstream target of Jak/Stat signaling, is observed upon RNAi-mediated knockdown of *ebd1*. Conversely, knockdown of *ebd1* has no effect on expression of *puc* or *keap1*, indicating that the stress pathways are not activated and that Jak/Stat pathway induction is not a secondary defect due to the induction of a stress response. (C-D) Expression of the Jak/Stat pathway reporter, *stat-GFP* (green, C and C’), is higher near *ebd1* mutant clones (dsRed labeled clones in magenta, C) than far from the clones. Nuclei are marked with DAPI (blue). (C”-D) Imaris was used to quantify *stat-GFP* intensity near and away from *ebd1* mutant clones. **** P<0.0001 (Mann-Whitney test). (E) Concomitant knockdown of *upd2* or *upd3*, as well as *ebd1*, rescues the *ebd1* RNAi phenotype, as measured by Dl positive cells. This indicates that ectopic expression of *upd* in ECs is responsible for overproliferation of ISCs in *ebd1* mutants. **** P<0.0001, *** P<0.001, * P<0.05 (t-test). (F) Concomitant RNAi-mediated knockdown of *upd3* and *ebd1* rescues the *ebd1* RNAi-mediated ISC overproliferation phenotype, as measured by pH3 staining. ** P<0.01 (t-test). Scale bars: (C-C”) 50 μm. Genotypes: (A-B) **WT:** *Myo1A-Gal4 UAS-GFP tub-Gal80*^*ts*^*/+* ***ebd1***^***i1***^: *Myo1A-Gal4 UAS-GFP tub-Gal80*^*ts*^*/+; UAS-ebd1 RNAi#1/+* ***ebd1***^***i2***^: *Myo1A-Gal4 UAS-GFP tub-Gal80*^*ts*^*/+; UAS-ebd1 RNAi#2/+* ***ebd1***^***i3***^: *Myo1A-Gal4 UAS-GFP tub-Gal80*^*ts*^*/+; UAS-ebd1 RNAi#3/+* (C-D) *y w hs-flp tub-Gal4 UAS-dsRed/+; 10xstat-GFP/+; FRT2A tub-Gal80/ebd1*^*240*^ *FRT2A* (E) **WT:** *Myo1A-Gal4 UAS-GFP tub-Gal80*^*ts*^*/+* ***ebd1***^***i3***^ **GFP:** *Myo1A-Gal4 UAS-GFP tub-Gal80*^*ts*^*/+; UAS-ebd1 RNAi#3/UAS-GFP-lacZ* ***ebd1***^***i3***^ ***upd2***^***i1***^: *Myo1A-Gal4 UAS-GFP tub-Gal80*^*ts*^*/+; UAS-ebd1 RNAi#3/UAS-upd2 RNAi#1* ***ebd1***^***i3***^ ***upd2***^***i2***^: *Myo1A-Gal4 UAS-GFP tub-Gal80*^*ts*^*/+; UAS-ebd1 RNAi#3/UAS-upd2 RNAi#2* ***ebd1***^***i3***^ ***upd3***^***i1***^: *Myo1A-Gal4 UAS-GFP tub-Gal80*^*ts*^*/UAS-upd3 RNAi#1; UAS-ebd1 RNAi#3/+* ***ebd1***^***i3***^ ***upd3***^***i2***^: *Myo1A-Gal4 UAS-GFP tub-Gal80*^*ts*^*/+; UAS-ebd1 RNAi#3/UAS-upd3 RNAi#2* (F) **WT:** *Myo1A-Gal4 UAS-GFP tub-Gal80*^*ts*^*/+* ***ebd1***^***i3***^: *Myo1A-Gal4 UAS-GFP tub-Gal80*^*ts*^*/+; UAS-ebd1 RNAi#3/+* ***ebd1***^***i3***^ ***upd3***^***i1***^: *Myo1A-Gal4 UAS-GFP tub-Gal80*^*ts*^*/UAS-upd3 RNAi#1; UAS-ebd1 RNAi#3/+*.

As Wingless signaling controls the proliferation of ISCs through the Jak/Stat pathway [92], we examined whether Ebd1 analogously controls Jak/Stat signaling. RNAi-mediated knockdown of *ebd1* expression in ECs led to significant increases in the expression of *upd2* and *upd3*, ligands for the Jak/Stat pathway (Fig 9A). In contrast, little increase was detected in the expression of *decapentaplegic* (*dpp*) or *keren* (*krn*), EC-expressed ligands for the TGF-β and EGF pathway, respectively [106–109] (Fig 9A). Similarly, expression of *puckered* (*puc*) and *keap1*, target genes for the two major stress response signaling pathways, JNK (c-Jun N-terminal kinase) and Nrf2 (Nuclear factor 2) respectively [110–112], was not affected (Fig 9B). Thus, Ebd1 specifically regulates the expression of Jak/Stat pathway ligands in ECs, and could thereby control the activation of Jak/Stat signaling in adjacent ISCs. In support of this idea, we found that RNAi-mediated knockdown of *ebd1* in ECs induced expression of *Socs36e*, a direct target gene of the Jak/Stat pathway [113] (Fig 9B). To further test whether Jak/Stat signaling is activated in ISCs upon loss of *ebd1* in ECs, we analyzed the expression of the Jak/Stat pathway reporter, *stat-GFP* [114]. We found that *stat-GFP* expression increased markedly in ISCs near *ebd1* mutant clones (Fig 9C–9C”; quantification: Fig 9D), indicating that the Jak/Stat pathway was activated non-autonomously upon *ebd1* inactivation. To determine whether the ectopic activation of Jak/Stat signaling mediates the overproliferation of ISCs resulting from loss of *ebd1*, we concomitantly knocked down both *upd* and *ebd1* in ECs using RNAi. Dual knockdown of *ebd1*, and either *upd2* or *upd3*, reduced ISC proliferation in posterior midguts, as indicated by Dl and pH3 staining (Fig 9E and 9F). Therefore, Ebd1 activity in ECs, like that of Wingless pathway components, prevents the non-autonomous activation of JAK/STAT signaling in neighboring ISCs, and thereby inhibits their proliferation.

The observation that *ebd1* inactivation results in ISC overproliferation in physiological conditions, but prevents ISC overproliferation in *Apc1* mutants presented us with a paradox. Analysis of the spatial and temporal requirement for Ebd1 provided an explanation for these unanticipated results. The *Apc1* mutant phenotype emerges during formation of the adult gut during pupation (S3–S5 Figs and S19A, S19C and S19E Fig), a stage in which *ebd1* knockdown has no effect (S18G–S18I Fig). Indeed, the midguts of newly eclosed *ebd1* mutants exhibited a similar number of EBs by comparison with the age-matched controls (S19A and S19B Fig; quantification: S19E Fig). Furthermore, Ebd1 is non-autonomously required in ECs to prevent ISC overproliferation during adult homeostasis (Figs 8 and 9 and S18 Fig), in contrast to its autonomous requirement in progenitor cells for the gut defects resulting from *Apc1* loss (Fig 4). Together, these findings indicate that Ebd1 plays qualitatively different roles in transducing physiological and pathological Wingless signaling, which are temporally and spatially distinct.

**Fig 10:**
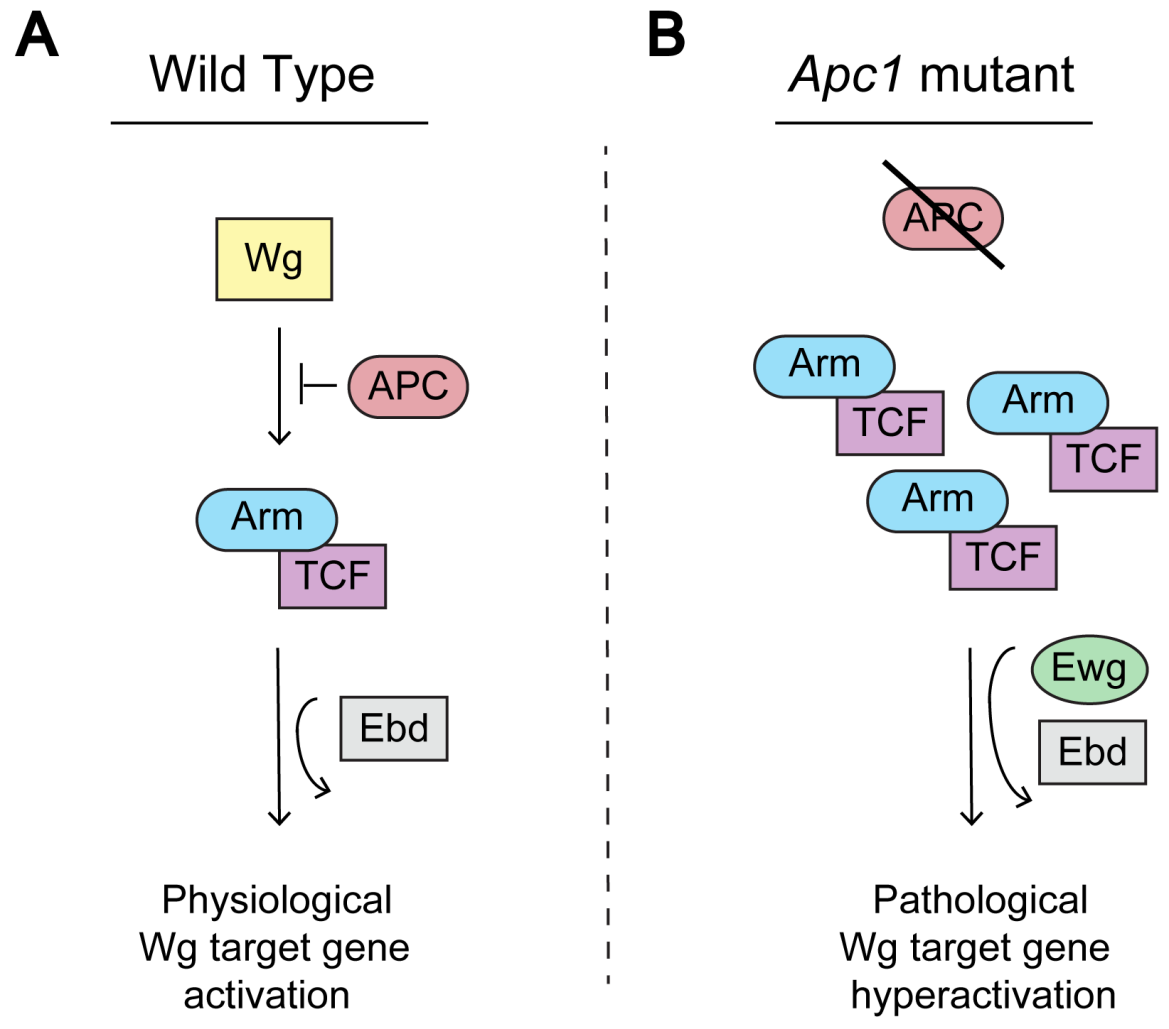
Model for Ebd and Ewg function in Wnt signaling. (A) Ebd, but not Ewg, is required for Wingless-dependent regulation of midgut homeostasis under physiological conditions. (B) Both Ebd and Ewg are required for high-level transcription of Wingless target genes and consequent ISC overproliferation and intestinal hyperplasia following *Apc1* inactivation.

In summary, our analysis of two transcription cofactors, Ebd and Ewg, in the Drosophila midgut revealed that both Ebd and Ewg are required for all major consequences of *Apc1* inactivation: the hyperactivation of Wingless target genes, excess number of progenitor cells, and epithelial hyperplasia (Fig 10A). By contrast, during intestinal homeostasis, only Ebd, but not Ewg, is essential for the Wingless-dependent control of ISC proliferation (Fig 10B). Together, these findings provide evidence that some context-specific transcription cofactors are differentially required for physiological Wnt pathway activation during homeostasis versus the oncogenic hyperactivation of the Wnt pathway resulting from loss of *Apc1*, and thus may present opportunities for the therapeutic targeting of Wnt-driven diseases.

## Discussion

Our findings indicate that both Ebd and Ewg are necessary for the aberrantly high-level Wnt target gene activation that mediates the consequences of *Apc1* loss. These results provide *in vivo* evidence that the core β-catenin-TCF transcriptional machinery is insufficient for the transformation of intestinal epithelial cells in *Apc1* mutants; cooperation of β-catenin-TCF with Ebd and Ewg is also necessary. As Ebd and Ewg are known to physically interact with each other and with β-catenin, we postulate that the Ebd-Ewg complex acts with β-catenin-TCF to activate the high-level transcription of Wnt target genes in ISCs in *Apc1* mutants. Moreover, Ebd, but not Ewg, is required for the Wnt-dependent control of ISC proliferation during homeostasis. Together, these studies reveal that transcription cofactors with context-specific roles in Wnt target gene activation under physiological conditions can be co-opted to function with β-catenin-TCF to promote the global hyperactivation of Wnt target genes following *APC* loss.

### Ebd and Ewg are essential mediators of the hyperactivated Wnt target gene expression and intestinal epithelial defects that result from *Apc1* inactivation.

In both mammals and Drosophila, two *APC* paralogs have partially redundant roles that are dependent on cell context. However, as in humans and mice, inactivation of a single Drosophila *APC* homolog alone is sufficient to induce ISC overproliferation, as well as defects in intestinal epithelial cell adhesion, cell polarity, and intestinal architecture that recapitulate many aspects of human colorectal cancer. Furthermore, similar to inactivation of human and mouse *APC*, loss of Drosophila *Apc1* results in aberrantly high levels of Wnt target gene expression in the intestine. Our analysis of one Wnt target gene reporter reveals a fivefold increase in its expression in *Apc1* mutant cells compared to wild-type cells even at intestinal compartment boundaries, which are the sites with the highest levels of Wingless protein and the highest activation of physiological Wingless signaling in the adult gut. Overall, the expression of approximately 1000 genes is significantly deregulated in *Apc1* mutant guts. These results provide evidence that inactivation of Drosophila *Apc1* singly results in intestinal hyperplasia and Wingless target gene hyperactivation, in a manner analogous to the pathological consequences that result from loss of mammalian *APC*.

Our findings also reveal that Ebd and Ewg mediate the intestinal epithelial defects and oncogenic levels of Wnt target gene expression that result from loss of *Apc1*. In addition, we find that although Ewg is a known sequence-specific DNA-binding protein and is required following *Apc1* loss for the high level expression of the Wnt target genes *fz3*, *nkd*, and *notum* through their well-characterized enhancers, Ewg consensus DNA binding sites are present in only one of these three enhancers. Therefore, the direct association of Ewg with DNA might not be required for Ewg’s role in the hyperactivation of Wnt signaling, or Ewg might also interact with non-consensus binding sites. Thus, in these contexts, Ebd might access DNA through its own CENPB-type DNA binding domains [53,115]. Alternatively, these findings raise the possibility that Ewg and Ebd access chromatin via protein-protein interactions instead of direct association with DNA. A precedent for this type of mechanism was documented previously for Fushi tarazu, which activates transcription even when its DNA-binding homeodomain is deleted, through interaction with the DNA-binding transcription factor Paired [116,117].

### Ebd, but not Ewg, is essential for Wingless-dependent control of ISC proliferation during homeostasis

In the Drosophila intestine, activation of Wingless signaling in ECs non-autonomously restricts the proliferation of surrounding ISCs during homeostasis. Our findings herein suggest that this process requires Ebd. We further find that Ebd is also required for the autonomous hyperactivation of Wingless signaling in ISCs that results in their overproliferation following *Apc1* loss. This novel finding reveals that Ebd is required for Wnt signaling during both normal homeostasis of the intestine and its aberrant hyperplasia, in addition to Ebd’s previously documented roles in muscles and neurons [53,54]. Similar to that in other tissues, the role of Ebd in the intestine is context-specific, as not all Wnt-mediated processes are dependent on Ebd; Ebd promotes the Wnt-mediated regulation of ISC proliferation during homeostasis, but is dispensable for the Wnt-dependent specification of cell fate near intestinal compartment boundaries [92]. Conversely, Ewg has no observed role in either of these Wnt-dependent processes. Thus, Ebd functions in an Ewg-independent manner in the adult gut under physiological conditions. These results suggest that Ebd and Ewg do not always function in a complex, and that recruitment of Ebd to chromatin by Ewg [54] is context-specific.

### Context-specific transcription cofactors in Wnt pathway hyperactivation: Implications for new therapeutic strategies in Wnt-driven cancer

Based on our findings, we propose that mechanistic differences in the Wnt transcriptional machinery underlie target gene activation in physiological versus pathological states. These novel distinctions likely underlie the markedly increased expression of Wingless target genes in the hyperactivated state that results from *Apc1* inactivation. Analogously, the mammalian transcription cofactors Pygo and BCL9 also form a complex that enhances target gene activation by β-catenin-TCF in the Wnt hyperactivated state. Neither mammalian Pygo nor BCL9 is required for Wnt-mediated ISC proliferation or maintenance during homeostasis, but both promote Wnt target gene expression in colorectal cancer. Most targeted therapies under investigation disrupt Wnt signaling not only in tumors, but also in normal tissues. Thus, the discovery that transcription cofactor complexes, such as Ebd-Ewg in Drosophila or Pygo-BCL9 in mammals, are essential for supraphysiological signaling but dispensable for most Wnt-dependent physiological processes may distinguish tumors from normal tissues and provide selectivity for therapeutic strategies that target Wnt-driven diseases.

Our findings suggest that the human homologs of Ebd and Ewg might provide novel therapeutic targets for the treatment of Wnt-driven cancers. Jerky (also known as JRK or JH8; [118–123]), the human homolog of Ebd, rescues *ebd* mutant phenotypes when expressed in Drosophila [53] and promotes the aberrant increase of both cell proliferation and β-catenin-TCF mediated transcription in colon cancer cell lines [53,124,125]. Moreover, aberrantly high levels of Jerky are present in several carcinomas, including colon, breast, and ovarian serous cystadenocarcinoma. Elevated Jerky expression is associated with increased β-catenin nuclear localization and the aberrantly increased expression of Wnt target genes in human colorectal tumors [125]. A possible role for Nuclear Respiratory Factor 1 (NRF1), the human homolog of Ewg, in Wnt signaling awaits future investigation. Together, these findings suggest that inhibition of Jerky, NRF1, or their physical interaction may provide promising therapeutic strategies for colorectal cancer.

## Materials and methods

### Fly stocks

**Reporters:** *esg>GFP* (*esg-Gal4 UAS-GFP)* [58], *GBE-Su(H)-lacZ* [58], *fz3-RFP* [95], *notum-lacZ* [93,94], *nkd(UpE2)-lacZ* [97], *esg-lacZ* [58], and *10x stat-GFP* (destabilized) [114].

**Mutant alleles:** *Apc1*^*Q8*^ [67], *ebd1*^*240*^ [53], *ebd1*^*5*^ [53], *ebd2*^*136*^ [53], *Df(3L)9698* [53], *ewg*^*P1*^ [54], *ewg*^*2*^ [55], *ewg*^*1*^ [55], *fz*^*H51*^ *Dfz2*^*C1*^ [102], *and dsh*^*3*^ [103].

**MARCM lines:** MARCM 82B: *y w hs-flp UAS-CD8*::*GFP; tub-Gal4 FRT82B tub-Gal80/TM6B* [126], MARCM 2A: *y w hs-flp; tub-Gal4 UAS-mCD8*::*GFP*^*LL5*^*/CyO act-GFP*^*JMR1*^*; FRT2A tub-Gal80*^*LL9*^ [127], or *y w hs-flp; tub-Gal4 UAS-GFP; FRT2A tub-Gal80/TM6B* (A kind gift from the Ohlstein lab), or *y w hs-flp tub-Gal4 UAS-dsRed; FRT2A tub-Gal80*, MARCM 19A: *hs-flp tub-Gal80 FRT19A;; tub-Gal4 UAS-mCD8*::*GFP/SM6^TM6B* [128].

**RNAi lines and Gal4 drivers:** *Myo1A-Gal4* [129], *ebd1-Gal4* [53] (DGRC#104336), *esg-Gal4 tubGal80*^*ts*^ *UAS-GFP/CyO*, *esg*^*ts*^ *F/O* (*esg-Gal4 tub-Gal80*^*ts*^ *UAS-GFP; UAS-flp Act>CD2>Gal4*) [83], *UAS-GFP-lacZ* (BDSC#6452), *w1118* (BDSC#5905), *UAS-Apc1 RNAi#1* (VDRC#51469; Construct ID: 1333), *UAS-Apc1 RNAi#2* (VDRC51468; Construct ID: 1333), *UAS-ewg RNAi#1* (BDSC#31104), *UAS-ewg RNAi#2* (BDSC#31225), *UAS-ebd1 RNAi#1* (VDRC#26180; Construct ID: 10952), *UAS-ebd1 RNAi#2* (BDSC#35765), *UAS-ebd1 RNAi#3* (BDSC#28296), *UAS-upd2 RNAi#1* (BDSC#33988), *UAS-upd2 RNAi#2* (BDSC#33949), *UAS-upd3 RNAi#1* (VDRC#27136; Construct ID: 6811), *UAS-upd3 RNAi#2* (BDSC#28575).

Canton S flies were used as wild-type controls. Fly crosses were performed at 25°C unless otherwise indicated.

### Immunohistochemistry

Primary antibodies were chicken anti-GFP (Abcam, Cat. no. ab13970, 1:10000), rabbit anti-GFP (Thermo Fisher Scientific, Cat. no. A-11122, 1:500), mouse anti-Arm [Developmental Studies Hybridoma Bank (DSHB), N2 7A1, 1:20], mouse anti-Discs large (DSHB, 4F3, 1:20), mouse anti-Prospero (DSHB, MR1A, 1:100), mouse anti-Delta (DSHB, C594.9B, 1:100), rabbit anti-dsRed (Clontech/TaKaRa, Cat. no. 632496, 1:500), mouse anti-β-gal (Promega, Cat. no. Z378B, 1:500), rabbit anti-β-gal (MP Biomedicals, Cat. no. 08559762, 1:5000), rabbit anti-phospho-histone H3 (Ser10) (Millipore, Cat. no. 06–570, 1:1000), rabbit anti-phospho-histone H3 (mix 1–1 of Cell signaling, Cat. no. 9701 (Ser10) and Cat. no. 9713 (Ser28), 1:100), guinea pig anti-Ebd1 ([53], 1:1000), Alexa Fluor 555 phalloidin (Thermo Fisher Scientific, Cat. no. A34055, 1:500) and DAPI (Sigma, 1:400). Secondary antibodies were goat or donkey Alexa Fluor 488 or 555 conjugates (Thermo Fisher Scientific, 1:400), and goat or donkey Cy5 conjugates (Thermo Fisher Scientific/Jackson Immunochemicals, 1:200).

Adult fly intestines were dissected in PBS and fixed in 4% paraformaldehyde in PBS for 45 minutes at room temperature. For Delta staining, intestines were fixed in 8% paraformaldehyde, 200mM Na cacodylate, 100mM sucrose, 40 mM KOAc, 10 mM NaOAc, and 10mM EGTA for 20 minutes at room temperature [130]. Tissues were then washed with PBS, 0.1% Triton X-100, followed by incubation in PBS, 0.1% Tween-20 and 10% BSA for 1 hour at room temperature. The samples were then incubated with primary antibodies at 4°C overnight in PBS, 0.5% Triton X-100. Samples were stained with secondary antibodies for 2 hours at room temperature. Specimens were stained with DAPI (2μg/ml) and mounted in Prolong Gold (Invitrogen). To assess the gut layers, specific mounting set-ups were performed according to a protocol from the Micchelli lab [74]. Larval guts were immunostained in the same way except that wandering third instar larvae were fixed in 4% paraformaldehyde in PBS for only 20 minutes and were incubated with primary antibodies in PBS, 0.1% Triton X-100. Fluorescent images were obtained on a Nikon A1RSi confocal microscope except those in (S6 Fig), which were captured on a Zeiss LSM 780 confocal microscope. Images were processed using Adobe Photoshop software.

### Clonal analysis

Mitotic clones were generated using the MARCM system [99]. Developmental clones were induced in third instar larvae by a single 2–3 hour heat shock at 37°C and examined 1 to 2 days after eclosion. To generate clones in the adult gut, flies were heat shocked for 30 minutes in a 37°C water bath four days post-eclosion. After heat shock, flies were maintained at 25°C for five days before analysis. For quantification of clone size, flies were maintained at 25°C for 14 days post heat shock and only clones in the posterior midguts were included in the analysis.

### Transgene expression using temperature-sensitive Gal4 and flip out systems

To induce temporal knockdown in ISCs, control or specific RNAi lines were crossed to the *esg*^*ts*^ (*esg-Gal4 tubGal80*^*ts*^ *UAS-GFP/CyO*) driver. For knock down during development, crosses were set up at 18°C and shifted to 29°C 6 days later (during the second instar larval stage). Progeny of desired genotypes were dissected 2–3 days after eclosion. For knock down during adulthood, crosses were maintained at 18°C until eclosion, and progeny of desired genotypes were then shifted to 29°C for 14 days before analysis.

To induce temporal knockdown in ECs, RNAi experiments were performed using *Myo1A-Gal4* with the temperature-sensitive Gal4 repressor, *Gal80*^*ts*^. Crosses were maintained at 22°C and on the day of eclosion, progeny of desired genotypes were shifted to the restrictive temperature (29°C) for 7 days.

To induce temporal knockdown using the “escargot flip out” system (*esg*^*ts*^ *F/O*: *esg-Gal4 tub-Gal80*^*ts*^ *UAS-GFP; UAS-flp Act>CD2>Gal4*), crosses were maintained at 18°C and 3-5-day-old progeny of the desired genotypes were shifted to 29°C. The marked *esg*^*+*^ cell lineages were analyzed 14 days later.

### Quantification and statistics

For quantification of ISCs, flies were stained with anti-Delta (Dl) and anti-Prospero antibodies. Images of the midgut R5a region [89] were obtained with a 60x lens and the total number of Dl-positive cells in a field of 0.051mm^2^ was counted. Similarly, progenitor cells inside a defined field were quantified by counting *esg>GFP*, *esg-lacZ*, or small cells with strong Arm staining and absence of Prospero staining. EBs inside a defined field were quantified by counting *GBE-Su(H)-lacZ* positive cells. For quantification of pH3-positive cells, the total number of pH3-positive cells in the posterior midgut of the indicated genotypes was counted. For quantification of pH3-positive cells near MARCM clones, the number of pH3-positive cells in a field of 4000 µm^2^ around the clone was counted.

For quantification of intestinal structure, 40x Z-stack confocal images of posterior midguts of desired genotypes were acquired. The maximum number of epithelial layers and maximum epithelial height were measured using NIS-elements software (Nikon).

Quantification of GFP intensity in “*esg*^*ts*^ flip out” guts was performed by measuring overall GFP intensity within two areas per posterior midgut and normalizing that value by the total number of cells in the field.

Quantification of *fz3-RFP* intensity was performed with ImageJ (NIH). For each clone examined (total of 57 clones derived from more than 20 guts), intensities of three separate areas within the clone and areas of identical size outside the clone were measured. The average intensities of the three areas were compared to the average intensities of their control counterparts.

Quantification of *stat-GFP* intensity was performed using Imaris software (Bitplane). *Stat-GFP*-positive cells within a field (40μm × 40μm) surrounding an *ebd1* mutant clone, or in an equal field at least 50μm away from the *ebd1* mutant clone, were identified and their intensity was measured.

All statistical tests were performed using Prism (GraphPad Software, USA).

### Microarray

Whole midguts from Canton S (control) or *Apc1*^*Q8*^ 7-day-old females were dissected in nuclease-free PBS and processed for transcriptomic analysis. Total RNA from 30 adult midguts per sample was extracted using Trizol following manufacturer’s instructions. Triplicate samples of each of the genotypes were prepared. RNA was sent to the Microarray facility at the University of Manchester where it was used to hybridize Drosophila Affymetrix 2.0 chips. The CEL files were subject to RMA normalization and log2 transformation followed by differential gene expression analysis by the Beatson Institute Bioinformatics department. Microarray data were deposited in the GEO database (GSE99071). GO term analysis was performed via “PANTHER GO-slim” [131].

### RT-qPCR validation

Whole midguts from 15–20 flies of desired genotypes were dissected in PBS and total RNA was extracted using the RNA miniprep kit (Zymo research). The RNA was subsequently treated with RQ1 DNase (Promega). 1 μg of RNA was reverse transcribed using pdT15 primers and M-MLV reverse transcriptase (Invitrogen). Expression level of candidate genes was quantified using the StepOne Real-time PCR system (Life Technologies) with SYBR green (Life Technologies/Biorad). RNA extraction of three biologically independent samples was performed. Two independent repeats are presented in Fig 3 and S10 Fig, respectively, as mean fold change relative to the internal control (rpl32), with standard deviation. The primers used are listed in Table 1.

**Table 1:** Primers for RT-qPCR validation.

Primer name	Sequence	Reference
rpl32_F	AGG CCC AAG ATC GTG AAG AA	[73]
rpl32_R	TTG TGC ACC AGG AAC TTC TTG AA	[73]
Socs36e_F	ATG ACC GTG CAC TCG CAA AT	[73]
Socs36e_R	CCT CGT AGC GGT CCA TCT TG	[73]
CG7997_F	GGC TGA ATC CTG TCC TGA T	This study
CG7997_R	CTG CTC AAT GAA ACT GGT CG	This study
sog_F	TTG CCC TGC TCC TCA ATC	This study
sog_R	GCT GCG AAA TCT TCC AGA C	This study
Akap200_F	TCT GAC CAC CGA AAA GAG T	This study
Akap200_R	CTT GCC AAA GGA AAT GCT TC	This study
CG6234_F	CTG GTT TCC GTG ATT CTC AAC	This study
CG6234_R	CTC CTC ATA AAC TGG CAC TG	This study
kay_F	CGA GGT GCT GAC GAA TAG C	This study
kay_R	TGT GGT TGT GAT GGC TGC	This study
vein_F	GTG AAG TTG CCT GGA TTC GT	[73]
vein_R	CTA CAG GGA GCG ACT GAT GC	[73]
spitz_F	TAC CAG GCA TCG AAG GTT TC	[73]
spitz_R	GAC CCA GGC TCC AGT CAC TA	[73]
CG31259_F	TTT GCC GTG TGC TAC TTG	This study
CG31259_R	TCG TGC CTC CAT CAT CTT	This study
DopEcR_F	CAG GAC ACC CAG AAT GC	This study
DopEcR_R	TCG TAC ACA ACT ACG GCT ATA	This study
AR-2_F	GTA ATG TTC TCC GTC CTC ATC	This study
AR-2_R	TCC AGT ACC TGA TTG TGG T	This study
Pdm2_F	AAT GAG TAG TGC CGT GAT GA	This study
Pdm2_R	CCG CTG TTT GAA GGT CTT	This study
nkd_F	GCC ACG CCA GCA AAC TGA AGA	This study
nkd_R	TGC GTG GTC GAT AGC GAT GCT	This study
notum_F	AGA GCA GCA GAA GCG TTA GC	This study
notum_R	AAA GCC GGA GAA GCT ACA AA	This study
fz3_F	ACA GTG AAG AGT AGT GGT CG	This study
fz3_R	CCA CCT CCT GTG GAA TCT GC	This study
CG16749_F	GAA TGG CTC CTC CAC GA	This study
CG16749_R	TGC TGC TCA TTG TAC GGA TG	This study
hh_F	CGT TCA TCA CCG AGT AGG C	This study
hh_R	CAA CTA CAA CAG GGA CAT CCT T	This study
CG32407_F	AAC CGC TGC TGA TCC TC	This study
CG32407_R	CCA TGT CCA AGT TAC TCA GAC C	This study
upd3_F	CCG TCT GAA TCT CAC TAG CAA AC	This study
upd3_R	TTC CGC ATC CTT CCC AG	This study
lectin-24A_F	TCC TGG CTG TAG GCA TTG A	This study
lectin-24A_R	ACA GTG AAA CTG GAC AGA ACG	This study
CG3961_F	TTG AAG ACC AAC TTG TCC CA	This study
CG3961_R	GCT GCG AGA ATG GAA TGT ACT A	This study
ewg_F	CCA CAA GCT ATC GGC TAG TCG	[132]
ewg_R	GCC CCA TTC GAG GAG TGA C	[132]

## Supporting information

S1 Fig Loss of *Apc1* alone is sufficient to induce excess progenitor cells and intestinal hyperplasia. (A-B) Dramatically increased numbers of *esg>GFP* marked progenitor cells are detected in *Apc1* mutants.
(C-D) Arm localization, which defines cell-cell junctions in the intestinal epithelium, is severely altered in *Apc1* mutants: the small progenitor cells form chains and clusters with strong Arm staining, whereas the large ECs have much less membrane-associated Arm.
(E-F) By contrast to the monolayer intestinal architecture in controls, *Apc1* mutant guts display extensive multi-layering and epithelial hyperplasia (cross-sectional view).
Scale bars: 50 μm.
Genotypes: **control:** *esg-Gal4 UAS-GFP/+; Apc1*^*Q8*^*/+*
***Apc1*:** *esg-Gal4 UAS-GFP/+; Apc1*^*Q8*^.
(TIF)

S2 Fig Ebd and Ewg mediate *Apc1* mutant intestinal stem cell phenotypes. (A) Quantification of *esg>GFP* positive progenitor cells in control, *Apc1* mutants, *ebd Apc1* mutants and *ewg Apc1* mutants. Increased number of progenitor cells in *Apc1* mutants is rescued by concomitant loss of *ebd* or *ewg*. **** P<0.0001 (Mann-Whitney test).
(B-D) Inactivation of *Apc1* leads to dramatically increased numbers of EBs, marked with *GBE-Su(H)-lacZ* (green; compare C to B). This defect is suppressed in *ebd Apc1* double mutants (D).
(E) In contrast to the control guts, which exhibit a monolayer intestinal architecture, the *Apc1* mutant guts are multi-layered. This defect is rescued in *ebd Apc1* or *ewg Apc1* double mutants. **** P<0.0001 (t-test).
(F) Compared to controls, the maximum epithelial height is greatly increased in *Apc1* mutants. The height is reverts to a normal level upon concomitant loss of *ebd* or *ewg*. **** P<0.0001 (t-test).
Scale bars: (B-D) 10 μm.
Genotypes: (A) **control:** *esg-Gal4 UAS-GFP; Apc1*^*Q8*^*/+*
***Apc1*:** *esg-Gal4 UAS-GFP; Apc1*^*Q8*^
***ebd Apc1*:** *esg-Gal4 UAS-GFP; ebd1*^*240*^ *Apc1*^*Q8*^*/Df(3L)9698 ebd2*^*136*^ *Apc1*^*Q8*^
***ewg Apc1*:** *ewg*^*P1*^*; esg-Gal4 UAS-GFP; Apc1*^*Q8*^
(B-D) **control:** *GBE-Su(H)-lacZ/+; Apc1*^*Q8*^*/+*
***Apc1*:** *GBE-Su(H)-lacZ/+; Apc1*^*Q8*^
***ebd Apc1*:** *GBE-Su(H)-lacZ/+; ebd1*^*240*^ *Apc1*^*Q8*^*/Df(3L)9698 ebd2*^*136*^ *Apc1*^*Q8*^
(E-F) **control:** *Apc1*^*Q8*^*/+*
***Apc1*:** *Apc1*^*Q8*^
***ebd Apc1*:** *ebd1*^*240*^ *Apc1*^*Q8*^
***ewg Apc1*:** *ewg*^*P1*^*; Apc1*^*Q8*^.
(TIF)

S3 Fig ISC overproliferation is readily detected in *Apc1* mutants at eclosion. Excess numbers of progenitor cells, marked by *esg>GFP* (green), are readily detected in newly eclosed *Apc1* mutant guts (compare C-C” to A-A”; high magnification view: compare D-D” to B-B”). The cell-cell junctions, which are marked by Arm (magenta), remained largely intact at this stage (compare C’ to A’; high magnification view: compare D’ to B’).
Scale bars: (A-A” and C-C”) 50 μm, (B-B” and D-D”) 10 μm.
Genotypes: **control:** *esg-Gal4 UAS-GFP; Apc1*^*Q8*^*/+*
***Apc1*:** *esg-Gal4 UAS-GFP; Apc1*^*Q8*^.
(TIF)

S4 Fig *Apc1* mutant ISC defects are not present before pupation. (A-B”) Numbers of AMPs (adult midgut progenitors), marked by *esg>GFP* (green), are comparable between control and *Apc1* mutant guts.
(C-D”) The cell-cell junctions, marked by membrane-associated Discs large 1 (Dlg1, magenta), remain intact at this stage.
Scale bars: (A-B”) 10 μm, (C-D”) 50 μm.
Genotypes: **control:** *esg-Gal4 UAS-GFP; Apc1*^*Q8*^*/+*
***Apc1*:** *esg-Gal4 UAS-GFP; Apc1*^*Q8*^.
(TIF)

S5 Fig Diminishing *Apc1* activity either during development of the adult gut or during adulthood results in excess progenitor cells. (A-D”) *Apc1* expression, knocked down using the *esg*^*ts*^ driver during formation of the adult gut (crosses were shifted from 18°C to 29°C during second instar larval stage and the progeny of desired genotype were examined 2–3 days post-eclosion), results in excess progenitor cells. Progenitor cells are identified as small cells with strong Arm staining and lack of Prospero staining (magenta) or by *esg>GFP* (green). Nuclei are labeled with DAPI (blue). Low magnification view: A-A’ (control) and C-C’ (*Apc1* RNAi); high magnification view: B-B’ (control) and D-D’ (*Apc1* RNAi).
(E-F”) *Apc1* expression, knocked down using the *esg*^*ts*^ driver during adulthood (progeny of desired genotype were shifted from 18°C to 29°C after eclosion and analyzed 14 days later), also results in excess progenitor cells (marked by *esg>GFP*, green). Nuclei are labeled with DAPI (blue). Low magnification view: E-E’ (control) and G-G’ (*Apc1* RNAi); high magnification view: F-F’ (control) and H-H’ (*Apc1* RNAi).
(I) Quantification of progenitor cell numbers when *Apc1* expression is knocked down during formation of the adult gut or during adulthood reveals dramatic increases in both contexts. **** P<0.0001 (t-test).
Scale bars: (A-A’, C-C’, E-E’ and G-G’) 50 μm, (B-B’, D-D’, F-F’ and H-H’) 10 μm.
Genotypes: ***Dcr Apc1***^***i1***^: *UAS-Dicer2/+; UAS-Apc1 RNAi#1/+*
***esg***^***ts***^***>Dcr Apc1***^***i1***^: *UAS-Dicer2/+; esg-Gal4 tubGal80*^*ts*^ *UAS-GFP/+; UAS-Apc1 RNAi#1/+*
***esg***^***ts***^***>GFP-lacZ*:** *esg-Gal4 tubGal80*^*ts*^ *UAS-GFP/+; UAS-GFP-lacZ*.
(TIF)

S6 Fig Adult-specific *Apc1* knockdown induces ISC proliferation/self-renewal in the midgut during adulthood. (A-B”) Knockdown of *Apc1* expression during adulthood (2–3 day old adults of the desired genotypes were shifted from 18°C to 29°C for 14 days before analysis) results in increased stem/progenitor cell self-renewal (marked by GFP, green) using the stem/progenitor and lineage tracing “*esg*^*ts*^ flip out” system. Nuclei are labeled with DAPI (blue). PM: posterior midgut; AM: anterior midgut; CCR: copper cell region.
(C) Quantification of ISC proliferation by pH3 scoring upon *Apc1* knockdown during adulthood. PMG: posterior midgut. **** P<0.001 (t-test). Number of guts (n): control guts: n = 14 and *Apc1*^*i1*^, *Apc1*^*i2*^ guts: n = 7.
(D) Measurement of total GFP area in posterior midguts (PM) of control and adult-specific *Apc1* RNAi driven by the “*esg*^*ts*^ flip out”system”. *** P<0.001 (t-test). For both conditions, 2 pictures in different regions of the posterior midgut were taken for each midgut, n = 5.
Scale bars: (A and B) 100 μm, (A’-A”) and (B’-B”) 50 μm.
Genotypes: ***esg***^***ts***^***>F/O***: *esg-Gal4*, *tub-Gal80*^*ts*^, *UAS-GFP/+; UAS-flp Act>CD2>Gal4*, UAS-GFP/+
***esg***^***ts***^***>UAS- Apc1***^***i***^ ***F/O***: *esg-Gal4*, *tub-Gal80*^*ts*^, *UAS-GFP/ UAS-Apc1 RNAi#2; UAS-flp Act>CD2>Gal4*, *UAS-GFP / UAS-Apc1 RNAi#1*.
(TIF)

S7 Fig Increased expression of the Wingless target genes *notum* and *fz3* resulting from *Apc1* loss requires Ebd and Ewg. Expansion of *notum-lacZ* (magenta; A and B) and *fz3-RFP* (magenta; E and F) expression upon loss of *Apc1* is suppressed by inactivation of *ebd* (C and G) or *ewg* (D and H). Anterior to the left. Scale bars: 100 μm.
Genotypes: (A-D) **control:** *notum-lacZ/+; Apc1*^*Q8*^*/+*
***Apc1*:** *notum-lacZ/+; Apc1*^*Q8*^
***ebd Apc1*:** *notum-lacZ/fz3-RFP; ebd1*^*240*^ *Apc1*^*Q8*^*/Df(3L)9698 ebd2*^*136*^ *Apc1*^*Q8*^
***ewg Apc1***: *ewg*^*P1*^*; notum-lacZ/+; Apc1*^*Q8*^
(E-H) **control:** *fz3-RFP/+; Apc1*^*Q8*^*/+*
***Apc1*:** *fz3-RFP/+; Apc1*^*Q8*^
***ebd Apc1*:** *notum-lacZ/fz3-RFP; ebd1*^*240*^ *Apc1*^*Q8*^*/Df(3L)9698 ebd2*^*136*^ *Apc1*^*Q8*^ (same gut as C)
***ewg Apc1***: *ewg*^*P1*^*; fz3-RFP/+; Apc1*^*Q8*^.
(TIF)

S8 Fig Increased expression of the Wingless target gene *nkd* resulting from *Apc1* loss requires Ewg. Expansion of *nkd(UpE2)-lacZ* expression [magenta, compare B to A (low magnification view) and E to D (high magnification view)] upon loss of *Apc1* is suppressed by further inactivation of *ewg* (C and F). Nuclei are marked with DAPI. Anterior to the left.
Scale bars: 100 μm.
Genotypes: **control:** *nkd(UpE2)-lacZ/+; Apc1*^*Q8*^*/+*
***Apc1*:** *nkd(UpE2)-lacZ/+; Apc1*^*Q8*^
***ewg Apc1*:** *ewg*^*P1*^*; nkd(UpE2)-lacZ/+; Apc1*^*Q8*^.
(TIF)

S9 Fig Analysis of gene ontology (GO) term enrichment of biological processes in *Apc1* mutant guts. A GO term analysis was performed on biological processes deregulated in *Apc1* mutant guts when compared to the wild type controls and the top enriched GO terms (p-value is p<0.05 for genes showing a minimum of 1.5 fold change) are visualized.
(TIF)

S10 Fig Up- or down-regulation of genes in the midgut upon loss of *Apc1* requires Ebd1 and Ewg. Quantitative RT-PCR of genes up- (A) or down-regulated (B) by loss of *Apc1*. Misexpression of both sets of genes is rescued in *ebd1 Apc1* and *ewg Apc1* double mutants. This is an independent biological replicate of the data shown in Fig 3.
Genotypes: **control:** *Apc1*^*Q8*^*/+*
***Apc1*:** *Apc1*^*Q8*^
***ebd Apc1*:** *ebd1*^*240*^ *Apc1*^*Q8*^
***ewg Apc1*:** *ewg*^*P1*^*; Apc1*^*Q8*^.
(TIF)

S11 Fig Direct association of Ewg with DNA might not be required for Ewg’s role in the hyperactivation of Wnt signaling. (A) Sequence of TCF core (HMG: High Mobility Group) consensus DNA binding sites, TCF Helper site, as well as Ewg consensus DNA binding sites.
(B-D) Each of the Wingless reporters contains at least one TCF HMG consensus binding site and TCF Helper site, but only *fz3-RFP* (D) has an Ewg consensus binding site.
(TIF)

S12 Fig The Ewg consensus binding site within the *fz3-RFP* reporter is conserved among sequenced Drosophila species. Using an Evoprinter analysis to identify site conservation, we found that the Ewg consensus binding site within the *fz3-RFP* reporter is 100% conserved within the melanogaster group (except that it is absent in the *Drosophila yakuba* species), and with only one substitution in the evolutionarily distant *Drosophila ananassae* and *Drosophila willistoni* species.
(TIF)

S13 Fig Ewg is dispensable for physiological Wingless signal transduction. Expression *of fz3-RFP* (A-B”) or *nkd-lacZ* (C-D”), reporters for Wingless signaling (in magenta), is retained in *ewg* null mutant clones, suggesting that Ewg is not required for Wingless-dependent expression of *fz3* or *nkd*. Clones are marked with GFP (green). Magnified views of the boxed regions in (A or C) are shown in (B-B” and D-D”), respectively.
Scale bars: (A and C) 50 μm and (B-B” and D-D”) 10 μm.
Genotypes: (A-B”) *hs-flp tub-Gal80 FRT19A/ewg*^*2*^ *FRT19A; fz3-RFP/+; tub-Gal4 UAS-mCD8*::*GFP/+*
(C-D”) *hs-flp tub-Gal80 FRT19A/ewg*^*2*^ *FRT19A; tub-Gal4 UAS-mCD8*::*GFP/nkd-lacZ*.
(TIF)

S14 Fig Ebd2 promotes intestinal stem cell homeostasis. (A-C) Progenitor cells, marked with *esg>GFP* (green, A) and observed as small cells with high levels of membrane-associated Arm and the absence of Prospero (Pros) staining (magenta, B), are either present as single cells or doublets distributed evenly in *ebd1/+* control guts.
(D-F) Chains of small *esg>GFP* positive progenitor cells (asterisk in F), with strong Arm staining, are observed in e*bd1*^*240*^ mutants.
(G-I) Flies homozygous mutant for *ebd1* and heterozygous mutant for *ebd2* (e*bd1*^*5*^ e*bd2*^*136*^*/* e*bd1*^*240*^) contain multi-cell clusters of *esg>GFP* positive cells (arrowhead in I), as well as chains of progenitor cells (asterisk).
(J-L) The prominence of clusters of *esg>GFP* positive cells is increased in flies homozygous mutant for both *ebd1* and *ebd2* (*ebd1*^*5*^ *ebd2*^*136*^*/ ebd1*^*240*^ *ebd2*^*136*^).
(M) *ebd1* and *ebd1 ebd2/ebd1* mutants have higher numbers of *esg>GFP* positive progenitor cells compared to controls. **** P<0.0001 (Mann-Whitney test).
(N) Proportion of posterior midguts containing chains and clusters of *esg>GFP* positive cells.
Scale bars: (A-L) 10 μm.
Genotypes: ***ebd1/+*:** *esg-Gal4 UAS-GFP; ebd1*^*240*^*/+*
***ebd1*:** *esg-Gal4 UAS-GFP; ebd1*^*240*^
***ebd1ebd2/ebd1*:** *esg-Gal4 UAS-GFP;* e*bd1*^*5*^ e*bd2*^*136*^*/* e*bd1*^*240*^
***ebd1ebd2*:** *esg-Gal4 UAS-GFP; ebd1*^*5*^ *ebd2*^*136*^*/ ebd1*^*240*^ *ebd2*^*136*^.
(TIF)

S15 Fig Proliferation of intestinal stem cells is increased in *ebd1* mutants. Adult *ebd1* clones are larger than control clones, indicating that Ebd1 is required for homeostasis of intestinal tissue during adulthood. Transient clones were excluded by restricting the analysis to clones of two or more cells.
Genotypes: **control:** *y w hs-flp/+; tub-Gal4 UAS-GFP+; FRT2A tub-Gal80/FRT2A*
***ebd1*:** *y w hs-flp/+; tub-Gal4 UAS-GFP/+; FRT2A tub-Gal80/ebd1*^*240*^ *FRT2A*.
(TIF)

S16 Fig Immunostaining with the Ebd1 antibody reveals that Ebd1 is expressed strongly in ECs under homeostatic condition. (A-A”) Fixed intestines were immunostained with the Ebd1 antibody and signals are detected in all intestinal epithelial cell types, including ECs (yellow arrow), progenitors (white arrow) and EEs (orange arrow).
(B-C”‘) Ebd1 staining is specifically diminished inside enterocytes in *ebd1* null mutant clones (marked with GFP).
Scale bars: (A-A”) 10 μm, (B and B’) 50 μm and (C-C”‘) 10 μm.
Genotypes: (A-A”) Canton S
(B-C”’) ***ebd1*:** *y w hs-flp/+; tub-Gal4 UAS-GFP/+; FRT2A tub-Gal80/ebd1*^*240*^ *FRT2A*.
(TIF)

S17 Fig *ebd1* enhancer-trap Gal4 line drives reporter expression in all gut epithelial cell types. (A-A”) *UAS-GFP-lacZ* driven by *ebd1-Gal4* (an enhancer-trap line in which Gal4 is inserted in the endogenous *ebd1* locus) exhibits expression in ECs (yellow arrow), progenitors (white arrow) and EEs (orange arrow).
(B-B”) No signal is detected with *UAS-GFP-lacZ* alone.
Scale bars: 10 μm.
Genotypes: (A-A”) *ebd1(104336)>U-GFP-lacZ*
(B-B”) *U-GFP-lacZ/+*.
(TIF)

S18 Fig Ebd1 non-autonomously regulates progenitor cell proliferation during adulthood. (A-F) RNAi-mediated disruption of *ebd1* expression leads to increased numbers of progenitor cells (compare D-F to A-C). Progenitor cells are marked with *esg-lacZ* (green) or are identified as small cells with strong Arm staining and lack of Prospero (Pros) staining (magenta). Flies were analyzed seven days post-eclosion.
(G-I) The number of progenitor cells in flies analyzed one day post-eclosion is not increased, indicating that Ebd1 regulates stem cell proliferation non-autonomously in adults, but not during development.
Scale bars: 10 μm.
Genotypes: **control:** *Myo1A-Gal4 UAS-GFP tub-Gal80*^*ts*^*/esg-lacZ*
***Myo>ebd1***^***i3***^: *Myo1A-Gal4 UAS-GFP tub-Gal80*^*ts*^*/esg-lacZ; UAS-ebd1 RNAi#3*.
(TIF)

S19 Fig The roles of Ebd in physiological versus pathological Wingless signaling are qualitatively different. (A-D) Compared with controls (A), excess EBs (marked by *GBE-Su(H)-lacZ*, magenta) are readily detected in midguts of newly eclosed *Apc1* mutants (C). In contrast, this defect is not observed in age-matched *ebd1* mutants (B) and is suppressed in *ebd1 Apc1* double mutants (D). Nuclei are labeled by DAPI (turquoise).
(E) Quantification of *GBE-Su(H)-lacZ* positive cells in newly eclosed control, *ebd1*, *Apc1* and *ebd1 Apc1* mutants. **** P<0.0001, *** P<0.001, ns: not significant (t-test).
Scale bars: (A-D) 10 μm.
Genotypes: **control:** *GBE-Su(H)-lacZ/+*
***ebd1*:** *GBE-Su(H)-lacZ/+; ebd1*^*240*^
***Apc1*:** *GBE-Su(H)-lacZ/+; Apc1*^*Q8*^
***ebd1 Apc1*:** *GBE-Su(H)-lacZ; ebd1*^*240*^ *Apc1*^*Q8*^.
(TIF)
