# Supplementary figures and images for: Intestinal stem cell overproliferation resulting from inactivation of the APC tumor suppressor requires the transcription cofactors Earthbound and Erect wing
Source: PLoS Genet. 2017 Jul 14;13(7):e1006870. doi: 10.1371/journal.pgen.1006870 (PMC5510812; doi:10.1371/journal.pgen.1006870)

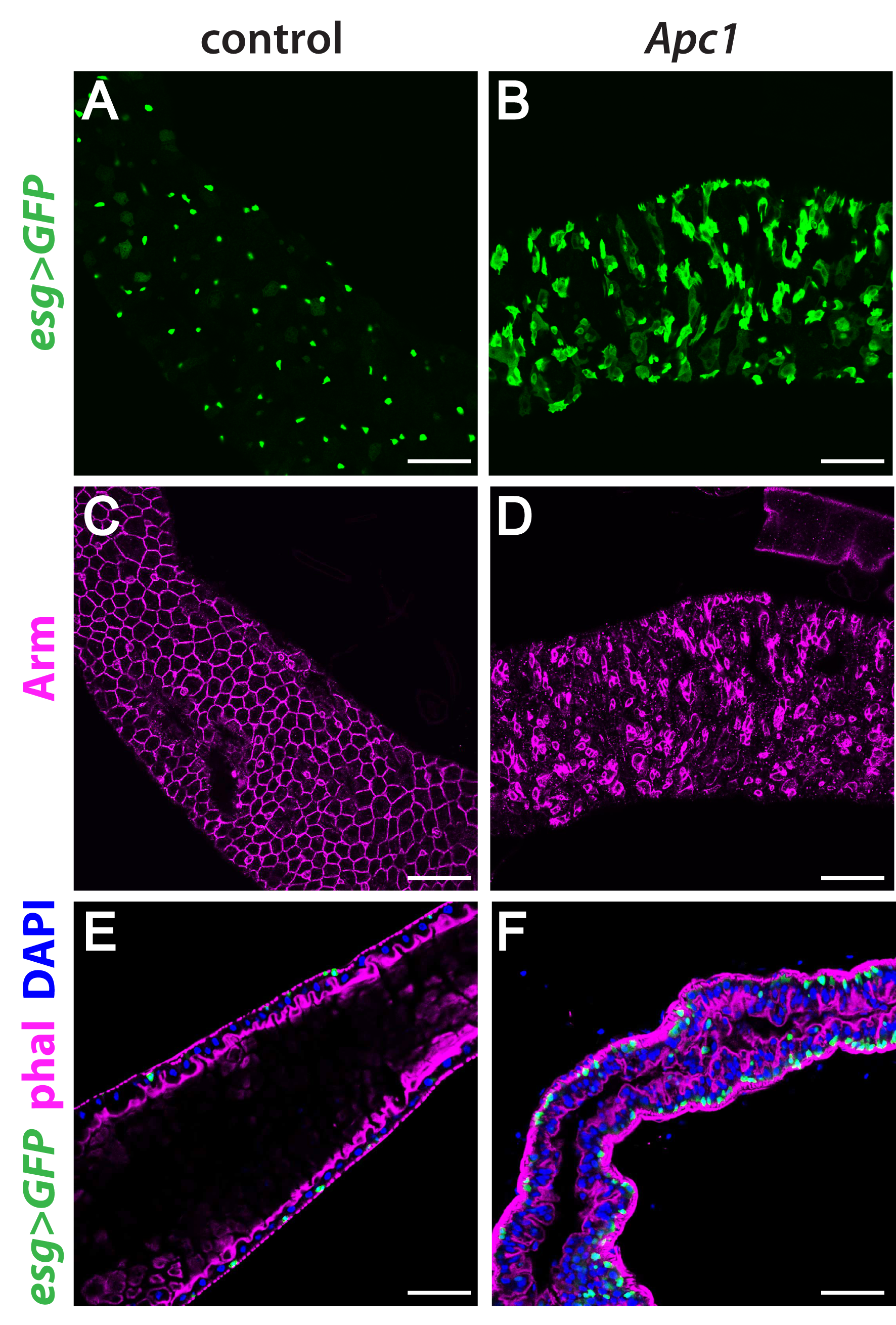

Supplement: S1 Fig — (A-B) Dramatically increased numbers of esg>GFP marked progenitor cells are detected in Apc1 mutants. (C-D) Arm localization, which defines cell-cell junctions in the intestinal epithelium, is severely altered in Apc1 mutants: the small progenitor cells form chains and clusters with strong Arm staining, whereas the large ECs have much less membrane-associated Arm. (E-F) By contrast to the monolayer intestinal architecture in controls, Apc1 mutant guts display extensive multi-layering and epithelial hyperplasia (cross-sectional view). Scale bars: 50 μm. Genotypes: control: esg-Gal4 UAS-GFP/+; Apc1Q8/+ Apc1: esg-Gal4 UAS-GFP/+; Apc1Q8. (TIF) [file pgen.1006870.s001.tif]

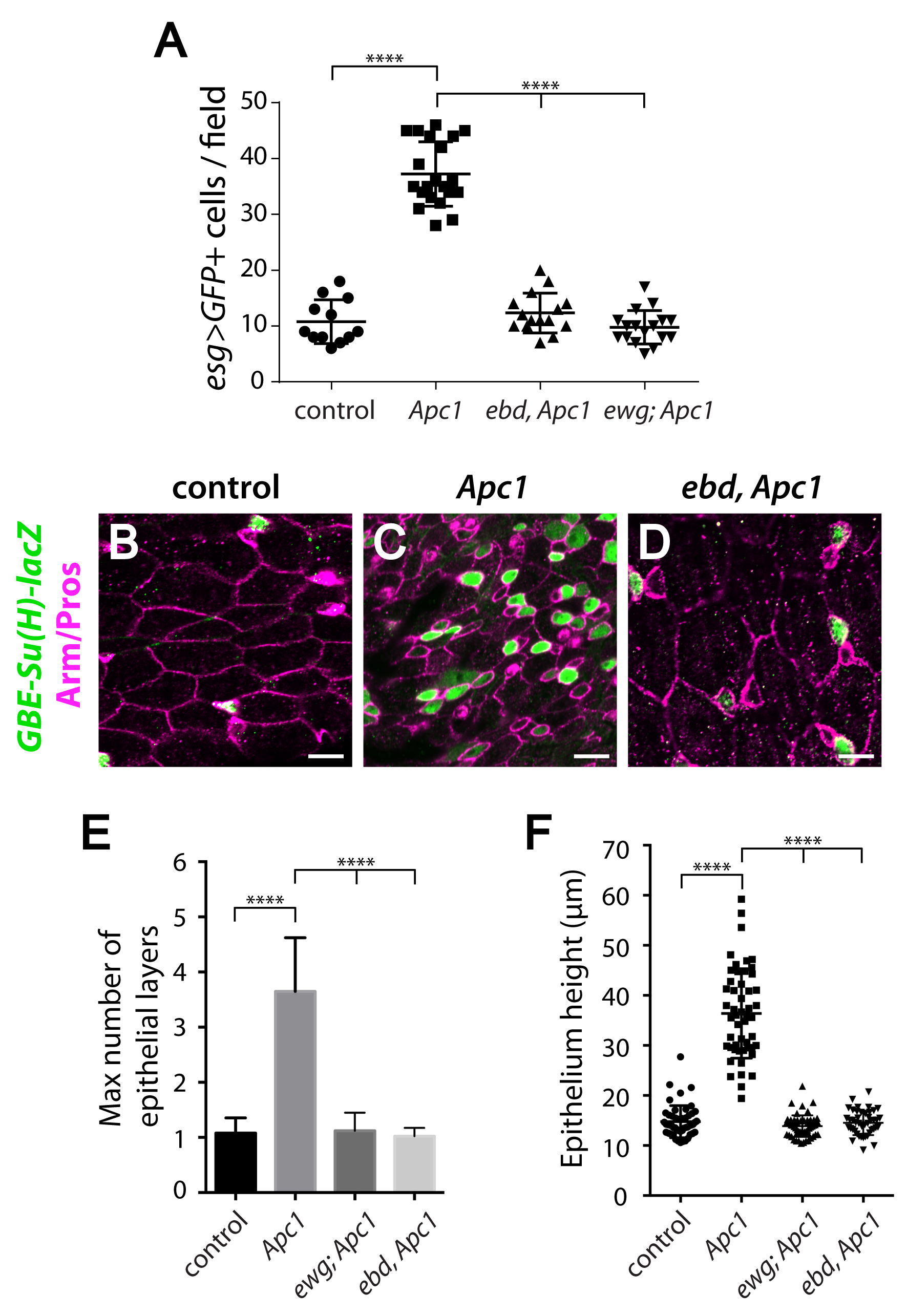

Supplement: S2 Fig — (A) Quantification of esg>GFP positive progenitor cells in control, Apc1 mutants, ebd Apc1 mutants and ewg Apc1 mutants. Increased number of progenitor cells in Apc1 mutants is rescued by concomitant loss of ebd or ewg. **** P<0.0001 (Mann-Whitney test). (B-D) Inactivation of Apc1 leads to dramatically increased numbers of EBs, marked with GBE-Su(H)-lacZ (green; compare C to B). This defect is suppressed in ebd Apc1 double mutants (D). (E) In contrast to the control guts, which exhibit a monolayer intestinal architecture, the Apc1 mutant guts are multi-layered. This defect is rescued in ebd Apc1 or ewg Apc1 double mutants. **** P<0.0001 (t-test). (F) Compared to controls, the maximum epithelial height is greatly increased in Apc1 mutants. The height is reverts to a normal level upon concomitant loss of ebd or ewg. **** P<0.0001 (t-test). Scale bars: (B-D) 10 μm. Genotypes: (A) control: esg-Gal4 UAS-GFP; Apc1Q8/+ Apc1: esg-Gal4 UAS-GFP; Apc1Q8 ebd Apc1: esg-Gal4 UAS-GFP; ebd1240 Apc1Q8/Df(3L)9698 ebd2136 Apc1Q8 ewg Apc1: ewgP1; esg-Gal4 UAS-GFP; Apc1Q8 (B-D) control: GBE-Su(H)-lacZ/+; Apc1Q8/+ Apc1: GBE-Su(H)-lacZ/+; Apc1Q8 ebd Apc1: GBE-Su(H)-lacZ/+; ebd1240 Apc1Q8/Df(3L)9698 ebd2136 Apc1Q8 (E-F) control: Apc1Q8/+ Apc1: Apc1Q8 ebd Apc1: ebd1240 Apc1Q8 ewg Apc1: ewgP1; Apc1Q8. (TIF) [file pgen.1006870.s002.tif]

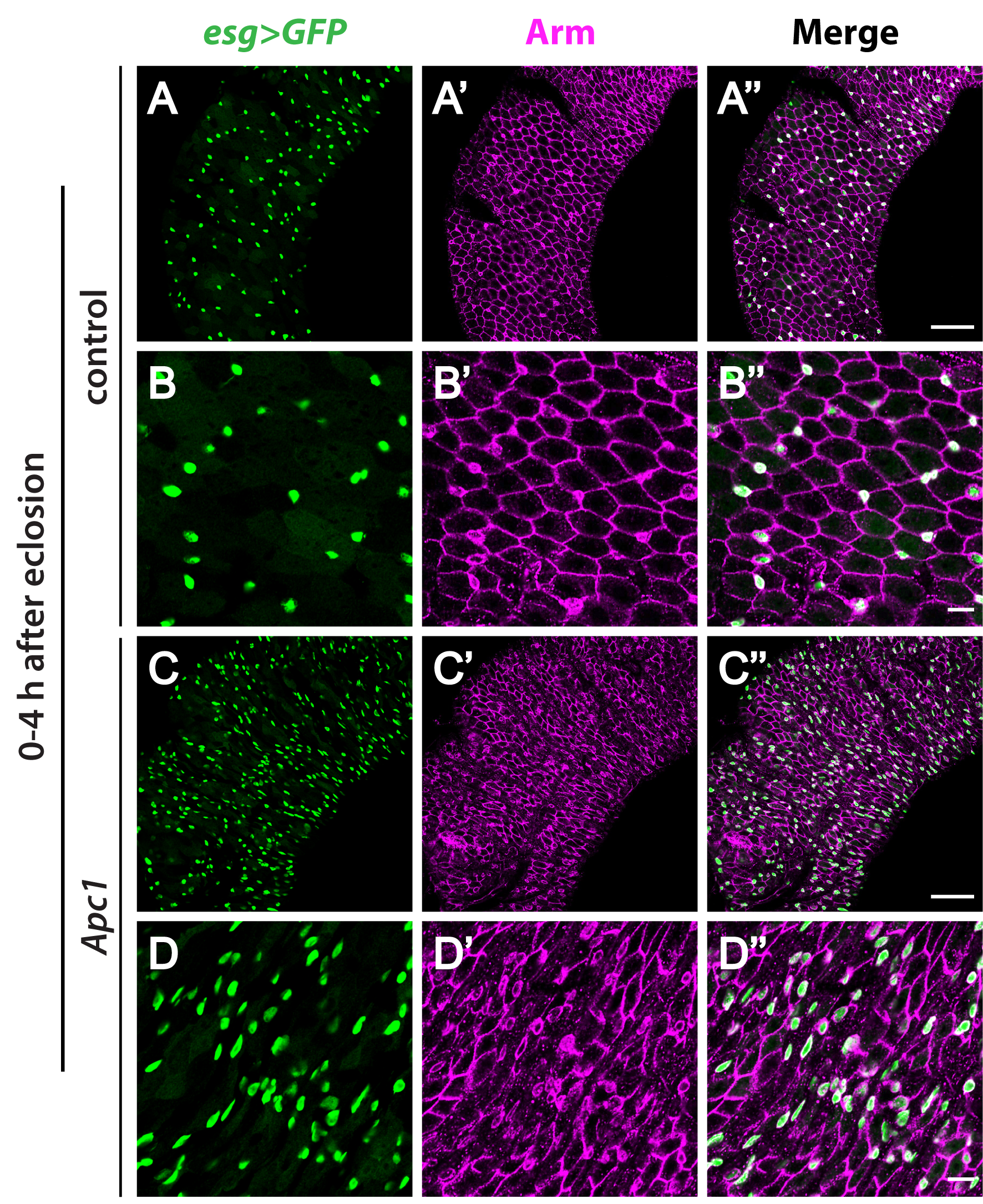

Supplement: S3 Fig — Excess numbers of progenitor cells, marked by esg>GFP (green), are readily detected in newly eclosed Apc1 mutant guts (compare C-C” to A-A”; high magnification view: compare D-D” to B-B”). The cell-cell junctions, which are marked by Arm (magenta), remained largely intact at this stage (compare C’ to A’; high magnification view: compare D’ to B’). Scale bars: (A-A” and C-C”) 50 μm, (B-B” and D-D”) 10 μm. Genotypes: control: esg-Gal4 UAS-GFP; Apc1Q8/+ Apc1: esg-Gal4 UAS-GFP; Apc1Q8. (TIF) [file pgen.1006870.s003.tif]

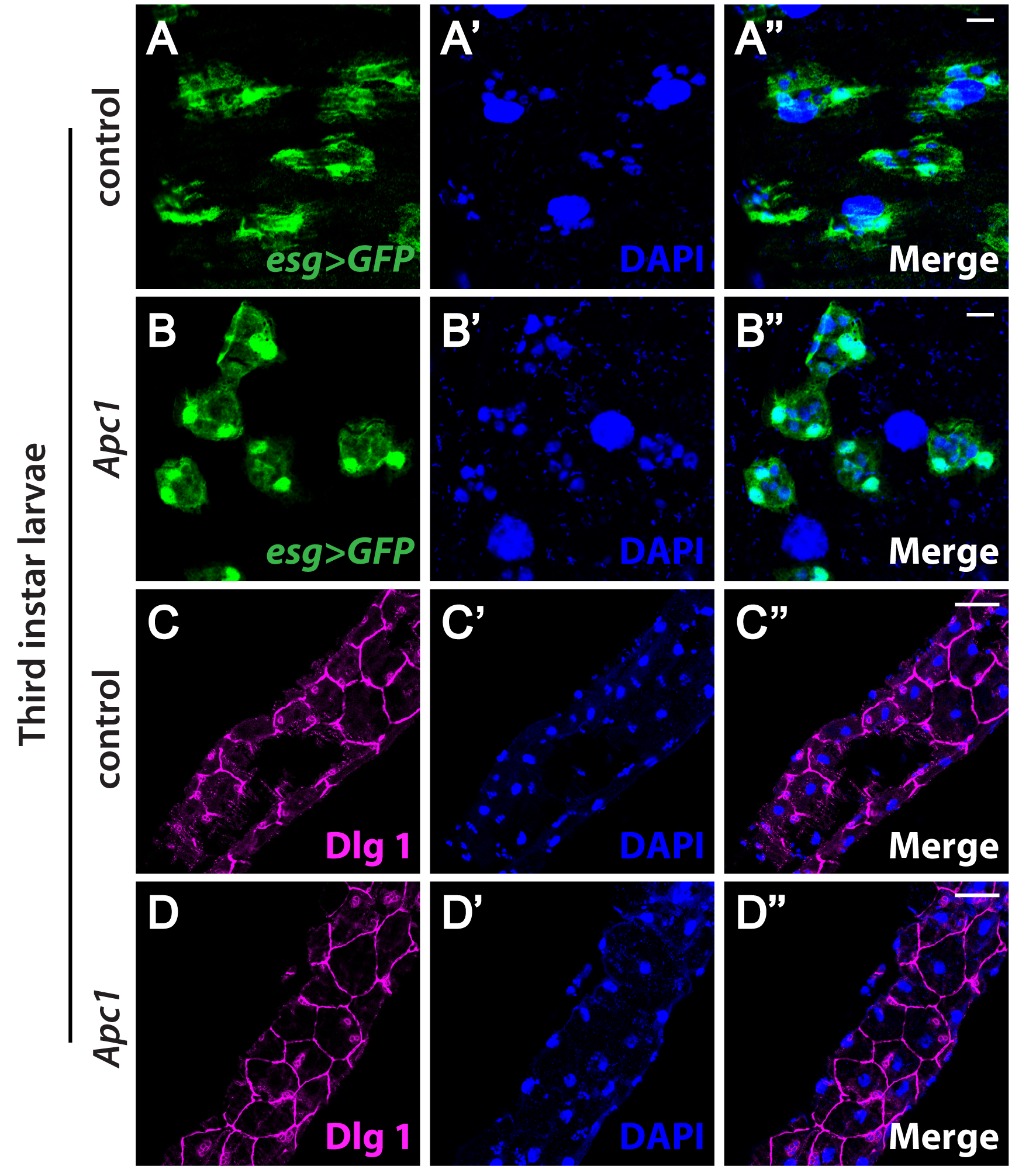

Supplement: S4 Fig — (A-B”) Numbers of AMPs (adult midgut progenitors), marked by esg>GFP (green), are comparable between control and Apc1 mutant guts. (C-D”) The cell-cell junctions, marked by membrane-associated Discs large 1 (Dlg1, magenta), remain intact at this stage. Scale bars: (A-B”) 10 μm, (C-D”) 50 μm. Genotypes: control: esg-Gal4 UAS-GFP; Apc1Q8/+ Apc1: esg-Gal4 UAS-GFP; Apc1Q8. (TIF) [file pgen.1006870.s004.tif]

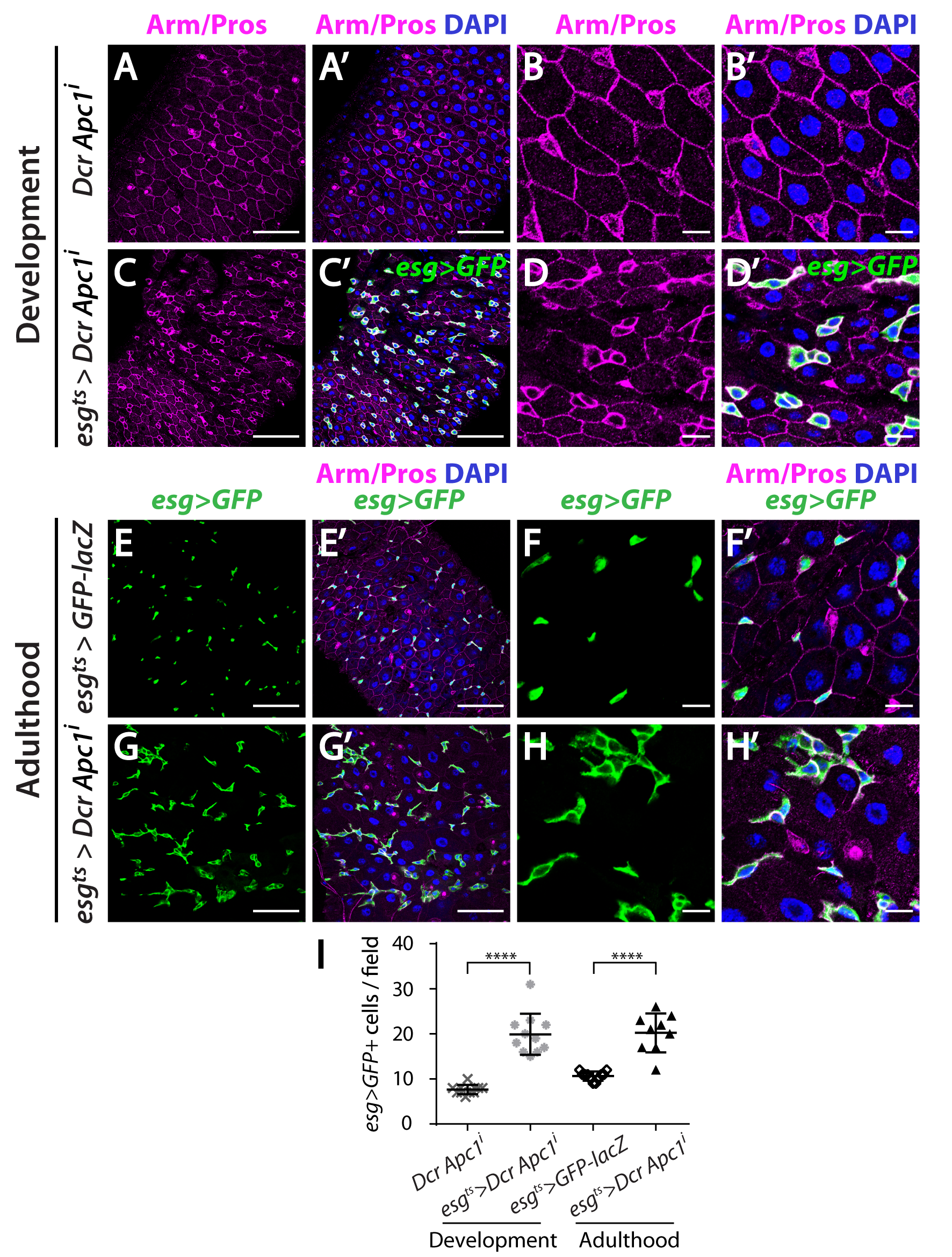

Supplement: S5 Fig — (A-D”) Apc1 expression, knocked down using the esgts driver during formation of the adult gut (crosses were shifted from 18°C to 29°C during second instar larval stage and the progeny of desired genotype were examined 2–3 days post-eclosion), results in excess progenitor cells. Progenitor cells are identified as small cells with strong Arm staining and lack of Prospero staining (magenta) or by esg>GFP (green). Nuclei are labeled with DAPI (blue). Low magnification view: A-A’ (control) and C-C’ (Apc1 RNAi); high magnification view: B-B’ (control) and D-D’ (Apc1 RNAi). (E-F”) Apc1 expression, knocked down using the esgts driver during adulthood (progeny of desired genotype were shifted from 18°C to 29°C after eclosion and analyzed 14 days later), also results in excess progenitor cells (marked by esg>GFP, green). Nuclei are labeled with DAPI (blue). Low magnification view: E-E’ (control) and G-G’ (Apc1 RNAi); high magnification view: F-F’ (control) and H-H’ (Apc1 RNAi). (I) Quantification of progenitor cell numbers when Apc1 expression is knocked down during formation of the adult gut or during adulthood reveals dramatic increases in both contexts. **** P<0.0001 (t-test). Scale bars: (A-A’, C-C’, E-E’ and G-G’) 50 μm, (B-B’, D-D’, F-F’ and H-H’) 10 μm. Genotypes: Dcr Apc1i1: UAS-Dicer2/+; UAS-Apc1 RNAi#1/+ esgts>Dcr Apc1i1: UAS-Dicer2/+; esg-Gal4 tubGal80ts UAS-GFP/+; UAS-Apc1 RNAi#1/+ esgts>GFP-lacZ: esg-Gal4 tubGal80ts UAS-GFP/+; UAS-GFP-lacZ. (TIF) [file pgen.1006870.s005.tif]

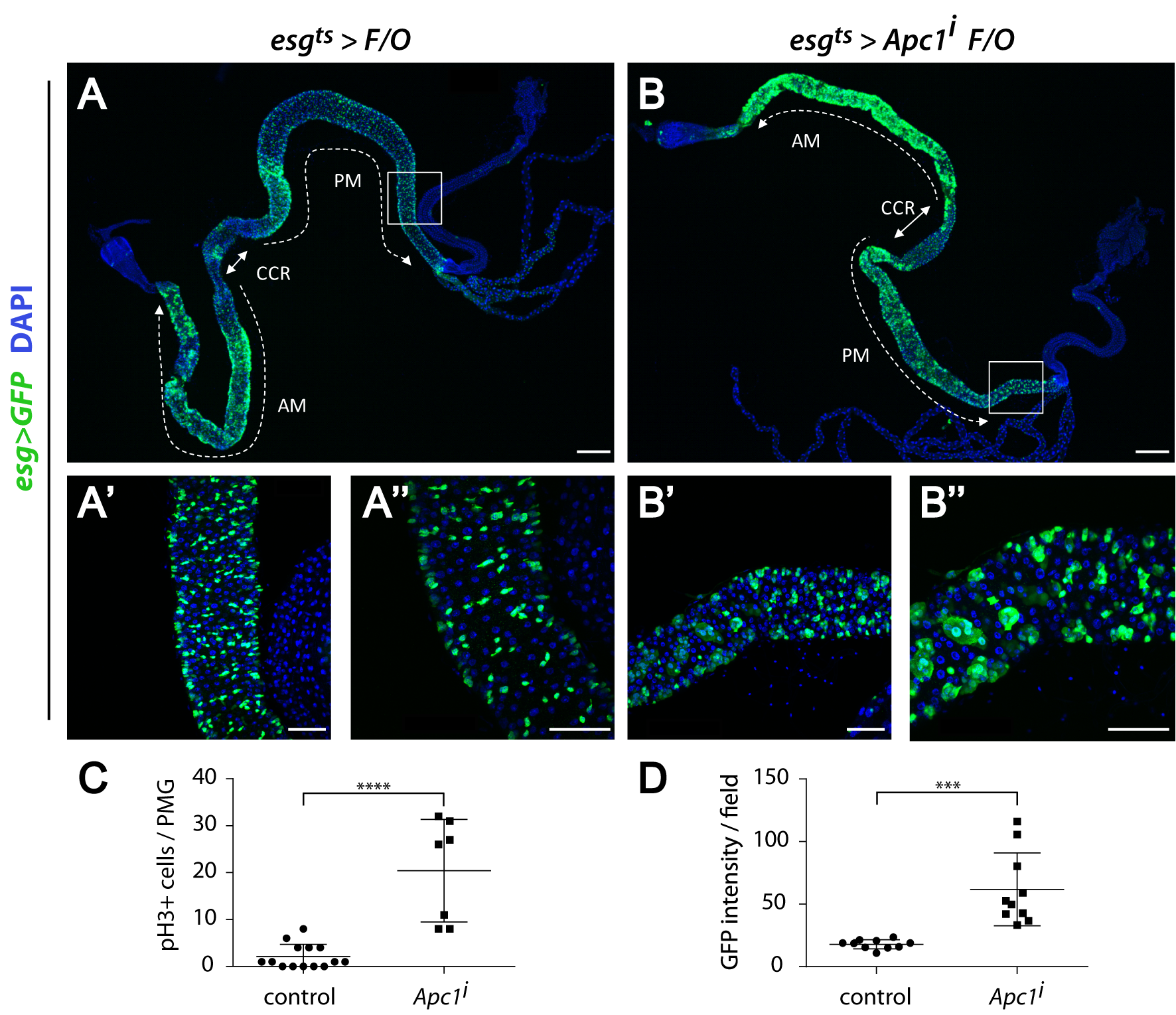

Supplement: S6 Fig — (A-B”) Knockdown of Apc1 expression during adulthood (2–3 day old adults of the desired genotypes were shifted from 18°C to 29°C for 14 days before analysis) results in increased stem/progenitor cell self-renewal (marked by GFP, green) using the stem/progenitor and lineage tracing “esgts flip out” system. Nuclei are labeled with DAPI (blue). PM: posterior midgut; AM: anterior midgut; CCR: copper cell region. (C) Quantification of ISC proliferation by pH3 scoring upon Apc1 knockdown during adulthood. PMG: posterior midgut. **** P<0.001 (t-test). Number of guts (n): control guts: n = 14 and Apc1i1, Apc1i2 guts: n = 7. (D) Measurement of total GFP area in posterior midguts (PM) of control and adult-specific Apc1 RNAi driven by the “esgts flip out”system”. *** P<0.001 (t-test). For both conditions, 2 pictures in different regions of the posterior midgut were taken for each midgut, n = 5. Scale bars: (A and B) 100 μm, (A’-A”) and (B’-B”) 50 μm. Genotypes: esgts>F/O: esg-Gal4, tub-Gal80ts, UAS-GFP/+; UAS-flp Act>CD2>Gal4, UAS-GFP/+ esgts>UAS- Apc1i F/O: esg-Gal4, tub-Gal80ts, UAS-GFP/ UAS-Apc1 RNAi#2; UAS-flp Act>CD2>Gal4, UAS-GFP / UAS-Apc1 RNAi#1. (TIF) [file pgen.1006870.s006.tif]

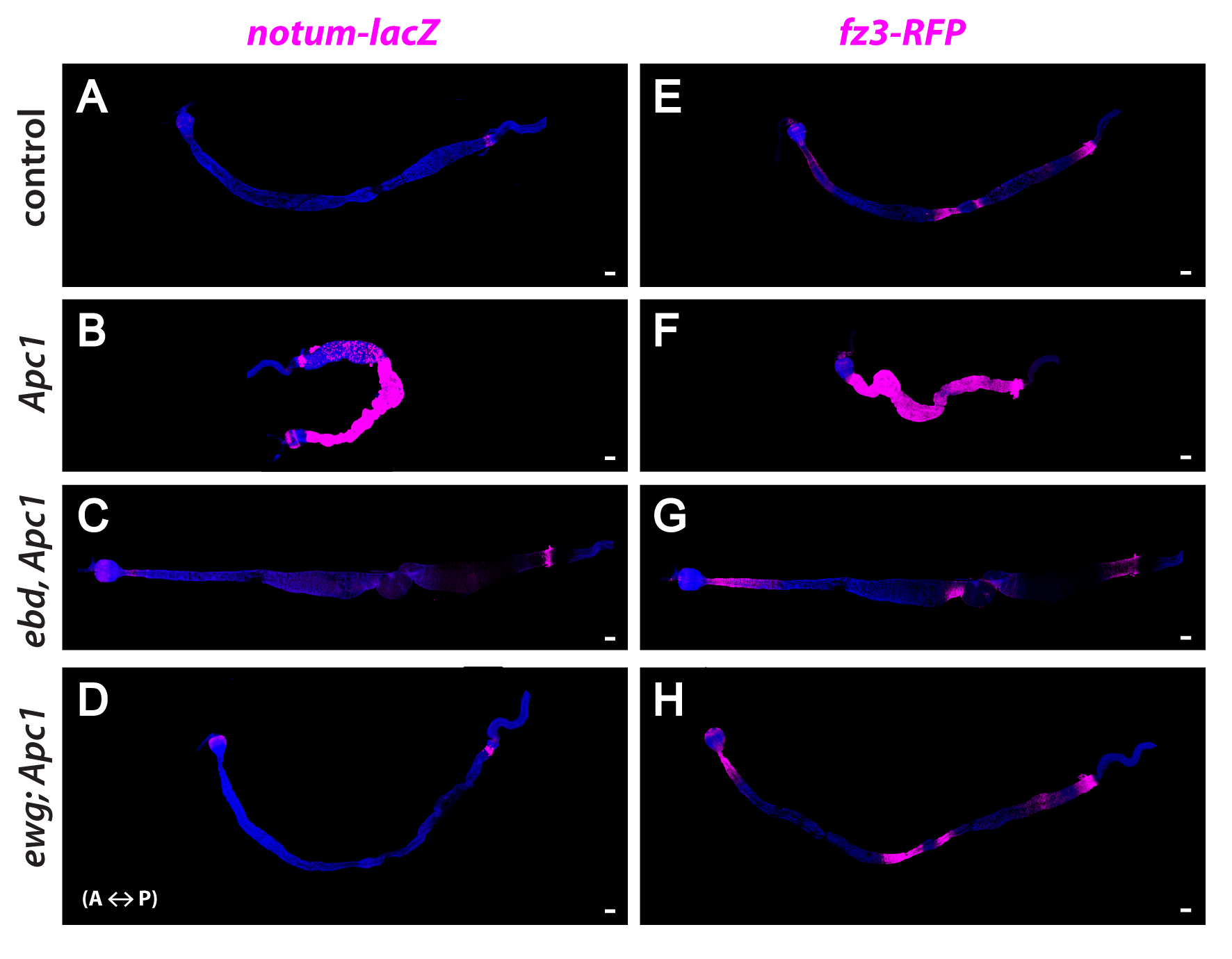

Supplement: S7 Fig — Expansion of notum-lacZ (magenta; A and B) and fz3-RFP (magenta; E and F) expression upon loss of Apc1 is suppressed by inactivation of ebd (C and G) or ewg (D and H). Anterior to the left. Scale bars: 100 μm. Genotypes: (A-D) control: notum-lacZ/+; Apc1Q8/+ Apc1: notum-lacZ/+; Apc1Q8 ebd Apc1: notum-lacZ/fz3-RFP; ebd1240 Apc1Q8/Df(3L)9698 ebd2136 Apc1Q8 ewg Apc1: ewgP1; notum-lacZ/+; Apc1Q8 (E-H) control: fz3-RFP/+; Apc1Q8/+ Apc1: fz3-RFP/+; Apc1Q8 ebd Apc1: notum-lacZ/fz3-RFP; ebd1240 Apc1Q8/Df(3L)9698 ebd2136 Apc1Q8 (same gut as C) ewg Apc1: ewgP1; fz3-RFP/+; Apc1Q8. (TIF) [file pgen.1006870.s007.tif]

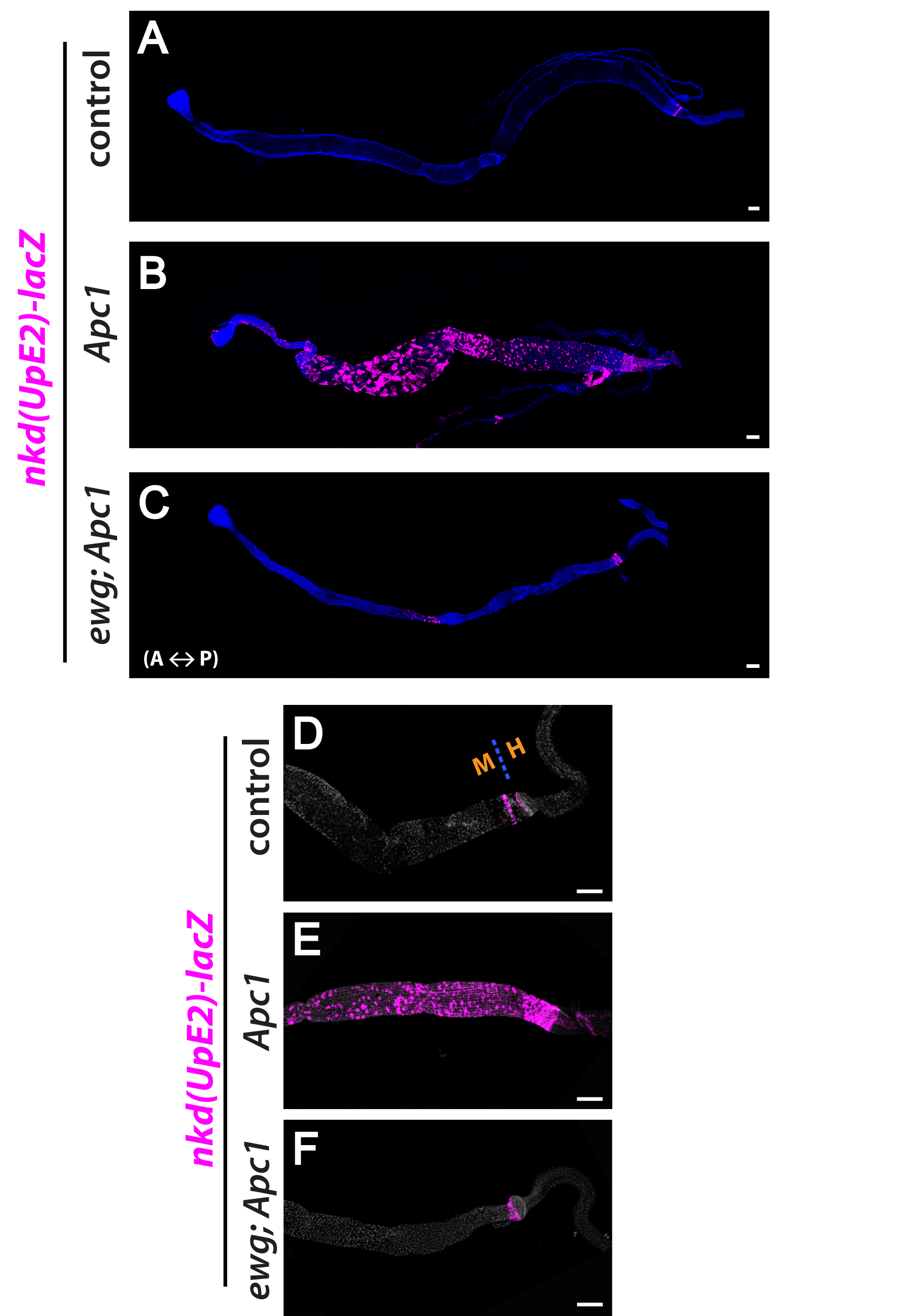

Supplement: S8 Fig — Expansion of nkd(UpE2)-lacZ expression [magenta, compare B to A (low magnification view) and E to D (high magnification view)] upon loss of Apc1 is suppressed by further inactivation of ewg (C and F). Nuclei are marked with DAPI. Anterior to the left. Scale bars: 100 μm. Genotypes: control: nkd(UpE2)-lacZ/+; Apc1Q8/+ Apc1: nkd(UpE2)-lacZ/+; Apc1Q8 ewg Apc1: ewgP1; nkd(UpE2)-lacZ/+; Apc1Q8. (TIF) [file pgen.1006870.s008.tif]

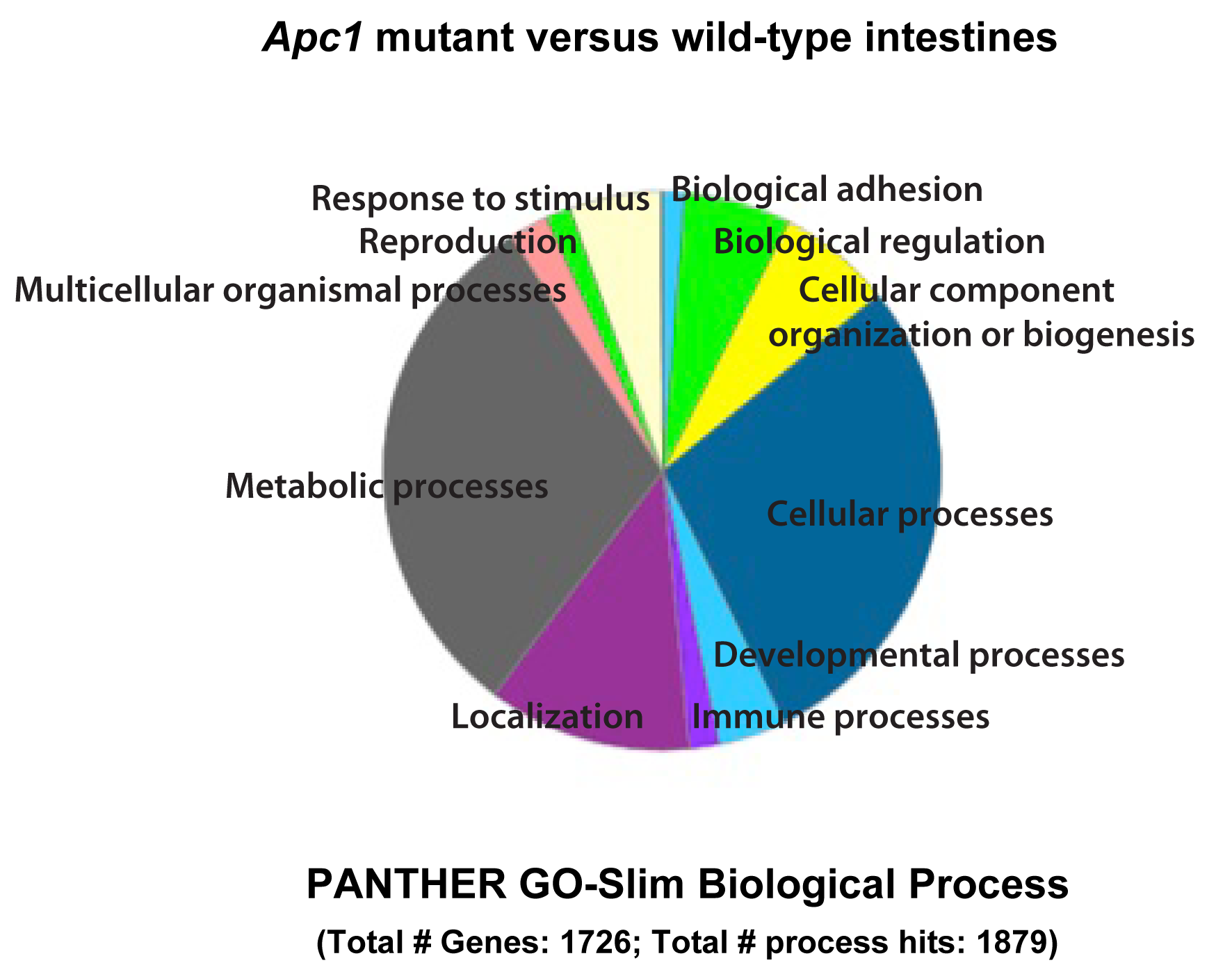

Supplement: S9 Fig — A GO term analysis was performed on biological processes deregulated in Apc1 mutant guts when compared to the wild type controls and the top enriched GO terms (p-value is p<0.05 for genes showing a minimum of 1.5 fold change) are visualized. (TIF) [file pgen.1006870.s009.tif]

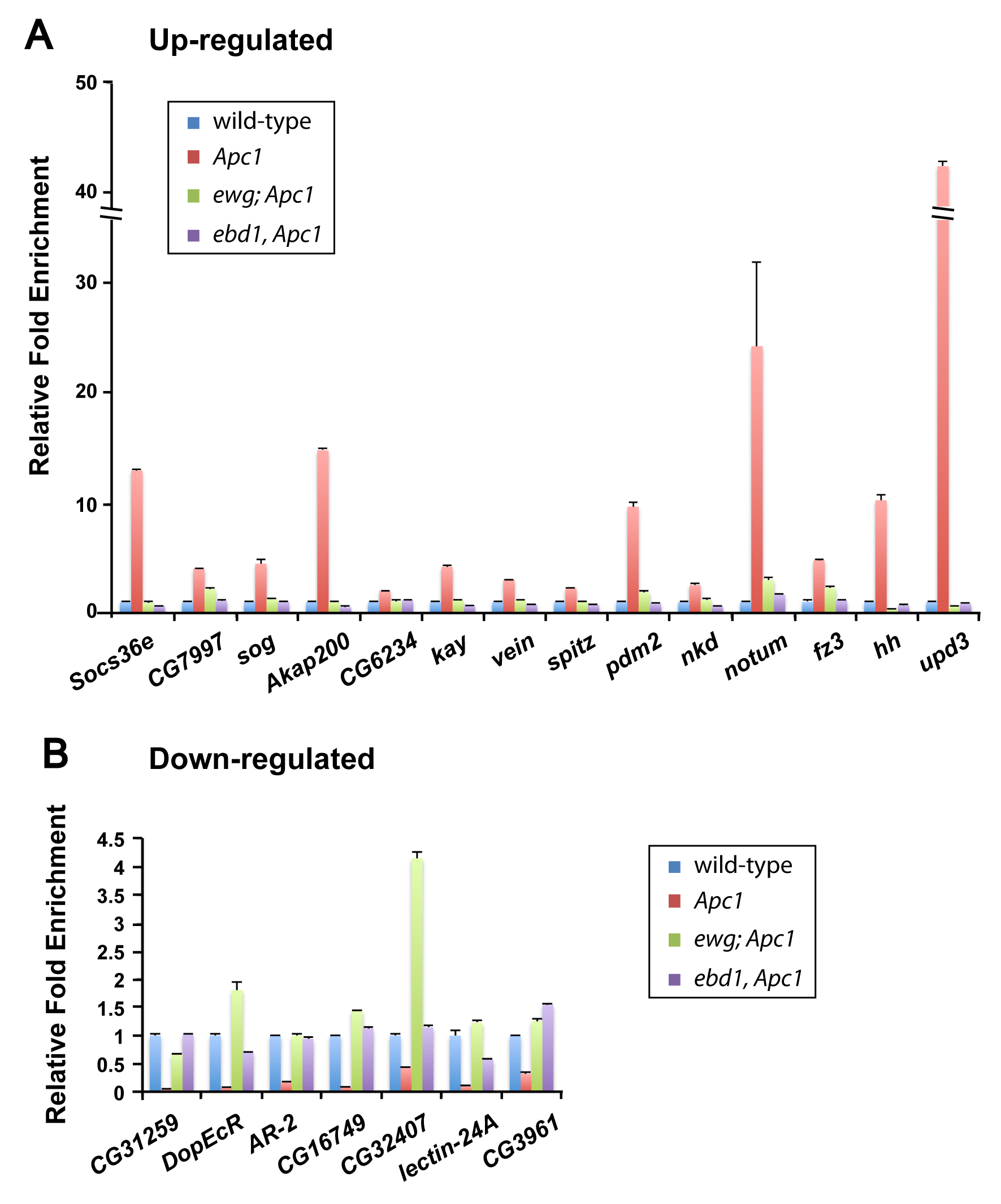

Supplement: S10 Fig — Quantitative RT-PCR of genes up- (A) or down-regulated (B) by loss of Apc1. Misexpression of both sets of genes is rescued in ebd1 Apc1 and ewg Apc1 double mutants. This is an independent biological replicate of the data shown in Fig 3. Genotypes: control: Apc1Q8/+ Apc1: Apc1Q8 ebd Apc1: ebd1240 Apc1Q8 ewg Apc1: ewgP1; Apc1Q8. (TIF) [file pgen.1006870.s010.tif]

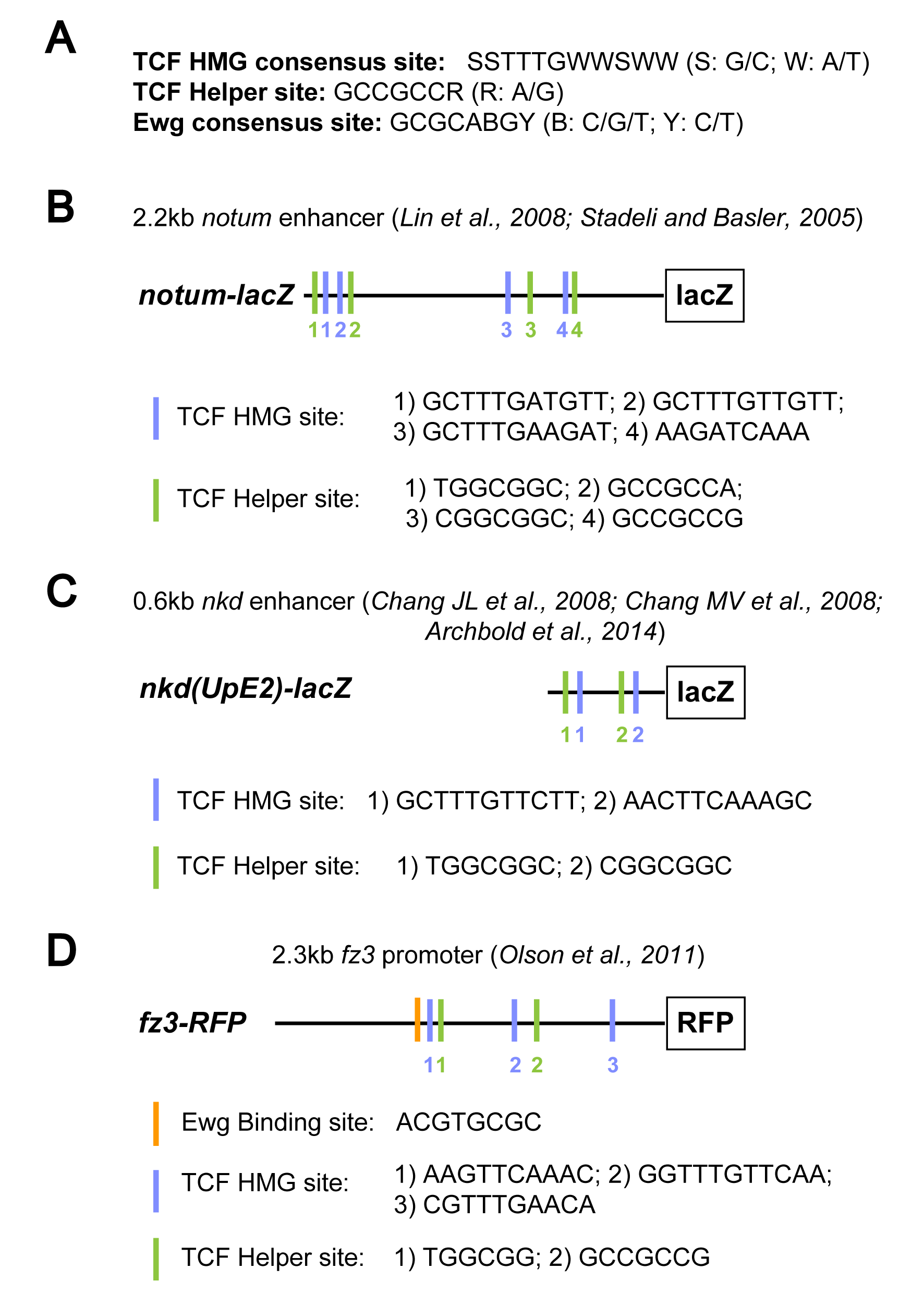

Supplement: S11 Fig — (A) Sequence of TCF core (HMG: High Mobility Group) consensus DNA binding sites, TCF Helper site, as well as Ewg consensus DNA binding sites. (B-D) Each of the Wingless reporters contains at least one TCF HMG consensus binding site and TCF Helper site, but only fz3-RFP (D) has an Ewg consensus binding site. (TIF) [file pgen.1006870.s011.tif]

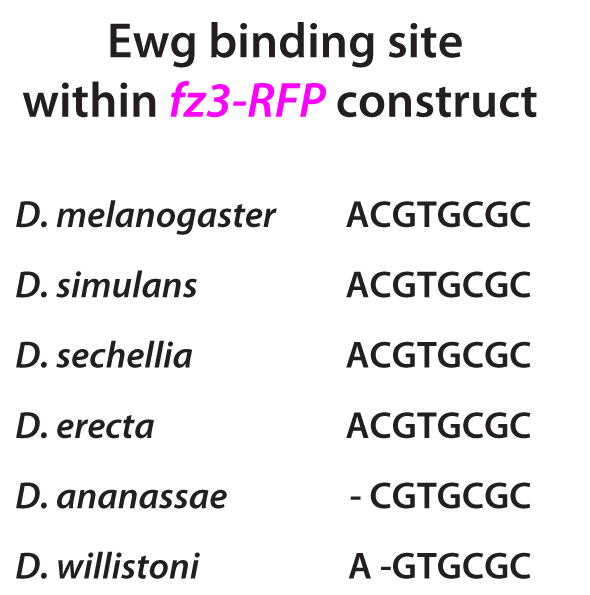

Supplement: S12 Fig — Using an Evoprinter analysis to identify site conservation, we found that the Ewg consensus binding site within the fz3-RFP reporter is 100% conserved within the melanogaster group (except that it is absent in the Drosophila yakuba species), and with only one substitution in the evolutionarily distant Drosophila ananassae and Drosophila willistoni species. (TIF) [file pgen.1006870.s012.tif]

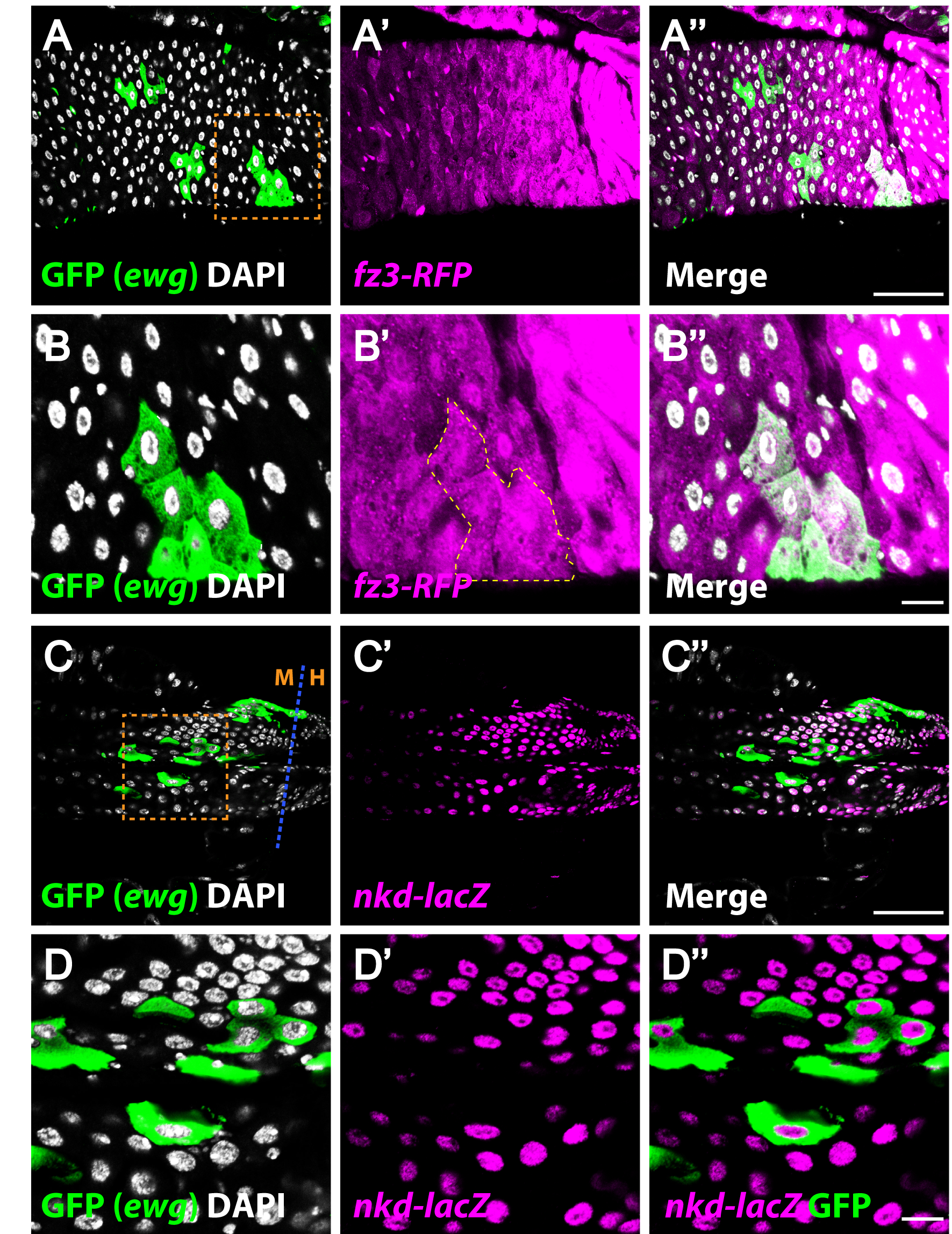

Supplement: S13 Fig — Expression of fz3-RFP (A-B”) or nkd-lacZ (C-D”), reporters for Wingless signaling (in magenta), is retained in ewg null mutant clones, suggesting that Ewg is not required for Wingless-dependent expression of fz3 or nkd. Clones are marked with GFP (green). Magnified views of the boxed regions in (A or C) are shown in (B-B” and D-D”), respectively. Scale bars: (A and C) 50 μm and (B-B” and D-D”) 10 μm. Genotypes: (A-B”) hs-flp tub-Gal80 FRT19A/ewg2 FRT19A; fz3-RFP/+; tub-Gal4 UAS-mCD8::GFP/+ (C-D”) hs-flp tub-Gal80 FRT19A/ewg2 FRT19A; tub-Gal4 UAS-mCD8::GFP/nkd-lacZ. (TIF) [file pgen.1006870.s013.tif]

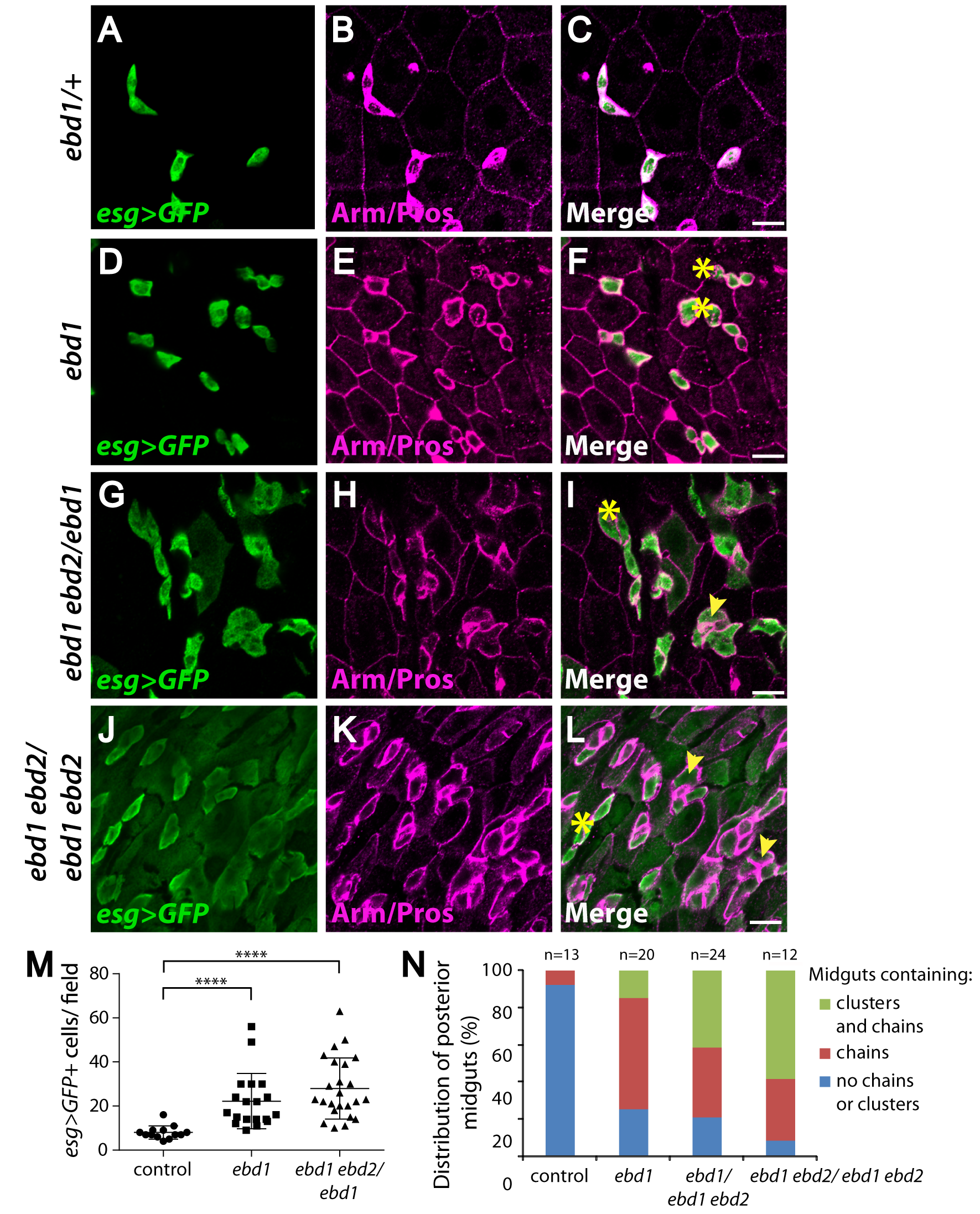

Supplement: S14 Fig — (A-C) Progenitor cells, marked with esg>GFP (green, A) and observed as small cells with high levels of membrane-associated Arm and the absence of Prospero (Pros) staining (magenta, B), are either present as single cells or doublets distributed evenly in ebd1/+ control guts. (D-F) Chains of small esg>GFP positive progenitor cells (asterisk in F), with strong Arm staining, are observed in ebd1240 mutants. (G-I) Flies homozygous mutant for ebd1 and heterozygous mutant for ebd2 (ebd15 ebd2136/ ebd1240) contain multi-cell clusters of esg>GFP positive cells (arrowhead in I), as well as chains of progenitor cells (asterisk). (J-L) The prominence of clusters of esg>GFP positive cells is increased in flies homozygous mutant for both ebd1 and ebd2 (ebd15 ebd2136/ ebd1240 ebd2136). (M) ebd1 and ebd1 ebd2/ebd1 mutants have higher numbers of esg>GFP positive progenitor cells compared to controls. **** P<0.0001 (Mann-Whitney test). (N) Proportion of posterior midguts containing chains and clusters of esg>GFP positive cells. Scale bars: (A-L) 10 μm. Genotypes: ebd1/+: esg-Gal4 UAS-GFP; ebd1240/+ ebd1: esg-Gal4 UAS-GFP; ebd1240 ebd1ebd2/ebd1: esg-Gal4 UAS-GFP; ebd15 ebd2136/ ebd1240 ebd1ebd2: esg-Gal4 UAS-GFP; ebd15 ebd2136/ ebd1240 ebd2136. (TIF) [file pgen.1006870.s014.tif]

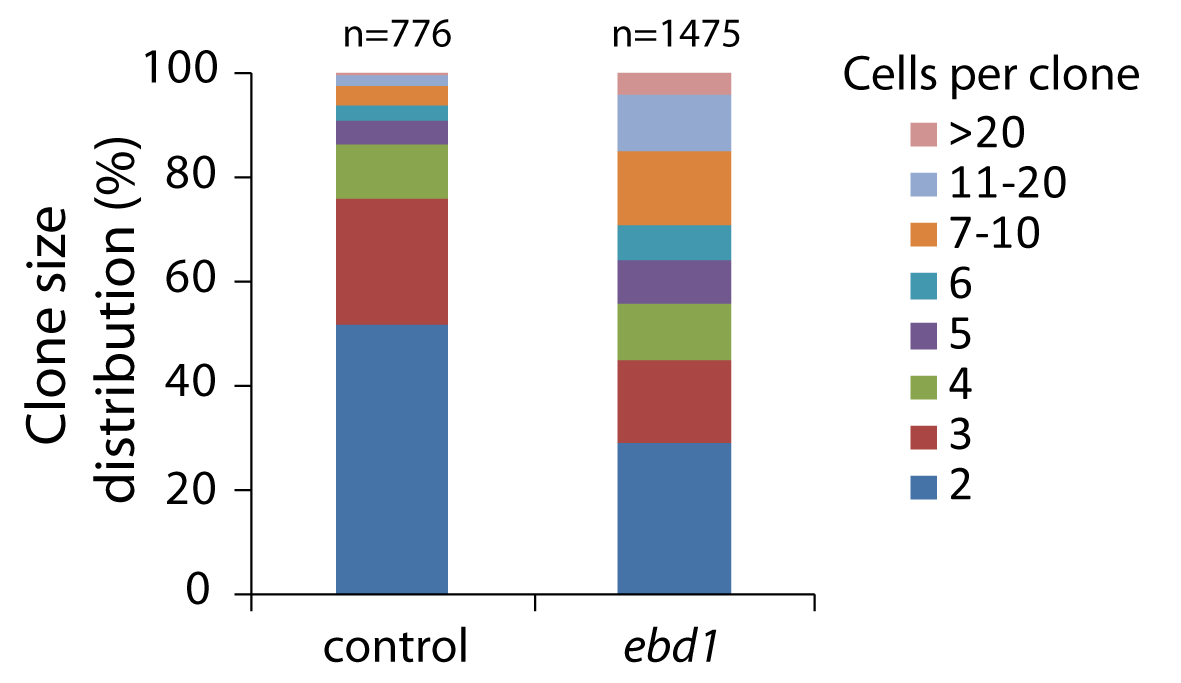

Supplement: S15 Fig — Adult ebd1 clones are larger than control clones, indicating that Ebd1 is required for homeostasis of intestinal tissue during adulthood. Transient clones were excluded by restricting the analysis to clones of two or more cells. Genotypes: control: y w hs-flp/+; tub-Gal4 UAS-GFP+; FRT2A tub-Gal80/FRT2A ebd1: y w hs-flp/+; tub-Gal4 UAS-GFP/+; FRT2A tub-Gal80/ebd1240 FRT2A. (TIF) [file pgen.1006870.s015.tif]

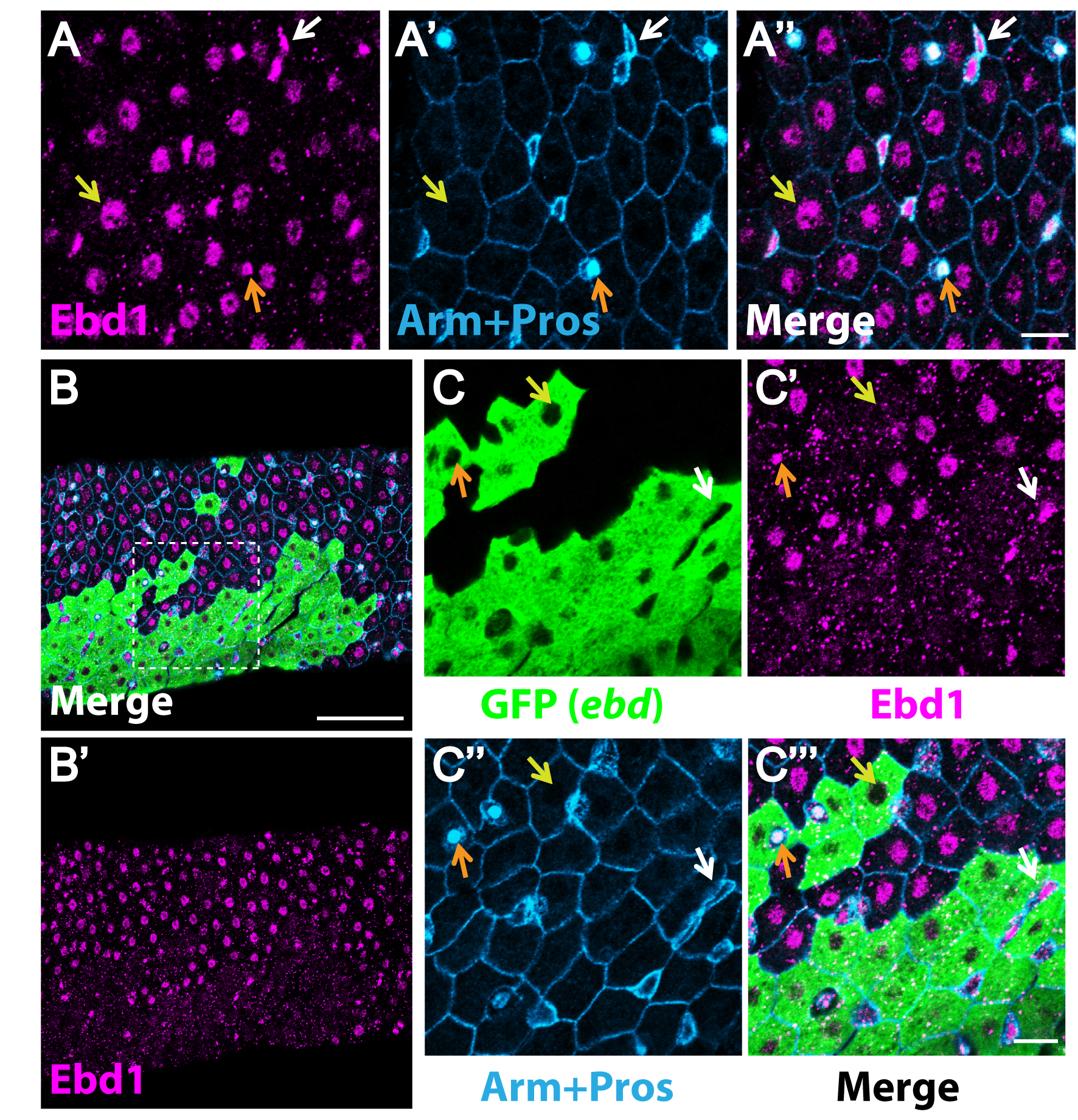

Supplement: S16 Fig — (A-A”) Fixed intestines were immunostained with the Ebd1 antibody and signals are detected in all intestinal epithelial cell types, including ECs (yellow arrow), progenitors (white arrow) and EEs (orange arrow). (B-C”‘) Ebd1 staining is specifically diminished inside enterocytes in ebd1 null mutant clones (marked with GFP). Scale bars: (A-A”) 10 μm, (B and B’) 50 μm and (C-C”‘) 10 μm. Genotypes: (A-A”) Canton S (B-C”’) ebd1: y w hs-flp/+; tub-Gal4 UAS-GFP/+; FRT2A tub-Gal80/ebd1240 FRT2A. (TIF) [file pgen.1006870.s016.tif]

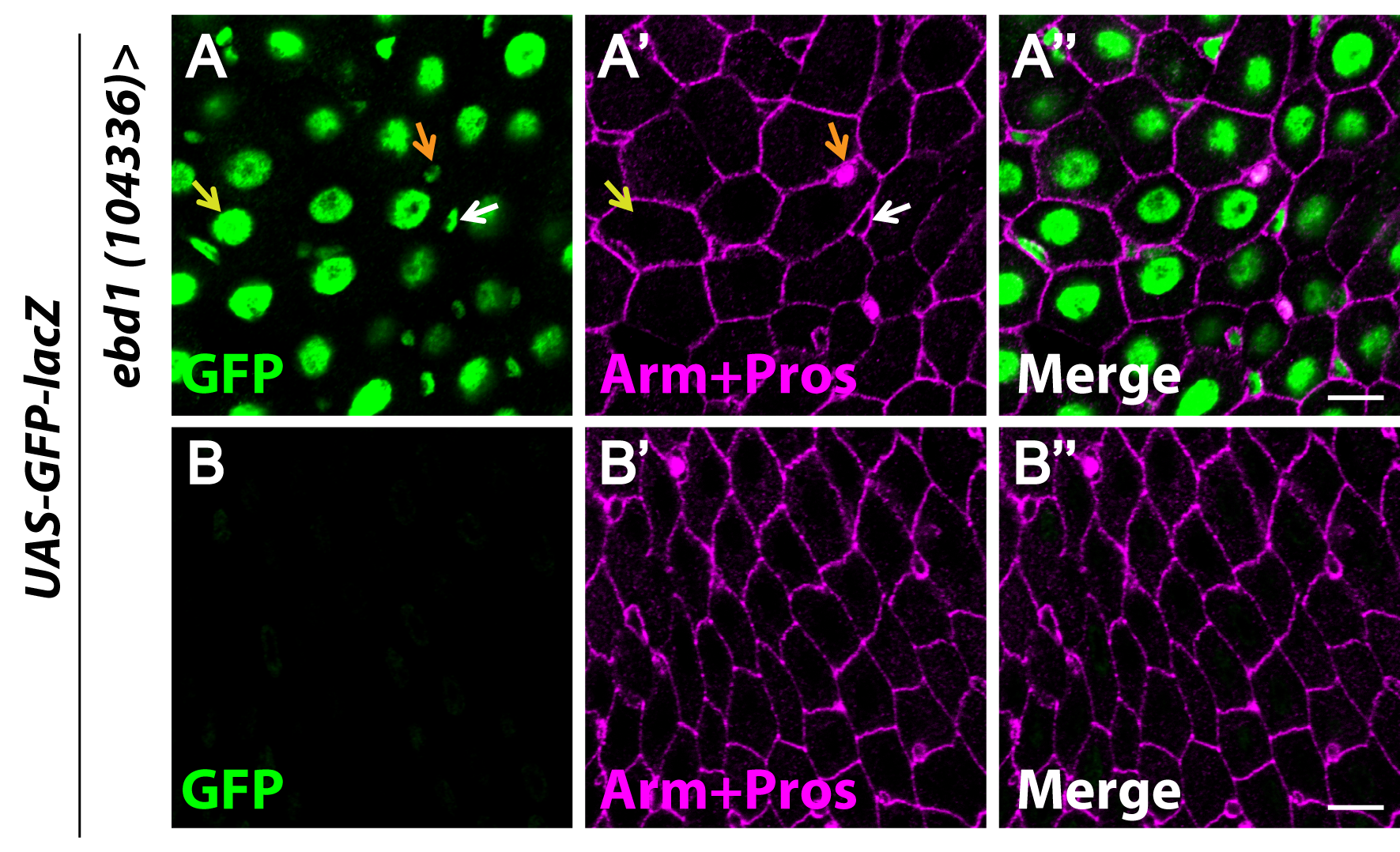

Supplement: S17 Fig — (A-A”) UAS-GFP-lacZ driven by ebd1-Gal4 (an enhancer-trap line in which Gal4 is inserted in the endogenous ebd1 locus) exhibits expression in ECs (yellow arrow), progenitors (white arrow) and EEs (orange arrow). (B-B”) No signal is detected with UAS-GFP-lacZ alone. Scale bars: 10 μm. Genotypes: (A-A”) ebd1(104336)>U-GFP-lacZ (B-B”) U-GFP-lacZ/+. (TIF) [file pgen.1006870.s017.tif]

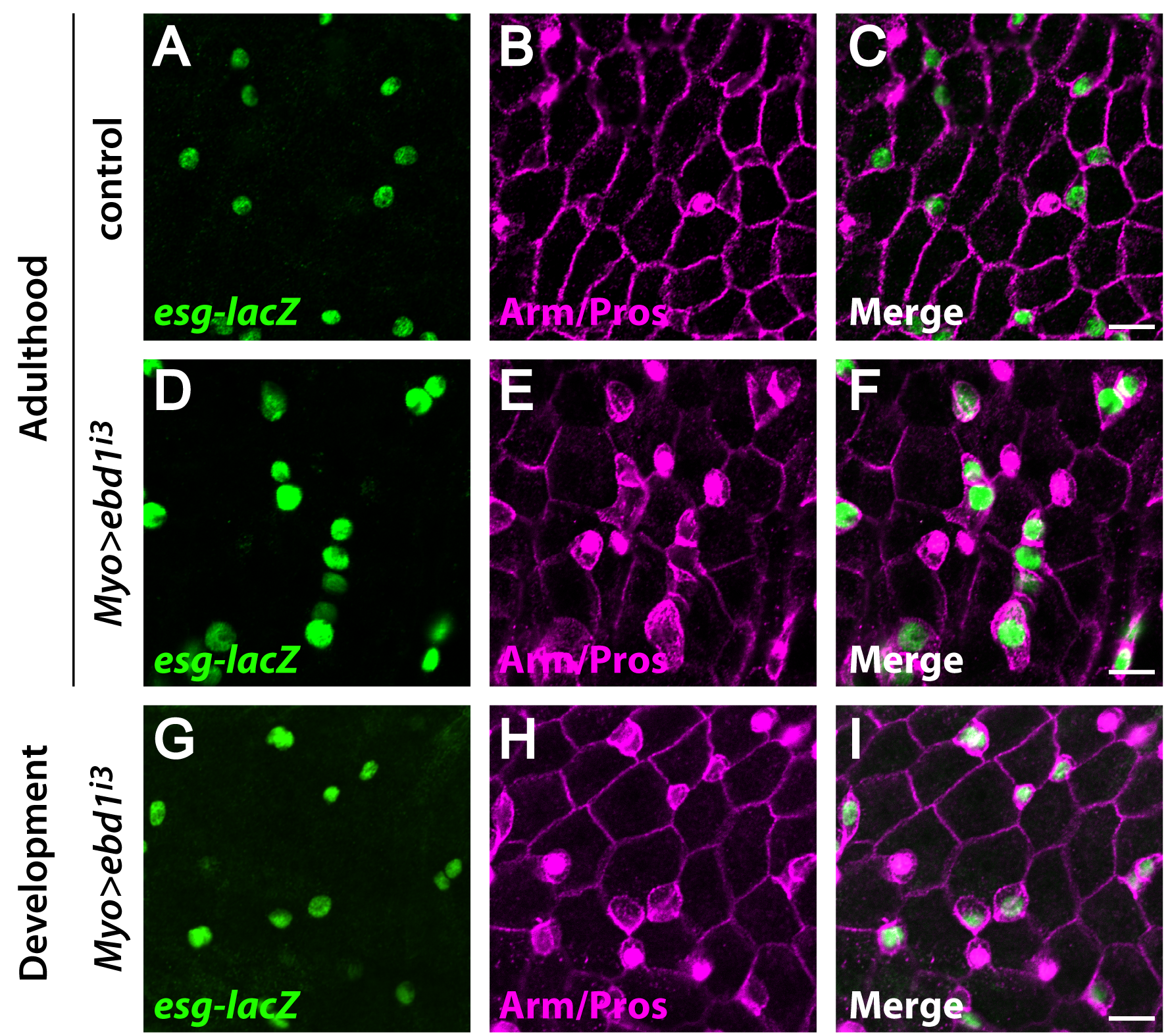

Supplement: S18 Fig — (A-F) RNAi-mediated disruption of ebd1 expression leads to increased numbers of progenitor cells (compare D-F to A-C). Progenitor cells are marked with esg-lacZ (green) or are identified as small cells with strong Arm staining and lack of Prospero (Pros) staining (magenta). Flies were analyzed seven days post-eclosion. (G-I) The number of progenitor cells in flies analyzed one day post-eclosion is not increased, indicating that Ebd1 regulates stem cell proliferation non-autonomously in adults, but not during development. Scale bars: 10 μm. Genotypes: control: Myo1A-Gal4 UAS-GFP tub-Gal80ts/esg-lacZ Myo>ebd1i3: Myo1A-Gal4 UAS-GFP tub-Gal80ts/esg-lacZ; UAS-ebd1 RNAi#3. (TIF) [file pgen.1006870.s018.tif]

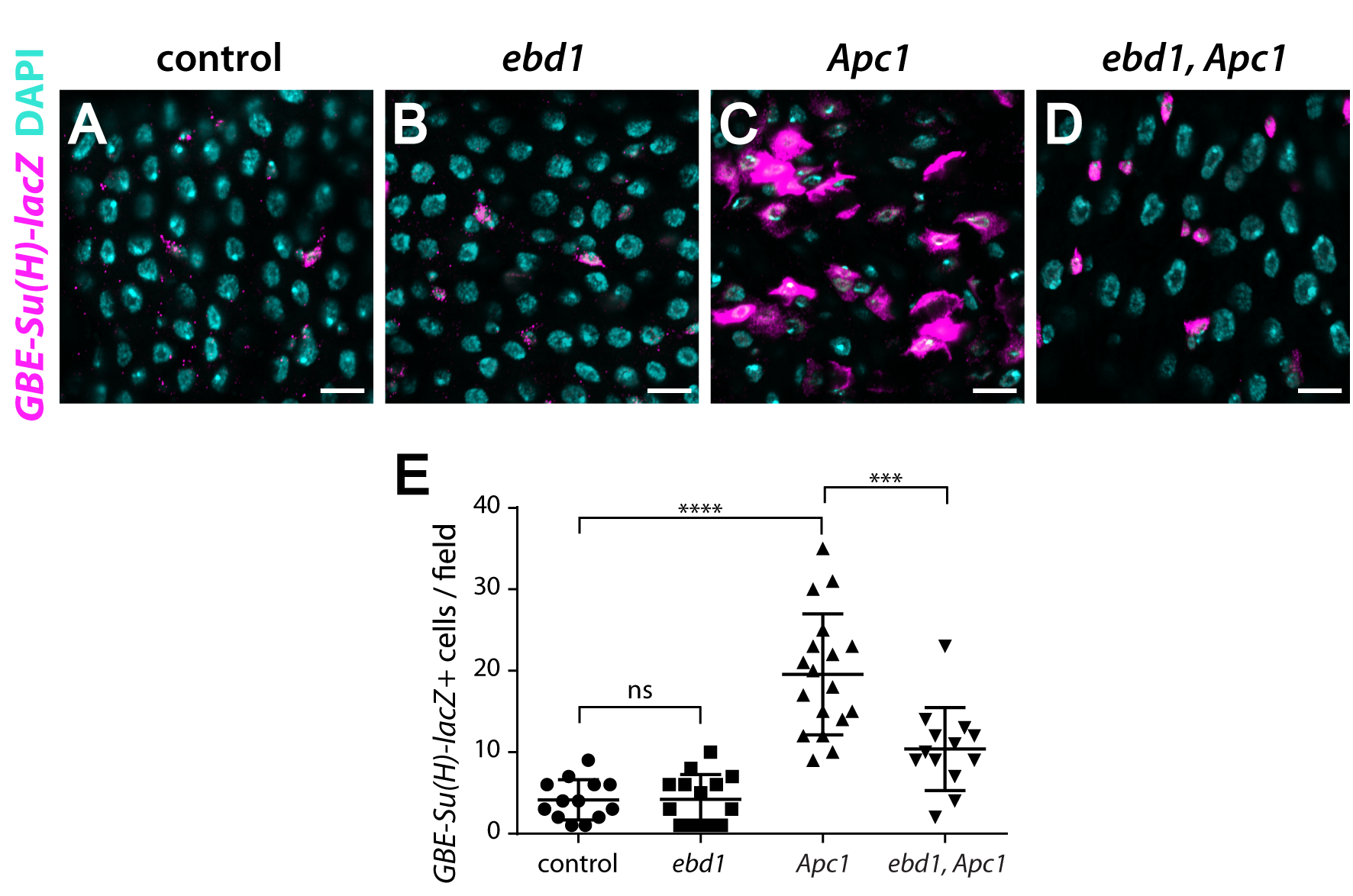

Supplement: S19 Fig — (A-D) Compared with controls (A), excess EBs (marked by GBE-Su(H)-lacZ, magenta) are readily detected in midguts of newly eclosed Apc1 mutants (C). In contrast, this defect is not observed in age-matched ebd1 mutants (B) and is suppressed in ebd1 Apc1 double mutants (D). Nuclei are labeled by DAPI (turquoise). (E) Quantification of GBE-Su(H)-lacZ positive cells in newly eclosed control, ebd1, Apc1 and ebd1 Apc1 mutants. **** P<0.0001, *** P<0.001, ns: not significant (t-test). Scale bars: (A-D) 10 μm. Genotypes: control: GBE-Su(H)-lacZ/+ ebd1: GBE-Su(H)-lacZ/+; ebd1240 Apc1: GBE-Su(H)-lacZ/+; Apc1Q8 ebd1 Apc1: GBE-Su(H)-lacZ; ebd1240 Apc1Q8. (TIF) [file pgen.1006870.s019.tif]
